# Prospects of Nanoscience with Nanocrystals: 2025 Edition

**DOI:** 10.1021/acsnano.5c07838

**Published:** 2025-09-03

**Authors:** Maria Ibáñez, Simon C. Boehme, Raffaella Buonsanti, Jonathan De Roo, Delia J. Milliron, Sandrine Ithurria, Andrey L. Rogach, Andreu Cabot, Maksym Yarema, Brandi M. Cossairt, Peter Reiss, Dmitri V. Talapin, Loredana Protesescu, Zeger Hens, Ivan Infante, Maryna I. Bodnarchuk, Xingchen Ye, Yuanyuan Wang, Hao Zhang, Emmanuel Lhuillier, Victor I. Klimov, Hendrik Utzat, Gabriele Rainò, Cherie R. Kagan, Matteo Cargnello, Jae Sung Son, Maksym V. Kovalenko

**Affiliations:** † 148492 Institute of Science and Technology Austria (ISTA), Am Campus 1, 3400 Klosterneuburg, Austria; ‡ Institute of Inorganic Chemistry, Department of Chemistry and Applied Biosciences, 27219 ETH Zürich, 8093 Zürich, Switzerland; § Laboratory of Thin Films and Photovoltaics, Empa−Swiss Federal Laboratories for Materials Science and Technology, 8600 Dübendorf, Switzerland; ∥ Laboratory of Nanochemistry for Energy (LNCE), Department of Chemical Sciences and Engineering, 150727 École Polytechnique Fédérale de Lausanne, CH-1950 Sion, Switzerland; ⊥ Department of Chemistry, 27209 University of Basel, 4058 Basel, Switzerland; # McKetta Department of Chemical Engineering, 12330 The University of Texas at Austin, Austin, Texas 78712, United States; ∇ Department of Chemistry, University of Texas at Austin, Austin, Texas 78712, United States; ○ Laboratoire de Physique et d’Etude des Matériaux, 52883 ESPCI-Paris, PSL Research University, Sorbonne Université Univ Paris 06, CNRS UMR 8213, 10 rue Vauquelin, 75005 Paris, France; ◆ Department of Materials Science and Engineering, and Centre for Functional Photonics (CFP), City University of Hong Kong, 83 Tat Chee Avenue, Kowloon, Hong Kong, SAR 999077, P. R. China; ¶ Catalonia Institute for Energy Research − IREC, Sant Adrià de Besòs, Barcelona 08930, Spain; †† ICREA, Pg. Lluís Companys 23, 08010 Barcelona, Spain; ‡‡ Chemistry and Materials Desing Group, Institute for Electronics, Department of Information Technology and Electrical Engineering, ETH Zurich, Gloriastrasse 35, CH-8092 Zurich, Switzerland; §§ 7284 University of Washington, Box 351700, Seattle, Washington 98195-1700, United States; ∥∥ Univ. Grenoble Alpes, CEA, CNRS, IRIG-SyMMES, STEP, 38000 Grenoble, France; ⊥⊥ Department of Chemistry, James Franck Institute, and Pritzker School of Molecular Engineering, 2462 University of Chicago, Chicago, Illinois 60637, United States; ## Zernike Institute for Advanced Materials, 332143 University of Groningen, Nijenborgh 3, Groningen 9747AG, The Netherlands; ∇∇ Physics and Chemistry of Nanostructures, Department of Chemistry, Ghent University, B-9000 Gent, Belgium; ○○ Center for Nano- and Biophotonics, Ghent University, B-9052 Gent, Belgium; ◆◆ BCMaterials, Basque Center for Materials, Applications, and Nanostructures, UPV/EHU Science Park, Leioa 48940, Spain; ¶¶ 518636 Ikerbasque Basque Foundation for Science, Plaza Euskadi 5, Bilbao 48009, Spain; ††† Department of Chemistry, 1771 Indiana University, Bloomington, Indiana 47405, United States; ‡‡‡ State Key Laboratory of Coordination Chemistry, School of Chemistry and Chemical Engineering, 12581 Nanjing University, Nanjing 210023, China; §§§ Department of Chemistry, Center for BioAnalytical Chemistry, Key Laboratory of Bioorganic Phosphorus Chemistry & Chemical Biology (Ministry of Education), 12442 Tsinghua University, Beijing 100084, China; ∥∥∥ Sorbonne Université, CNRS, Institut des NanoSciences de Paris, 4 Place Jussieu, 75005 Paris, France; ⊥⊥⊥ Nanotechnology and Advanced Spectroscopy Team, C-PCS, Chemistry Division, 5112 Los Alamos National Laboratory, Los Alamos, New Mexico 87545, United States; ### Department of Materials Science and Engineering, 6429 Stanford University, 496 Lomita Mall, Palo Alto, California 94305, United States; ∇∇∇ Electrical and Systems Engineering, 6572 University of Pennsylvania, Philadelphia, Pennsylvania 19104, United States; ○○○ Department of Chemistry, University of Pennsylvania, Philadelphia, Pennsylvania 19104, United States; ◆◆◆ Department of Materials Science and Engineering, University of Pennsylvania, Philadelphia, Pennsylvania 19104, United States; ¶¶¶ Department of Chemical Engineering and SUNCAT Center for Interface Science and Catalysis, Stanford University, Stanford, California 94305, United States; †††† Department of Chemical Engineering, 34995 Pohang University of Science and Technology (POSTECH), Gyeongsangbuk-do 37673, Republic of Korea; ‡‡‡‡ SKKU Institute of Energy Science and Technology (SIEST), Sungkyunkwan University (SKKU), 2066, Seobu-ro, Jangan-gu, Suwon, Gyeonggi-do 16419, Republic of Korea

**Keywords:** nanocrystal, quantum dot, semiconductor, assembly, optoelectronics, catalysis, perovskites, synthesis, surface chemistry, photolithography, photonics, fluorescence, lasing, quantum light, thermoelectrics, high-entropy alloy, hard ceramics

## Abstract

Nanocrystals (NCs) of various compositions have made important contributions to science and technology, with their impact recognized by the 2023 Nobel Prize in Chemistry for the discovery and synthesis of semiconductor quantum dots (QDs). Over four decades of research into NCs has led to numerous advancements in diverse fields, such as optoelectronics, catalysis, energy, medicine, and recently, quantum information and computing. The last 10 years since the predecessor perspective “Prospect of Nanoscience with Nanocrystals” was published in ACS Nano have seen NC research continuously evolve, yielding critical advances in fundamental understanding and practical applications. Mechanistic insights into NC formation have translated into precision control over NC size, shape, and composition. Emerging synthesis techniques have broadened the landscape of compounds obtainable in colloidal NC form. Sophistication in surface chemistry, jointly bolstered by theoretical models and experimental findings, has facilitated refined control over NC properties and represents a trusted gateway to enhanced NC stability and processability. The assembly of NCs into superlattices, along with two-dimensional (2D) photolithography and three-dimensional (3D) printing, has expanded their utility in creating materials with tailored properties. Applications of NCs are also flourishing, consolidating progress in fields targeted early on, such as optoelectronics and catalysis, and extending into areas ranging from quantum technology to phase-change memories. In this perspective, we review the extensive progress in research on NCs over the past decade and highlight key areas where future research may bring further breakthroughs.

## Graphical abstract

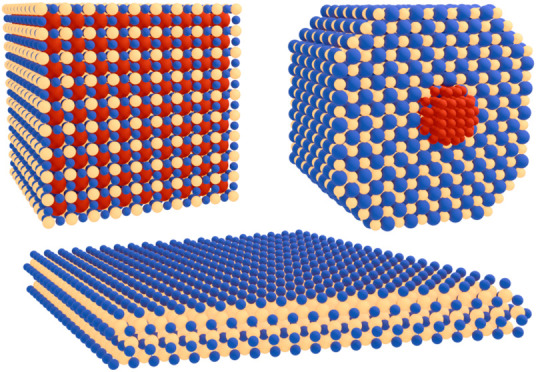

Colloidal nanocrystals (NCs) are tiny, crystalline particles typically ranging from 1 to 100 nm in size. These nanocrystalline grains are synthesized using surfactants to dissolve the precursors, direct the synthesis, and provide colloidal stability. Reducing the crystalline domain to the nanoscale results in their exceptional properties, including precise tunability of their optical, electronic, and chemical characteristics by altering the size, shape, and composition of the NCs. This versatility makes NCs powerful building blocks for creating materials and devices, revolutionizing various fields, from optoelectronics and catalysis to medicine and quantum computing.

The NC research impact was prominently recognized in 2023 when the Nobel Prize in Chemistry was awarded for the discovery and development of synthetic techniques of semiconductor quantum dots (QDs), where the reduced dimensionality induces quantum confinement effects and yields outstanding luminescent properties.^1^ This honor not only emphasized the magnificent achievement of the pioneers in the field but also the depth and breadth of research that has unfolded over the decades, well beyond QDs.

One of the defining features of NC research is its interdisciplinary nature. Scientists from diverse backgrounds, including chemistry, physics, materials science, and engineering, have contributed to advancing the field. This collaborative spirit has been instrumental in overcoming complex challenges, such as achieving precise control over NC synthesis, understanding NC surface chemistry, and integrating NCs into devices. The author list of this perspective reflects the previous statement: researchers in NCs from diverse backgrounds and focus areas have come together to highlight the significant progress made in recent years, while humbly acknowledging that the discussion is not fully comprehensive or exhaustive. Similar to its predecessor ACS Nano 2015 Perspective “Prospects of Nanoscience with Nanocrystals”,^2^ this article is structured into five chapters. The first chapter surveys the latest developments in NC synthesis. Subsequently, we delve into the intricacies of NC surface chemistry and explore diverse strategies to assemble NCs and spotlight emerging applications. We conclude by outlining possible future trajectories of this vibrant field.

## Synthesis

### Molecular-Level Insight into NC Formation

Over the past 10 years, one of the emerging trends in nanochemistry has been the increased attention dedicated to developing a molecular-level understanding of the mechanisms behind the nucleation and growth of NCs. A variety of *in situ* techniques are exploited to achieve this aim in various studies across the literature. These studies prove that a molecular-level understanding is crucial to driving the field of nanochemistry forward. Indeed, a more rational approach to synthesis results from the collected chemistry insight, which eventually results in superior sample homogeneity, size monodispersity, shape tunability, and compositional control.

*In situ* optical spectroscopy continues to be a valuable tool for mechanistic studies on QDs. For example, the entire pathway from precursor to CsPbBr_3_ QDs was elucidated using mostly this technique (Figure 1A).^3^ Akkerman et al. discovered that trioctylphosphine oxide (TOPO) plays a crucial role in the synthesis as it drives the PbBr_2_:Cs­[PbBr_3_] equilibrium toward the formation of the CsPbBr_3_ QDs. Having learned this, they synthesized highly monodisperse rhombicuboctahedral CsPbBr_3_ QDs, with a mean size tunable between 3 and 13 nm, with 100% precursor-to-QD conversion yield. Because of their superior monodispersity, these CsPbBr_3_ QDs, as well as FAPbBr_3_ and MAPbBr_3_ QDs obtained analogously, exhibited up to four well-resolved excitonic transitions, which had never been achieved before.

**Figure 1:**
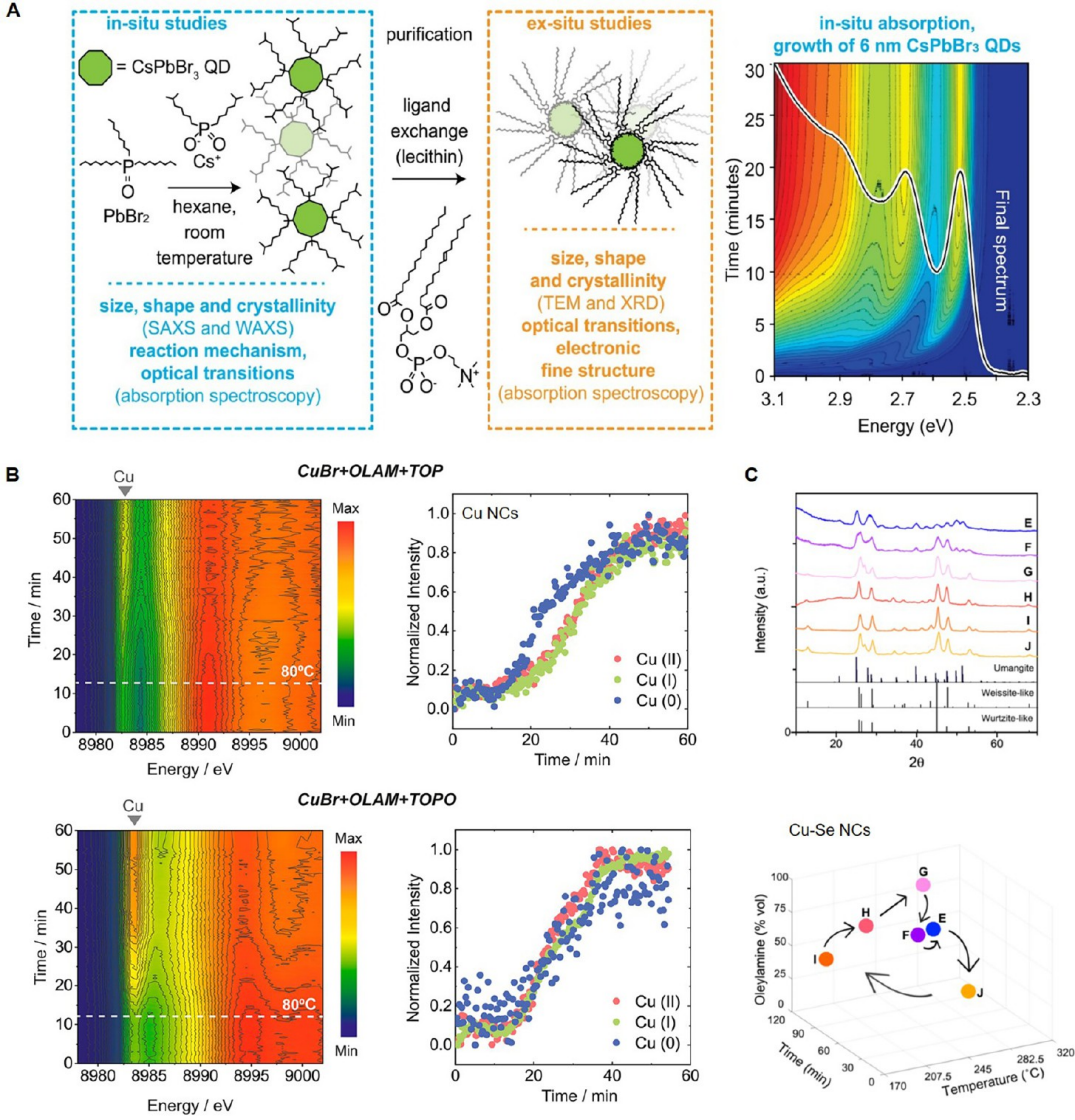
Examples of mechanistic understanding of NC formation. (A) CsPbBr_3_ QDs: Reaction scheme for their synthesis along with an overview of *in situ* monitoring techniques, complementary *ex situ* techniques on purified QDs, and one example of *in situ* recorded absorption spectra of 6 nm QDs during 30 min reaction (from left to right). Reproduced with permission from ref 3. Copyright © 2022 The American Association for the Advancement of Science. (B) Cu NCs: Color maps of normalized X-ray absorption near-edge structure spectra (left) and intensities of the Cu(0), Cu­(I), and Cu­(II) pre-edges (right) collected during their synthesis with CuBr and TOP or TOPO, leading to the formation of cubes and spheres, respectively. Reproduced from ref 4. Copyright © 2019 American Chemical Society. (C) Cu–Se NCs: Powder X-ray diffraction (XRD) patterns of phase combinations of copper selenide NCs (left) that result from the reaction conditions reported in a 3D map (right) wherein Cu­(oleate)_2_ and Ph_2_Se_2_ are the precursors. The coded letters represent the following phase combinations: (E) umangite Cu_3_Se_2_, (F) wurtzite-like Cu_2–*x*_Se/umangite Cu_3_Se_2_, (G) wurtzite-like Cu_2–*x*_Se, (H) weissite-like Cu_2–*x*_Se/wurtzite-like Cu_2–*x*_Se, (I) weissite-like Cu_2–*x*_Se, and (J) weissite-like Cu_2–*x*_Se/umangite Cu_3_Se_2_. Reproduced from ref 21. Copyright © 2023 American Chemical Society.

Meanwhile, *in situ* X-ray absorption and scattering are widespread in the community as they are applicable to a larger variety of materials compared to optical spectroscopies and offer unique insight into the chemical and structural evolution of species in the reaction from precursors to the final NCs. In one example, *in situ* X-ray studies on copper NCs have highlighted the crucial importance of precursor chemistry in determining the shape. Strach et al. discovered that complexes forming between CuBr and trioctylphosphine (TOP) or TOPO disproportionate at different rates, and this rate of disproportionation governs the monomer flux, thus the final shape of the obtained NCs (Figure 1B).^4^ Thanks to this acquired knowledge, Strach et al. synthesized size-tunable spheres, octahedra, cubes, and also tetrahedra, which were not previously available. More recently, the substitution of TOP with diphenylphosphine enabled a continuous shape modulation from single-crystalline to twinned and stacking fault-lined Cu NCs.^5^ *In situ* X-ray absorption spectroscopy evidenced the formation of a copper­(I)­bromide–diphenylphosphine complex with higher reactivity compared to the copper­(I)­bromide–TOP complex. This higher reactivity broadens the temperature range accessible for the synthesis and, thus, the attainable shapes. Complementing X-ray absorption with X-ray scattering methods, Mantella et al. found that copper spheres form from lamellae of a copper phosphonate coordination polymer.^6^ A follow-up study demonstrated that the length of the phosphonic acid utilized during the synthesis regulates the thermal stability of the polymer lamellae.^7^ Using shorter carboxylic chains generates more thermally stable lamellae, which could template 2D copper sheets. These studies on copper NCs are significant because enlarging the current library of shapes of these NCs and, more broadly, nonprecious metal NCs is particularly relevant to selectively direct catalytic transformations.^8^

In a second example, time-resolved X-ray scattering techniques, including small-angle, wide-angle, and total scattering measurements with pair distribution function analysis, were coupled with *in situ* absorbance to provide insight into the formation of PbS QDs, which are prominent visible light-absorbing QDs.^9,10^ Specifically, the conversion kinetics of a library of differently substituted thiourea, which is the sulfur precursor, were correlated to the number of NC in solution and the mean radius over time. The data evidenced that growth kinetics are size-dependent and determine the size monodispersity. Furthermore, Abécassis et al. and Campos et al. concluded that the nucleation of these particles is slow and continued, which contrasts with the classical idea of “burst of nucleation” proposed by La Mer.

Particularly relevant for the molecular-level understanding of NC formation are nuclear magnetic resonance (NMR) spectroscopy and mass spectrometry. Although these techniques have yet to be used *in situ*, they still provide important insight, especially when coupled with *in situ* investigation. To mention one example that further supports the idea of the absence of a nucleation event separated from the growth, the combination of mass spectrometry, *in situ* X-ray scattering, and diffraction, along with theoretical calculations, indicated that iron oxide NCs form via continuous growth of trinuclear-oxo iron clusters through the esterification of the oleate ligands induced by a long-chain alcohol.^11^ This insight has potential implications for the synthesis of other metal oxide NCs, which often do not exhibit the same size monodispersity and size tunability that has been demonstrated for iron oxide so far. In a second example, mass spectrometry combined with optical spectroscopy revealed quantized growth for InP QDs^12^ with well-defined clusters forming as reaction intermediates. Similar quantized growth has been shown for other systems (*e*.*g*. ZnSe^13^ and CdSe^14^). This mechanism again represents a deviation from the classical nucleation theory (CNT); notably, it suggests that understanding how these clusters convert into the final NCs is crucial for obtaining highly monodispersed QDs and narrowing the emission width.

These selected examples highlight that progress in NCs synthesis relies on insight into the chemistry behind their formation. The need for a more predictive synthesis of NCs away from a trial-and-error approach drives the efforts of the scientific community, which is further motivated by the ambitious idea of developing a retrosynthetic approach to NCs synthesis. A few examples of retrosynthesis do exist, wherein presynthesized NCs are used as precursors in solid-state reactions (*e*.*g*., cation exchange, thermally induced reactions) and converted into the targeted NC product via programmable reaction steps.^15,16^ However, this approach still relies on synthesizing the initial NC precursors, which might limit its applicability.

The complexity of colloidal NCs is certainly superior compared to that of organic molecules, as they can include almost all elements of the periodic table in their core; these atoms can be assembled in different crystalline structures, phases, compositions, sizes, and shapes. Additionally, surface organic ligands are integral elements along with the inorganic core, which must be considered. This complexity implies that the ability to write down balanced chemical equations from precursors to NCs is challenging, especially if the same reagent plays several roles that are difficult to decouple. Yet, a few promising examples have been reported.^17,18^

An alternative way to envision retrosynthesis for colloidal NCs is in the form of multidimensional maps where different parameters are simultaneously reported. In addition to experiments, a new theory of nucleation and growth is needed to move toward retrosynthetic design of colloidal NCs. This theory should include chemical models, including the reaction intermediates and transition states. A recent publication moves in this direction for the case of CdSe QDs, for which more knowledge exists compared to other classes of colloidal NCs.^19^ These retrosynthesis maps might eventually require the aid of machine learning algorithms. Efforts toward embracing the digital transformation in the colloidal synthesis of NCs are ongoing, with a few successful examples already being published.^20–22^ As one example, referring back to Cu NCs, a machine-learning toolbox that operates in a low data regime from electronic lab notebook data was recently proposed.^22^ The developed toolbox predicts the NC shape given the reaction conditions and proposes reaction conditions given a target NC shape. By classifying NC shapes on a continuous energy scale, an unreported shape, which is Cu rhombic dodecahedra, was synthesized along with the chemical knowledge regarding the use of different copper halides as precursors.

Eventually, these three different approaches to retrosynthesis of colloidal NCs might result in a general conceptual framework that describes reaction patterns common across different classes of materials. As for now, the scientific community must continue its effort toward a molecular-level understanding of the chemistry, along with appropriate data storage and sharing practices^23^ which are of fundamental importance for moving the field of nanochemistry toward an era wherein retrosynthesis is a reality rather than a utopia.

### Reaction Mechanism with a Focus on Oxides

NC formation can be split into precursor conversion (metal–organic chemistry) and crystallization. On the one hand, while many precursor conversion reactions have been elucidated, we have recently come to the realization that it is not only the precursors that are undergoing chemical transformations. In the last five years, it became clear that many commonly used solvents and ligands form side products that were previously not considered: (i) 1-octadecene polymerizes,^24^ (ii) nitrates oxidize amines to carboxylic acids,^25^ (iii) TOPO decomposes to phosphinic and phosphonic acids,^26^ (iv) oleylamine forms graphitic flakes,^27^ (v) oleic acid undergoes decarboxylative coupling to a ketone.^28^ Oleylamine is a particular problem, with the technical grade varying in composition,^29^ and with purification-dependent complexation strength.^30^ On the other hand, crystallization is commonly described by CNT. The very notion of nucleation was challenged for metal oxides. Oxo clusters and amorphous intermediates play an important role, as shown in several examples.

Iron oxide NCs can be formed by esterification of iron oleate with aliphatic alcohols. The iron oleate precursor is an oxo cluster, Fe_3_(μ_3_-O)­(OOCR)_7_, identified by matrix-assisted laser desorption ionization–time of flight (MALDI-TOF, Figure 2A).^11^

$$2\;Fe_{3}O{\left(\mathrm{OOCR}\right)}_{7}+{14\;R}^{\prime }\mathrm{OH}\overset{200^{{}^{\circ}}\;C}{\to }3\;Fe_{2}O_{3}+14\;\mathrm{RCOO}R^{\prime }+{7\;H}_{2}O$$

**Figure 2:**
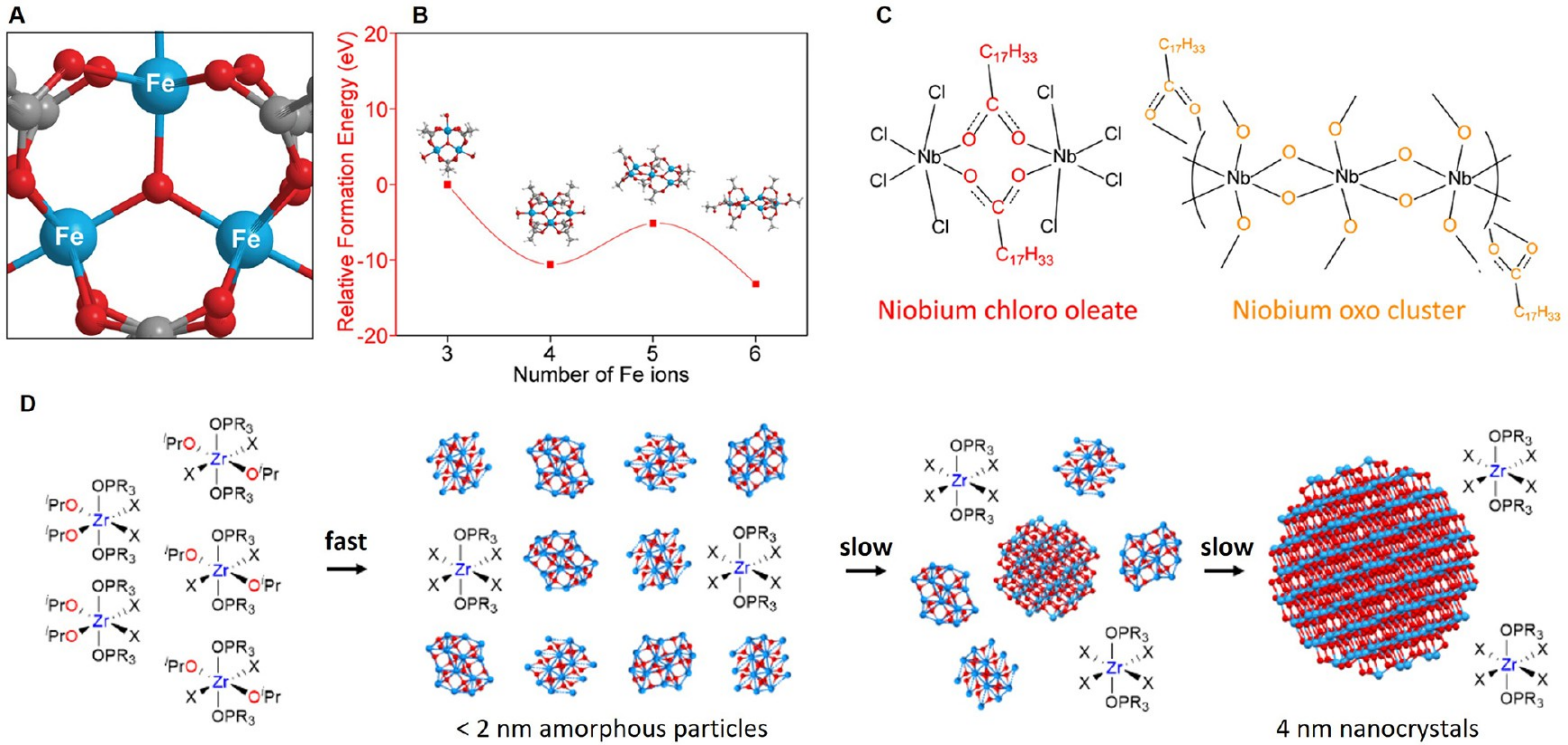
(A) Proposed structure of iron oleate. (B) Calculated relative formation energies of carboxylate-capped oxo clusters with different nuclearity. Panels (A) and (B) adapted from ref 11. Copyright © 2019 American Chemical Society. (C) Intermediates identified in the synthesis of niobium oxide. Adapted from ref 32. Copyright © 2020 American Chemical Society. (D) Zirconium halide alkoxides decompose into amorphous intermediates that turn into crystalline particles. Adapted from ref 36. Copyright © 2023 American Chemical Society.

Upon esterification at 200 °C, Fe_4_, Fe_5_, and Fe_6_ species are immediately detected without an induction phase. The term continuous growth was coined to describe the process in contrast to burst nucleation or continuous nucleation. Calculations show that there is indeed no barrier to growth when the carboxylate ligands are considered (Figure 2B). In contrast, CNT describes nucleation as an activated process with an energy barrier for nucleation due to the high surface energy of the nucleus. Carboxylate ligands thus strongly reduce the surface energy. Understanding the cluster nature of the precursor allowed also to understand the formation of metal ferrite, MFe_2_O_4_ NCs. Mn^2+^, Co^2+^, and Ni^2+^ ions form bimetallic oxo clusters with Fe^3+^: MFe_2_O­(oleate)_6_. The MFe_2_O_4_ NCs are readily formed from this precursor complex. In contrast, copper and zinc do not form mixed oxo clusters and thus not the metal ferrite NC.^31^

Niobium oxide nanoplatelets (NPLs) are synthesized from niobium chloride, oleic acid, and oleylamine at 300 °C.^32^ A chloro-carboxylate complex is formed upon the reaction of oleic acid and niobium chloride (Figure 2C). Synthesizing NCs from the chloride-containing complex produced nanorods (NRs) of the common orthorhombic phase of fully oxidized Nb_2_O_5_, which is also readily obtained by other methods. Only by first forming a niobium oxo cluster free from chloride complexation (Figure 2C), and then injecting this intermediate into the reaction flask were NPLs of the targeted monoclinic Nb_12_O_29_ phase produced. The oxo cluster is formed at 120 °C under vacuum by condensation of two oleic acid molecules, producing the anhydride and water. Water hydrolyzes the niobium chloride, liberating HCl, which is removed by evaporation.

$$NbCl_{x}{\left(OOCR\right)}_{5-x}+excess\;RCOOH\overset{120^{{}^{\circ}}\;C}{\to }NbO_{n}{\left(OOCR\right)}_{5-n/2}+xHCl↑+n{\left(RCO\right)}_{2}O$$

Although no specific structure of the oxo cluster intermediate could be identified, the metal oxo bridges were identified by Fourier transform infrared spectroscopy (FTIR) with supporting evidence for the chemical and structural identification provided by UV–vis (ultraviolet visible), elemental analysis, NMR, and dynamic light scattering. NPLs with a similar morphology and monoclinic Nb_12_O_29_ crystal phase could be formed from a niobium oxalate precursor and oleic acid, avoiding the potential for chloride coordination of the precursor and suggesting a similar cluster intermediate may be involved.^33^

Zirconium oxide NCs can be synthesized by reaction of zirconium chloride with zirconium isopropoxide isopropanol complex in TOPO at 340 °C.^34^ The actual precursor is a mixed zirconium chloro isopropoxide complex coordinated by two TOPO molecules (Figure 2D), as identified by ^31^P NMR spectroscopy.^35^ The precursors decompose according to an E1 elimination reaction, producing finally ZrCl_4_ as a byproduct, limiting the overall material yield to 50%.

$$2\;ZrCl_{2}{\left(OiPr\right)}_{2}\overset{340^{{}^{\circ}}\;C}{\to }ZrO_{2}+ZrCl_{4}+2\;\mathrm{HO}i\mathrm{Pr}+2\;propene$$

The precursor decomposes into many small (<2 nm) amorphous intermediates.^36^ After a single nucleation event, the crystalline particles grow by consuming the amorphous intermediates (Figure 2D). These observations were made through a combined small-angle X-ray scattering (SAXS) and pair distribution function (PDF) analysis of reaction aliquots. Kinetic modeling shows that the precursor decomposition is fast compared to the crystallization, leading to a buildup of amorphous intermediates. The particle size can be tuned by either extending the growth (fresh precursor injection) or by regulating nucleation through the precursor conversion rate.^35,36^ The presence of an amorphous intermediate is reminiscent of similar observations made in the case of CsPbBr_3_ NCs^37^ and the occurrence of an amorphous gel in the synthesis of HfO_2_ NCs.^38^ Also for CsPbBr_3_, the buildup of amorphous clusters was explained by the rate imbalance.

It is interesting that similar conclusions have been obtained in the studies of nucleation and growth, regardless of the covalency of the lattice or the chemical nature of the precursors. The classical picture of burst nucleation and diffusion-limited growth has been retired and seems to have been replaced by two types of crystallization. In the case of lead halides and oxides of tri-, tetra-, and pentavalent metals, cluster intermediates were identified.^11,32,36,37^ In the case of Pd, Ir, CdSe, InP, and PbS, continuous nucleation and a strongly size-dependent growth rate were observed.^9,10,39–43^

### Engineering Shapes of Metal Chalcogenide NCs

Modulating the shape of NCs stands as a well-established method for spectrally tuning their optical properties. Moving from QDs to NRs and NPLs, the quantum confinement undergoes a transition from 3Ds to 2Ds and, ultimately, one dimension (1D). In the case of NPLs, the thickness is reduced to just a few atomic planes, while lateral dimensions extend over hundreds to thousands of square nanometers.^44^ Unlike spherical particles, these 2D nanoparticles exhibit no inhomogeneous broadening, resulting in remarkably narrow optical properties. The PL full width at half-maximum can be as narrow as 1.5 *k*_B_ *T*.

Like other semiconductor NCs, the optical properties of NPLs can be tuned by adjusting their thickness. The initial demonstrations of NPLs were based on cadmium chalcogenides, with either a wurtzite or zinc blende (ZB) crystal structure. For ZB NPLs, the thickness was initially varied from 2.5 to 5.5 monolayers (MLs), where 1 ML represents the stacking of a cadmium and chalcogen plane in the [001] direction of the ZB crystal structure. In NPLs, the two external (001) wide planes are cadmium-based and are passivated by carboxylate ligands to ensure neutrality and colloidal stability. The growth of these anisotropic NPLs stems from a template growth method originally developed for wurtzite nanoparticles. While the growth of ZB NPLs is widespread, a consensus on their growth mechanism has not been reached. Historically, they were synthesized by introducing an acetate salt during the growth of NCs resulting from the reaction of Cd­(Oleate)_2_ or Cd­(Myristate)_2_ with a selenium precursor in octadecene.^45,46^ However, it has been demonstrated that these NPLs can be obtained without acetate salt under conditions where the reaction is kinetically limited.^47,48^ Alternatively, a two-step process involves the annealing of purified spherical particles in the presence of Cd­(Acetate)_2_.^49,50^ More recently, *in situ* SAXS has revealed the presence of very small anisotropic NCs at the early stages of synthesis whose growth is further promoted by the presence of acetate salts.^51^ Moreover, the ratio between different precursors or ligands appears to influence the presence of (101) or (111) facets, leading to the formation of either rectangular or square-shaped NPLs.^52^ Understanding the impact of ligands on the lateral extension of 2D particles remains a topic of high interest for controlling their thickness and shape.

Considering their substantial lateral extension and limited thickness, NPLs can be likened to flexible substrates for the self-assembly of molecules. Maintaining charge neutrality necessitates an average of one X ligand per Cd surface.^53^ The initial carboxylate ligands can undergo postsynthesis modifications with thiolates, phosphonates, halides, or even chiral cysteine ligands, resulting in circular dichroism.^54–61^ Alterations in surface chemistry induce changes in the confinement direction, leading to an evolution in optical properties.^54,62,63^ The observed bathochromic shifts in optical properties may arise from modifications in thickness direction stress, wave function delocalization over ligands, or changes in exciton binding energy.^54,56,63–65^

Carboxylate ligands induce in-plane tensile stress on NPLs, which can be alleviated by halide ligands or transformed into compressive stress with thiolates or phosphonates. This stress modification may accompany a change in NPLs’ shape, and to release elastic energy, they may fold into helical structures.^56,66,67^ This distinctive shape implies chirality, potentially resulting in circular dichroic optical properties.^68^ Additionally, halide ligands not only alleviate stress but also reduce surface energy, facilitating an increase in thickness.^69–72^ Consequently, in the presence of chloride, CdSe NPLs with thicknesses exceeding 5.5 MLs up to 11.5 MLs have been successfully synthesized.^69,72,73^

Initially, the control of NPLs’ optical properties centered on their thickness; however, diverse heterostructures can now be grown owing to their characteristic geometry. When NPLs are elongated perpendicular to the confined direction (*i*.*e*., core/crown configuration), the number of atomic planes in the thickness remains constant, resulting in a planar heterojunction. The lateral extension for both homo- and heterostructures ranges from a few nanometers to hundreds of nanometers. Specifically designed core/crown heterostructures have been proposed, facilitating the localization of electron and hole wave functions in distinct regions of the heterostructures based on the band alignment between different semiconductors. This approach allows for the violation of the Kasha rule, leading to NPLs with PL spectra featuring two or three distinctive emissions^74–76^ whose ratio between the emissions can be tuned by the excitation power (Figure 3). This is achieved by leveraging type I and type II band alignments between the semiconductors.^74,77–79^ By systematically rationalizing and engineering band alignments between semiconductors and their alloys and by determining the appropriate lateral extensions for these different components, 2D particles with multiple emissions can be synthesized on demand.^75^ Ultimately, once grown in thickness, NPLs with high QY are achieved, holding promise for potential applications in optoelectronic devices.^80^

**Figure 3:**
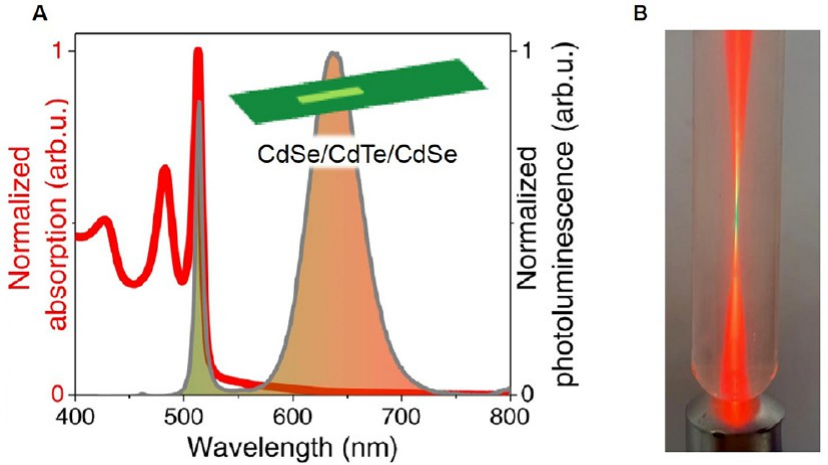
(A) Absorption and emission of 4.5 ML CdSe/CdTe/CdSe core/crown/crown NPLs. (B) Image of a test tube containing the NPLs, illuminated by a blue 405 nm laser diode. At the focal point, the emission is green while it is red away from it. Figure adapted with permission under CC BY 4.0 from ref 74. (Copyright © 2022 Dabard et al. open access).

Working with cadmium chalcogenides NPLs confines optical properties to the visible range. To expand these properties into the infrared (IR) range, different strategies have been proposed. One approach involves the direct synthesis of 2D lead chalcogenide nanoparticles,^81–84^ while another utilizes cation exchange to obtain mercury chalcogenide NPLs.^85–89^ The ultimate aim is to maintain narrow optical properties. Direct syntheses of ultrathin PbS NPLs have been documented, demonstrating optical properties reaching up to 750 nm with limited tunability.^84^ Consequently, increased efforts are now dedicated to refining their syntheses to achieve narrow, efficient, and tunable emissions within the telecommunication wavelengths.^82,90^

Cadmium chalcogenide NPLs serve as templates for cation exchange with various elements, including copper,^91–95^ lead,^96,97^ and mercury.^85,87,88^ In the case of lead cation exchange, while it proves to be efficient, the optical features are broadened compared to pristine particles. This is most likely due to a loss of 1D confinement, as TEM images reveal single particles with inhomogeneous contrast. Additionally, the emission broadening may result from compositional inhomogeneity, with either vacancies or residual Cd^2+^ cations remaining in the particles. Conversely, when cation exchange is applied to mercury chalcogenides NPLs, the 2D shape is maintained, and optical features comparable to those of visible NPLs are observed, with distinct transitions shifted by up to 900 nm with potential optoelectronic applications.^98–100^

### Mercury Chalcogenides

Mercury chalcogenide NCs,^101,102^ in particular HgTe, exhibit distinct properties due to quantum confinement effects at the nanoscale and have attracted a great deal of attention since the 1990s,^103,104^ as active material in optoelectronics as photodetectors,^105^ thermal imaging components,^106,107^ and light-emitting diodes (LEDs).^108,109^ In comparison to other IR active semiconductors, such as lead chalcogenides, gallium indium arsenide, silicon, *etc*., mercury chalcogenide QDs offer the broadest band gap tunability, from near-infrared (NIR) to terahertz range.^102^ Beyond size control to achieve quantum confinement, in the past decade, a significant effort has been made to tailor their electronic structure by inducing anisotropy in their shape along three (multipods), two (NPLs), or one (NRs) directions (Figure 4A). Theoretically, the large exciton Bohr radius of HgTe (40 nm)^110^ would allow those nanoparticles to be elongated up to tens of nanometers while still remaining in the strong quantum confinement regime. While branched nanostructures have found applications in NIR and short-wave infrared (SWIR) LEDs,^111^ the development of 2D and 1D elongated mercury chalcogenide nanostructures is still an ongoing research area.

**Figure 4:**
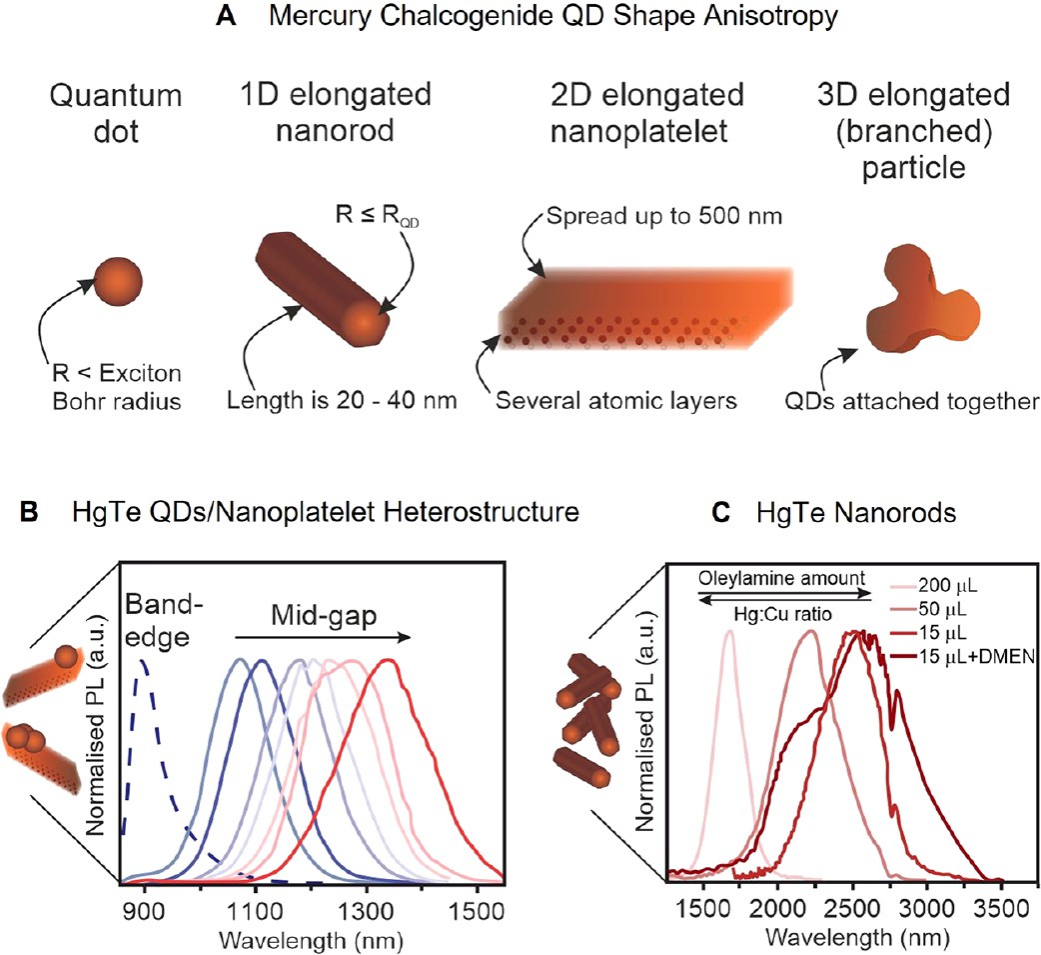
(A) Altering the morphology of the mercury chalcogenide QDs. (B) NIR emission of HgTe QDs/NPLs. Reproduced from ref 88. Copyright © 2020 American Chemical Society. (C) PL tunability of HgTe NRs, governed by the amount of ligand oleylamine and the resulting Hg:Cu ratio (DMEN is *N*,*N*-dimethylethylenediamine). Reproduced from ref 115. Copyright © 2023 American Chemical Society.

Pioneering work by S. Ithurria’s group^85^ demonstrated that the use of mercury-amine complexes during cation exchange reaction (CdTe to HgTe) can result in Cd-free, colloidally stable HgTe NPLs with narrow NIR emission bands. However, the softness of the final mercury chalcogenides often results in undesired etching, causing structural defects such as voids and torn edges. In such cases, proper control of the reaction parameters, as well as implementation of ternary HgSe_*x*_Te_1–*x*_ alloys or HgTe NPL/QD heterostructures is a key to obtaining NPLs with better-defined structures and bright emission covering the range from 1 to 1.5 μm (Figure 4B).^87,88^ Cation exchanges are diffusion-limited processes. For mercury chalcogenides, the diffusion of Hg^2+^ can be even more limited. For example, the CdSe-to-HgSe cation exchange is topotaxial, meaning that the guest and host crystalline phases are identical. Hence, it has been shown that for thick CdSe NPLs, the exchange was limited to a couple of atomic planes only, leading to the formation of core/shell NPLs and the absence of a bandgap redshift with increasing thickness.^86^ With heterostructures, the optical properties may be shifted up to 1.4 μm.^112,113^ More recently, chemical strategies have been developed to overcome this limitation and achieve NPLs emitting in the IR range thanks to the use of a joined Ag^+^ cation with Hg^2+^.^113,114^ Elongating HgTe QDs of 3 nm in diameter in one direction to form NRs allows for a significant shift of the emission band toward the midwave infrared (MWIR) region (Figure 4C).^115^ In this work, HgTe NRs were produced by introducing an intermediate step into the cation exchange reaction, namely, the transformation of CdTe NRs to Cu_2‑x_Te NRs and the latter ones to HgTe NRs. Notably, the formation of a stable wurtzite-structured HgTe NRs was achieved. Furthermore, it has been demonstrated that the addition of surfactants can enhance the extraction of Cd and Cu ions from the HgTe NRs while preserving their structural integrity. Proper purification from previous stages’ residuals allowed the tunability of the position of a narrow PL band in the spectral range from 1600 to 2600 nm (Figure 4C). While these HgTe NRs could be used as active media for field-effect transistors, the defect-rich crystal structure of the HgTe NRs still constitutes a limiting factor for an effective charge carrier transfer. Therefore, further synthetic development is required to advance the HgTe NRs-based photodetector’s figures of merit.

### Intermetallics

Metal and alloys are inevitable materials in nearly all modern technologies.^116^ Intermetallic compounds formed by combining two or more metals are extremely diverse, with over 25,000 unique intermetallic phases to date.^117^ Intermetallic materials excel in various applications, including hydrogen storage,^118^ ordered magnetism,^119^ thermoelectrics,^120^ and shape-memory alloys.^121^ Moreover, it is well known that the nanoscale dimensions can enhance the properties and provide distinct functionalities of metals and intermetallic materials, benefiting fields such as catalysis,^122,123^ plasmonics,^124^ and energy-storage applications.^125^ In strong dissonance, intermetallic NCs in the form of colloids remain an underexplored area of research.

While monometallic NCs have been perfected in size and shape control,^126–128^ bimetallic and intermetallic compositions generally suffer from undefined morphology or broad size distributions. The main reason behind this is the different reactivity of metals in the liquid-phase chemistry systems. It is challenging to match the conversion rates of two precursors, and therefore, only several bimetallic colloids have been prepared in excellent quality (*e*.*g*., CoPt_3_, FePt, and NiPt_3_ intermetallics or alloys of Au–Ag, Bi–Sb, and In–Sn).^129–133^ For example, FePt NCs have been synthesized by the cothermolysis of two precursors, Fe­(CO)_5_ and Pt­(acac)_2_, designed to have the same decomposition temperature.^131^

Seed-mediated approaches are more successful, offering two generalizable methods for intermetallic NCs: galvanic replacement reaction^134^ and, recently discovered, nanoscale amalgamation alloying.^135^ Galvanic replacement relies on an electrochemical reaction, in which the ions of less active metal (*e.g*. Au^3+^ salt) push the more active metal (*e.g*. Ag^0^) out of the seed. This approach is powerful, and it works well for metal couples with similar enough electrochemical potentials (*e.g*., noble metals).^129^ However, the disadvantage of galvanic replacement is that expensive seed metal is sacrificial in the process. Furthermore, the interdiffusion of metals is typically slow during the galvanic replacement process, leading to the accumulation of incoming metal at the surface of the seeds and, thus, phase segregation within bimetallic NCs, slow reaction kinetics, and ill-defined NC interfaces with high roughness.^134^

Recently, the nanoscale amalgamation reaction has been proposed as a generalizable method for colloidal intermetallic NCs.^135^ Starting from monometallic seeds (*e.g*., Ni, Cu, Pd, Ag, or Au), the method involves a thermal decomposition of metal-amides (*e.g*., Ga, In, or Zn amides) to dispatch low-melting metals to the surface of NCs. This is followed up by the amalgamation process, *i.e*., an efficient way of alloy formation, in which a liquid metal diffuses into solid metal, forming a bimetallic composition or intermetallic compound (Figure 5A). To sum up, the nanoscale amalgamation reaction is a convenient colloidal synthesis for high-quality intermetallic NCs, which can be employed for up to 1000 intermetallic phases as long as one metal is liquid at the reaction conditions (260–320 °C and ambient pressure).^135,136^ The nanoscale amalgamation reaction provides colloidal intermetallics with unprecedented monodispersity, composition control, and phase purity (Figure 5B–D). Due to the fast diffusivity of liquid metals, the amalgamation synthesis takes only a few minutes, thus minimizing detrimental mass transfer effects, such as Ostwald ripening. Consequently, intermetallic NCs can be prepared with excellent size uniformity and size control, both defined by the quality of monometallic seeds (Figure 5B,C). Moreover, the composition of intermetallic NCs can be conveniently controlled by the amount of metalamide. Adjusting the amide concentration as well as kinetic parameters of the reaction (*i.e*., injection and growth temperature, reaction time), the amalgamation alloying can be optimized to provide intermetallic compounds with excellent composition uniformity (Figure 5B), access to different intermetallic compounds within the same bimetallic, and even achieve an accurate composition control within the solid solutions of those intermetallic phases (Figure 5D).^136^

**Figure 5:**
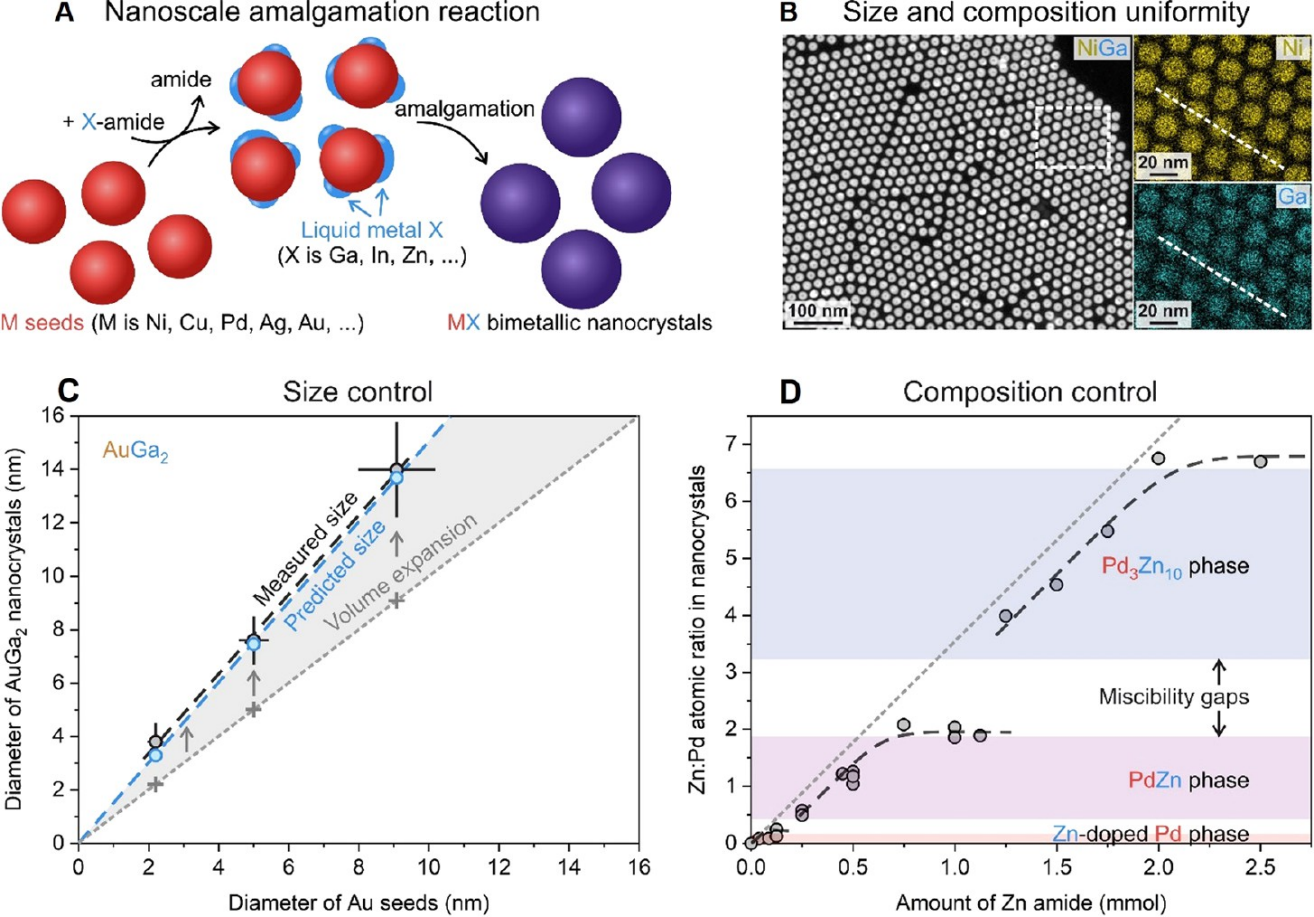
(A) Schematics of the nanoscale amalgamation reaction. Examples of excellent structural assets of intermetallic NC products, such as the size uniformity and composition homogeneity in NiGa (B), size control in AuGa_2_ NCs (C), and composition control in Pd–Zn NCs (D). Panels (A) and (D) adapted with permission from ref 136. (Copyright © 2024 Yarema et al.). Panels (B) and (C) are adapted from ref 135. Copyright © Clarysse et al., some rights reserved; exclusive licensee AAAS. Reprinted with permission from AAAS.

Combining metals at the nanoscale is a powerful strategy for many applications.^124,132,136–139^ Featuring size- and shape-dependent properties, large surface area, small hydrodynamic radius, and the ability to grow a protective oxide shell, colloidal intermetallics can be regarded as the most multifunctional class of nanomaterials. Such multifunctionality of intermetallic NCs has been recently exemplified for Pd–Zn bimetallic NCs prepared via the nanoscale amalgamation reaction.^136^ Depending on Zn amount and intermetallic phase, Pd–Zn NCs exhibit extended lifetime for Zn-ion batteries, improved stability as high-voltage cathodes, and superior performance in electrocatalytic O_2_ reduction. Finally, intermetallic NCs can be covered by a thin oxide shell, using controlled dealloying via air oxidation,^135^ mild oxidation agent,^140^ or electrochemical reaction.^141^ The oxide-protected materials exhibit improved stability at harsh conditions. For example, Au/Ga_2_O_3_ core/shell NCs preserve plasmonic properties even at elevated temperatures of 150 °C.^140^ In another paper, the Sb/Li_2_O core/shell NCs act as an ultimately durable Li-ion battery anode due to the delithiation/lithiation cycles via void formation and refill.^141^

### High-Entropy Materials (HEMs)

In the most general sense, HEMs are defined as single-phase compounds containing five or more principal elements with some degree of random occupation of at least one atomic sublattice (Figure 6). HEMs are an extension of the high-entropy alloy (HEA) concept introduced by Yeh et al. in 2004 when exploring the stabilization of multielement solid solutions through the configurational entropy contribution to the total Gibbs free energy.^142^ They estimated that the configuration entropy associated with the combination of 5 elements in equiatomic ratios might be sufficient to overcome the enthalpy of formation of most intermetallic phases, thus stabilizing the solid solution. Consequently, HEAs were initially defined by Yeh et al. as alloys composed of five or more principal elements, with the concentration of each element being between 35 and 5 atom %. While subsequent research has suggested that entropy might not be the primary factor in the formation, stabilization, and property determination of most proposed HEMs,^143–145^ and actually some of them have relatively low configuration entropies,^146,147^ the catchy name coined by Yeh et al. has rooted deep enough to prevail and be extended to virtually all types of materials.^143,145,146,148,149^

**Figure 6:**
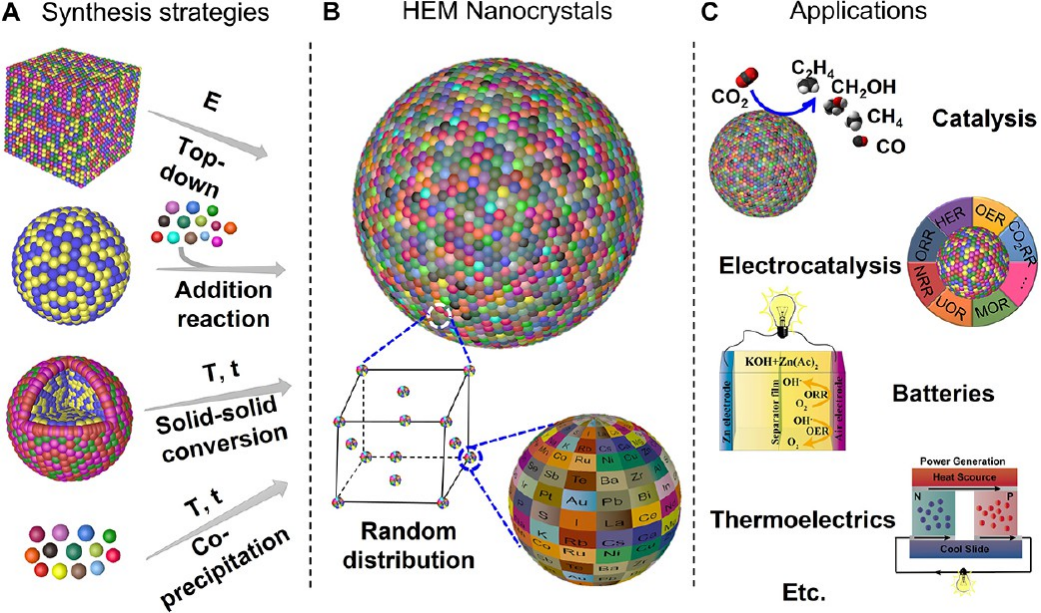
Scheme of the synthesis of HEM NCs by top-down, conversion, and bottom-up approaches (left), their vast compositional flexibility (center), and some of their potential applications (right).

Following the developments of bulk HEMs, in the past few years, HEM NCs have been synthesized and applied in a broad range of fields, including catalysis,^150–156^ (electro)­catalysis,^157–163^ energy conversion^164^ and storage,^165–167^ and biosensing,^168^ among others (Figure 6).^145^ Initial HEM NCs were produced using top-down and solid-state methods such as mechanochemical approaches and thermal shock strategies aiming at the ultrafast heating of the precursors to very high temperatures for a short time, followed by rapid cooling to trap the entropy-stabilized phase.^169–174^ More recently, several solution-based strategies have been developed to synthesize HEM NCs. Among other advantages, solution-based approaches allow for more efficient atomic-scale mixing of various elemental precursors without the high temperatures typically imposed by solid-state techniques.^175^ The developed solution-based methods include both templated and bottom-up approaches (Figure 6). The former involves either the annealing of heterostructured NCs such as core–shells to mix all the elements when enough energy for atomic diffusion and reorganization is provided,^176,177^ or the incorporation of additional elements in lower-component NCs,^178^ frequently through galvanic replacement^179^ or ion exchange processes driven by favorable redox potentials or solvation/desolvation energies.^168,180^ On the other hand, two main visions have realized the synthesis of unsupported HEM NCs using bottom-up approaches.

Earlier attempts to synthesize HEM NCs mirrored the approaches used to produce bulk HEMs and supported HEM NCs, like carbothermal reduction.^145,176,181,182^ It was believed that for the successful synthesis of HEM NCs, all reagents had to be reacted or reduced together. This approach requires the use of suitable precursors amenable to coreaction or coreduction, along with strong reducing agents or high temperatures, often involving the hot injection of either the precursor or the reductant.^176,181,182^ One of the pioneering colloidal synthesis methods in this direction was developed by Singh and Srivastava, who produced CrFeCoNiCu NCs in a mixture of oleic acid and oleylamine using lithium triethylborohydride as a strong reducing agent to enable the simultaneous incorporation of all elements into the growing NCs.^181^

Nevertheless, as previously observed in the synthesis of quaternary chalcogenide NCs,^183,184^ HEM NCs frequently form not from HEM nuclei but from the successive incorporation of the different elements into seeds that are rich in one of two of the components. In this direction, Iversen and Schaak groups reported how, in the synthesis of RuRhPdIrPt, RhPdIrPtSn, and NiRhPdIrPt NCs, Pd-rich seeds are initially formed, which afterward incorporate the other elements from the surface inward.^185–187^ This heterogeneous mechanism does not require precursors to have identical reduction potentials or reactivities.^158^ According to Dey et al. and Liu et al., a key synthesis parameter to obtain HEM NCs is a slow injection of a dilute precursor mixture to minimize the frequency of collisions between atoms of the same elements, thus avoiding the formation of secondary phases while maximizing the frequency of collisions between atoms of different elements to form the HEM.^158,165,166,186–189^

Overall, the colloidal synthesis of HEM NCs is still in its embryonic stage, facing numerous challenges. One major issue is the control of the NC shape and size, which, despite a few notable exceptions,^158,164,168,190^ remains elusive, especially for noble metal-free HEMs.^187,191^ Additionally, there has been a lack of effort in tuning the composition within each developed system, which is crucial for unlocking the full potential of these materials.^187,191^ Besides, there is a need to effectively manage defects and dopants within these complex materials, an area that has been seldom explored,^153^ and to produce HEM-based heterostructured materials. Overcoming these challenges strongly relies on improving our understanding of the mechanisms of formation of the HEM NCs and of how we can tune their nucleation and growth.^145^

The factors determining the formation of HEM NCs instead of NCs with several different phases are unclear, but entropy seems to play a rather secondary role. At the relatively low temperatures used in the solution synthesis of HEM NCs, the contribution of the configuration entropy to the total free energy is relatively small compared with formation enthalpies, precursor redox potentials, and surface/interface energies. Nevertheless, in some compounds, the formation of single-phase quinary NCs has been demonstrated to be more feasible than the formation of single-phase binary NCs. For example, using single-source precursors, Pittkowski et al. noticed that single-phase HEM NCs were easier to obtain than single-phase NCs with smaller constituents, even when the elements in the five-metal precursor reduced stepwise.^192^ To explain this experimental observation, they proposed the formation of HEM NCs to be kinetically controlled assuming that in a mixture of several elements, the concentration of each atom type is diluted as the number of elements increases. Therefore, despite the possible elemental, binary, ternary, and quaternary combinations increasing with the number of elements, it is also increasingly difficult for elements that would form secondary phases to find each other, which favors the formation of single-phase NCs containing all the elements. This hypothesis aligns with the synthesis of HEM by stepwise injection of precursor in the bottom-up synthesis. Nevertheless, while kinetics certainly plays a key role in HEM NC formation and in determining their size, shape, and composition distribution, the variety of HEM NC formation paths suggests more complex mechanisms at play.^145^ The surface/interface energies, precursor chemical reactivities,^186^ and mutual miscibility^193^ of elements may play a particularly important role in the formation of HEM NCs, although examples of HEM NCs that include immiscible combinations have also been reported.^194^

At the same time, the atomic distribution within HEM NCs is unlikely to be fully random. A preferential bonding between particular elements generating short-range order has been experimentally observed and theoretically predicted in several HEMs.^192,195^ This short-range order may play a fundamental role in defining the functional properties of the material. However, its characterization within HEM NCs is exceptionally challenging, complicating the establishment of reliable structure/composition–property relationships.^177,192^

The surface composition of HEM NCs might be particularly far from that of the overall particle due to the different chemical environments. This may have a particularly important effect on catalytic applications.^177^ Additionally, the surface atomic organization and overall composition are particularly susceptible to variations in the NC environment and the application conditions. In this direction, the oxidation and reduction of HEA NCs have resulted in a reorganization of the different elements,^196–199^ following similar trends to those observed in binary and ternary alloys, and associated with the relative oxophilicity of the different elements.^200–202^

Beyond the controversial role of entropy in stabilizing HEMs and defining their properties, the main interest of HEMs resides in the enormous range of compounds that can be generated by combining five or more elements of the periodic table in similar ratios. Considering 40 useful elements in the periodic table, over 650,000 different possible combinations of 5 elements and over 1.2 billion combinations of 5–10 elements exist. If further considering the different possible elemental ratios within each combination of elements, the number of possible materials becomes virtually infinite.^203^ The availability of this virtually unlimited pool of materials opens avenues for the design and engineering of HEM NCs, potentially made of abundant and nontoxic elements, to fulfill the requirements of a wide variety of applications. The combination of multiple elements also allows the development of multifunctional materials.

The exploration of the immense number of possible alloys is both the main interest of the research in HEMs and also its major challenge. The examination of the vast range of possible compositions requires high-throughput computational methods to predict stable HEM compositions and structures and their properties,^204–207^ extensive experimental synthesis and property/performance screening,^208^ and establishing reliable composition/structure–property correlations.

### III–V NCs

The polarity of chemical bonds plays an important role in defining semiconductor properties. For example, materials with nonpolar covalent bonds, such as Si and Ge, tend to have indirect band gaps, while the materials with highly ionic bonding, like ZnSe or CsPbX_3_ (X = Cl, Br, I), are less resistant to bond-angle deformation and more easily incorporate defects, which amplify device degradation rates.^209^ The profound importance of III–V semiconductors for optoelectronic applications follows from their ability to balance these competing trends. On the one hand, they are polar enough to exhibit direct bandgaps, with strong absorption and fast radiative recombination. On the other hand, they are sufficiently covalent to not suffer from the intrinsic bond lability typical of highly ionic crystals. Strong bonding makes III–V semiconductors generally more robust under high temperatures, intense illumination, and strong electric fields.^209,210^ For these reasons, today’s most efficient and highest power semiconductor light-emitting diodes and lasers are based on GaAs and GaN; moreover, because of their ability to form epitaxial heterostructures with precisely engineered electronic structure, III–V semiconductors are used in multijunction solar cells,^211^ quantum-cascade lasers,^212,213^ and many other devices.

Despite their tremendous usefulness, progress in the solution-phase synthesis of colloidal III–V NCs has been challenging. It contrasts starkly with the exceptional level of control achieved in the II–VI and IV–VI systems. Part of this challenge is due to the increased covalency of III–V compounds relative to II–VI and IV–VI systems,^214^ making the bonds hard to make due to the corresponding covalency of the precursor molecules, and hard to break. This increased covalency puts III–V compounds in the “nanoceramics” category, with greater similarities to carbides and borides than to chalcogenides. A second challenge has been the lack of suitable precursors for group III and V elements. However, four developments from the past decade have enabled significant advances in the synthesis and application of colloidal III–V NCs: atomically precise clusters, aminopnictogen precursors, molten inorganic salts as reaction media, and engineered shells.

The realization that atomically precise clusters exist as intermediates on the reaction landscape between III–V precursors and NCs has shed light on the mechanisms of III–V crystallization (Figure 7A). Syntheses that use clusters as single-source precursors or seeds typically result in monodisperse III–V NCs. In addition, these clusters can be isolated and understood from both structure and reactivity perspectives, creating opportunities for synthetic development. In 2015, Cossairt and co-workers re-examined the classic InP synthesis^220^ involving the reaction of indium carboxylates with P­(SiMe_3_)_3_ and discovered that this reaction proceeds through the intermediacy of a distinct cluster intermediate with absorption at 386 nm.^12^ In fact, this cluster had been documented earlier in the work of Peng.^221^ Subsequent work revealed the structure of this cluster, which was characterized by single crystal XRD as having a formula of In_37_P_20_(O_2_CR)_51_, and an inorganic core that deviates from the bulk ZB lattice and is perhaps best described as pseudowurtzite in character.^215^ Therefore, partial dissolution and rearrangement is necessary when this cluster is used as a precursor in thermolysis or seeded growth reactions. It has also been discovered that formation and dissolution of In_37_P_20_ proceeds through another higher-symmetry, but still pseudowurtzite, intermediate that was recently characterized by single crystal XRD with the formula In_26_P_13_(O_2_CR)_39_.^216^ Additional work has suggested that several other InP clusters may be accessible by changing ligands (*i.e*., carboxylates, amines, phosphonates, halides) and other additives (*i.e*., zinc) in the synthesis.^222,223^ While clusters have been studied and characterized most extensively for the InP system, it is clear that cluster intermediates are also central to the synthesis of InAs NCs.^224–227^ A particular challenge of clusters that must be designed around is they can create kinetic traps that hamper precursor-controlled reactivity during III–V NC nucleation and growth.

**Figure 7:**
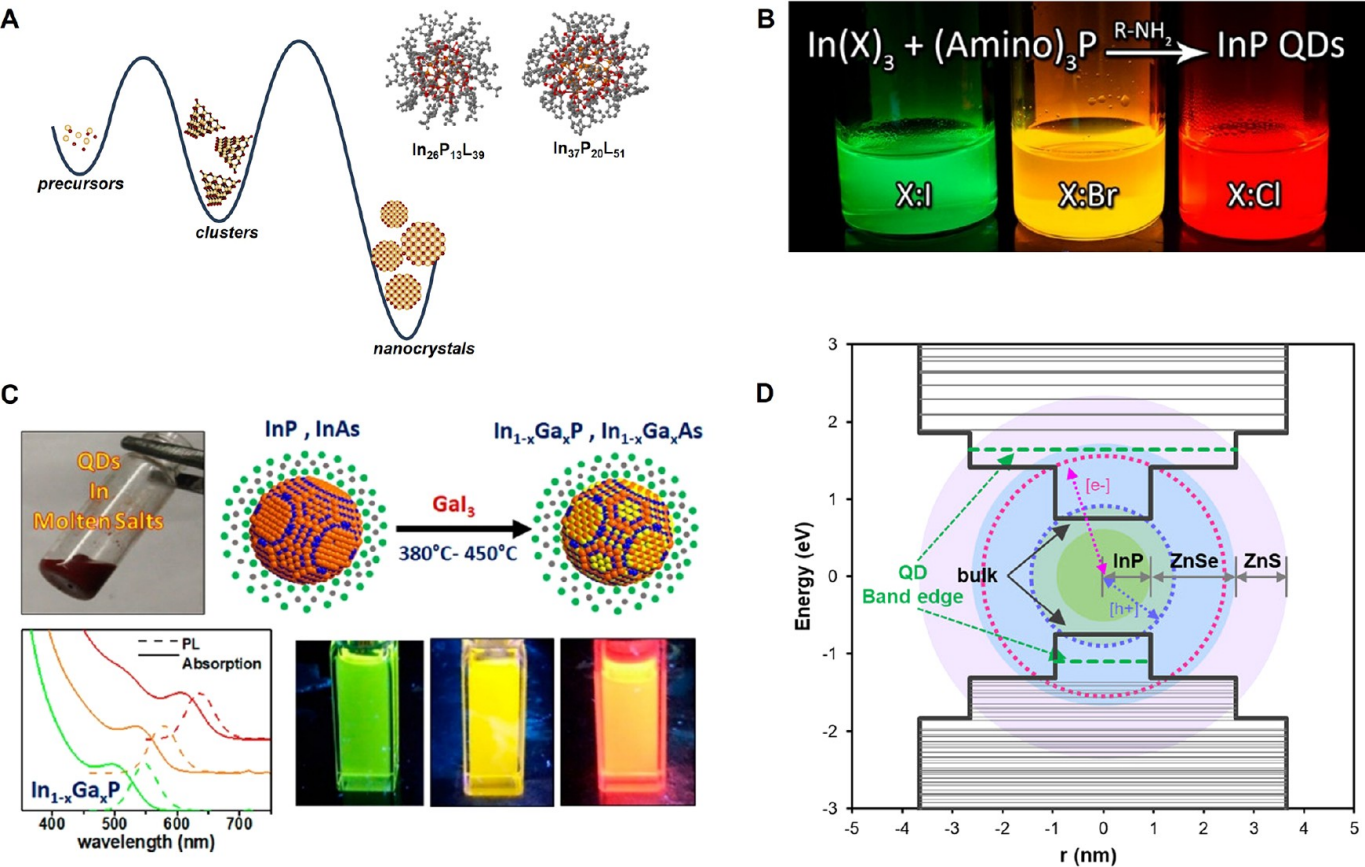
(A) Clusters have been implicated as critical intermediates along the potential energy landscape between molecular precursors and larger III–V NCs. Two of these clusters have been structurally characterized—In_26_P_13_(O_2_CR)_39_ and In_37_P_20_(O_2_CR)_51_. Adapted from refs 215,216 . Copyright © 2016 and 2024 American Chemical Society. (B) Aminopnictogen precursors (E­(NR_2_)_3_, E = P, As, Sb; R = Me, Et) have been introduced as versatile precursors in the synthesis of III–V NCs. Reproduced from ref 217. Copyright © 2015 American Chemical Society. (C) Molten inorganic salts have been introduced as high-temperature reaction media for the synthesis of highly crystalline colloidal III–V NCs. Reproduced from ref 218. Copyright © 2018 American Chemical Society. (D) Shell engineering has been a critical issue in obtaining InP QDs with high PL QY and good environmental stability. Reproduced from ref 219. Copyright © 2020 American Chemical Society.

Aminopnictogens (E­(NR_2_)_3_, E = P, As, Sb; R = Me, Et) have been identified as versatile precursors in the synthesis of metal pnictide NCs across a broad size range (Figure 7B). In 2013, Yang introduced the use of tris­(dimethylamino)­phosphine in a primary amine solvent as a lower toxicity, easier to handle reagent for the synthesis of InP QDs.^228^ In 2015, Hens’ group took this synthesis one step further and demonstrated that precursor-controlled size tuning could be achieved by tuning the identity of the InX_3_ (X = Cl, Br, I) reagent in an aminophosphine synthesis.^217^ Given that P is in the +3 oxidation state in P­(NR_2_)_3_ precursors, it was clear that the reaction mechanism in this chemistry must involve a redox step. Dubertret and Hens’s group separately revealed that the primary amine solvent is a critical component of the reaction, leading to transamination followed by the disporportionation of the phosphorus precursor.^18,229^ On the other hand, this reaction requires a high P:In ratio (at least 4:1) making it challenging to access larger QD sizes. Moreover, the same concept cannot be applied to the heavier pnictides, as aminoarsine and aminostibine precursors do not undergo the self-reduction via disproportionation. Changing the indium precursor chemistry by switching from In­(III) to In­(I) halides can overcome these shortcomings as demonstrated by Reiss for InP and InSb and Bawendi for InAs QDs.^230–232^ In this case, the In­(I) halide acts as both the indium precursor and reducing agent of the aminopnictogen precursor.

Alternatively, the addition of external reducing agents such as borohydrides has been explored to extend the use of aminopnictide precursors to several other material platforms, including InAs, InSb, and GaAs,^233–236^ as well as a variety of other main group and transition metal phosphides.^237^ Recently, Owen and co-workers demonstrated that InP nucleation in the aminophosphine system, and perhaps more generally, proceeds through a continuous mechanism with size-dependent growth kinetics responsible for the narrow size distributions.^43^ This has been exploited by Boyer and Cossairt to push the limits of size control in this system.^238,239^

The use of molten salts as a higher temperature medium for colloidal synthesis and postsynthesis modification of III–V materials has proven very effective (Figure 7C). Talapin’s group has shown that, especially for Ga-containing III–V NCs, access to higher temperatures between 380 and 500 °C is critical for obtaining high-quality colloidal NCs with low densities of internal defects. These temperatures are only accessible using nontraditional solvent media like molten salts.^218,240,241^ Moreover, it has been shown that controlling the molten salt environment can produce mechanisms for kinetic control, enhanced phase stability, and tunable emission line widths, affording access to a continuous range of In_1–*x*_Ga_*x*_P alloyed NCs—strategies that are expected to be generalizable to other III–V compositions.^241^ More details can be found in the following section.

Effective surface engineering and shelling methods have further pushed the boundaries of the synthesis of III–V materials (Figure 7D). Controlling the stoichiometry (In:P ratio) and oxidation level of InP cores is a critical first step in obtaining high-quality InP emitters for both downconversion and electroluminescence applications.^242,243^ Notably, the pretreatment of InP cores with HF, alternative fluoride sources, or via controlled oxidation prior to or during the first stages of shelling is beneficial for obtaining near-unity QYs.^219,244–252^ It is generally believed that HF and related species remove or passivate electronic traps, but revealing the exact underlying chemical mechanisms remains a challenge and an active area of investigation. Removal of oxidized phosphorus and trap passivation by surface fluorination have been suggested.^244,245,247,251^ A study using anhydrous HF has identified that a primary mechanism by which HF enhances PLQY is by breaking up and removing polyphosphates, providing a surface that is amenable to further passivation.^246^ The etching induced by HF generates InF_3_, which solubilizes at elevated temperature and serves as a ligand to likely passivate sterically congested surface dangling bonds. To avoid etching, alternative fluoride sources, such as InF_3_, have been directly applied, leading to near-unity PLQY without etching.^253^

The most successful approach to shelling uses a gradient or multishell approach, most commonly involving ZnSe/ZnS.^219,242,248,249,254–256^ Controlling the quality of the core, the relative thickness and alloy ratios of the ZnSe/ZnS layers, and the conformity and uniformity of the shell are essential for obtaining the best quality emitters with photoluminescent QYs of >85%. Given the challenges of avoiding oxidation of the InP core surface, alternative oxide interlayers have also been explored, with ZnO demonstrating improvements in terms of both PL and material stability.^257–259^

Owing to these developments, the future of III–V NC synthesis is bright. We are beginning to understand how to leverage atomically precise clusters, alternative precursor chemistries and reaction media, and intricate surface engineering to generate a broad array of III–V NCs with optimal form and function. And there are several exciting areas for further investigation. One is developing arsenide and nitride materials with strong absorption and efficient emission in the NIR, SWIR, and mid-IR regions. Toward this end, InAs has seen a surge of interest with notable advances in size control and PL.^226,260–263^ Additionally, shape control of III–V NCs remains an outstanding challenge. Differences in surface passivation requirements relative to II–VI NCs have been suggested as one critical barrier to preventing the growth of 2D structures.^264^ However, the long history of InP nanowire growth using solution-liquid–solid chemistry^265,266^ hints that there should be viable strategies for obtaining III–V semiconductors in the form of 1D and 2D nanostructures.

### Molten Salts

The nature of chemical bonding is highly relevant to the difficulty of materials synthesis. Empirically, we know that many “difficult-to-synthesize” NCs, like Si, GaAs, or diamond, have strong covalent chemical bonds. Successful growth of a defect-free crystal requires reversibility of bond formation to enable self-healing of structural defects. Materials with strong chemical bonds require high synthesis temperatures, often far above temperatures accessible for traditional solvents used in colloidal synthesis: even the most robust organic solvents are falling apart at temperatures above 400 °C. For example, the optimal temperature for chemical vapor deposition of GaAs is between 600 and 800 °C, whereas the materials grown below 500 °C are highly defective and not suitable for optoelectronic applications.^267^ Numerous attempts to synthesize GaAs NCs by traditional colloidal methods just kept burning graduate student time without making tangible outcomes.

To expand the scope of synthesizable colloidal nanomaterials, it has been recently proposed to use molten inorganic salts as solvents for transformations of colloidal NCs.^218,241,268,269^ Molten inorganic salts have successfully been used as inert or reactive fluxes for solid-state chemistry,^270^ and crystal growth.^271,272^ Their advantages include a broad range of accessible temperatures, wide windows of electrochemical and chemical stability (Figure 8B), and the ability to dissolve many solids that are insoluble in traditional solvents.^273^ Molten salt fluxes have been explored in the synthesis of oxides, metal alloys,^274^ and covalent compounds (SiC,^275^ Si,^276^ graphene,^277^ carbon nanotubes^278^). However, the utility of molten salts for colloidal chemistry has been recognized only recently, after the first observation of stable colloidal dispersions of NCs in these unusual solvents.^279^ The phenomenon of colloidal stability in molten salts cannot be explained by traditional electrostatic and steric stabilization mechanisms; these colloids most likely form through a mechanism based on the long-range ion correlations in the molten salt induced by the crystal surface.^279–281^

**Figure 8:**
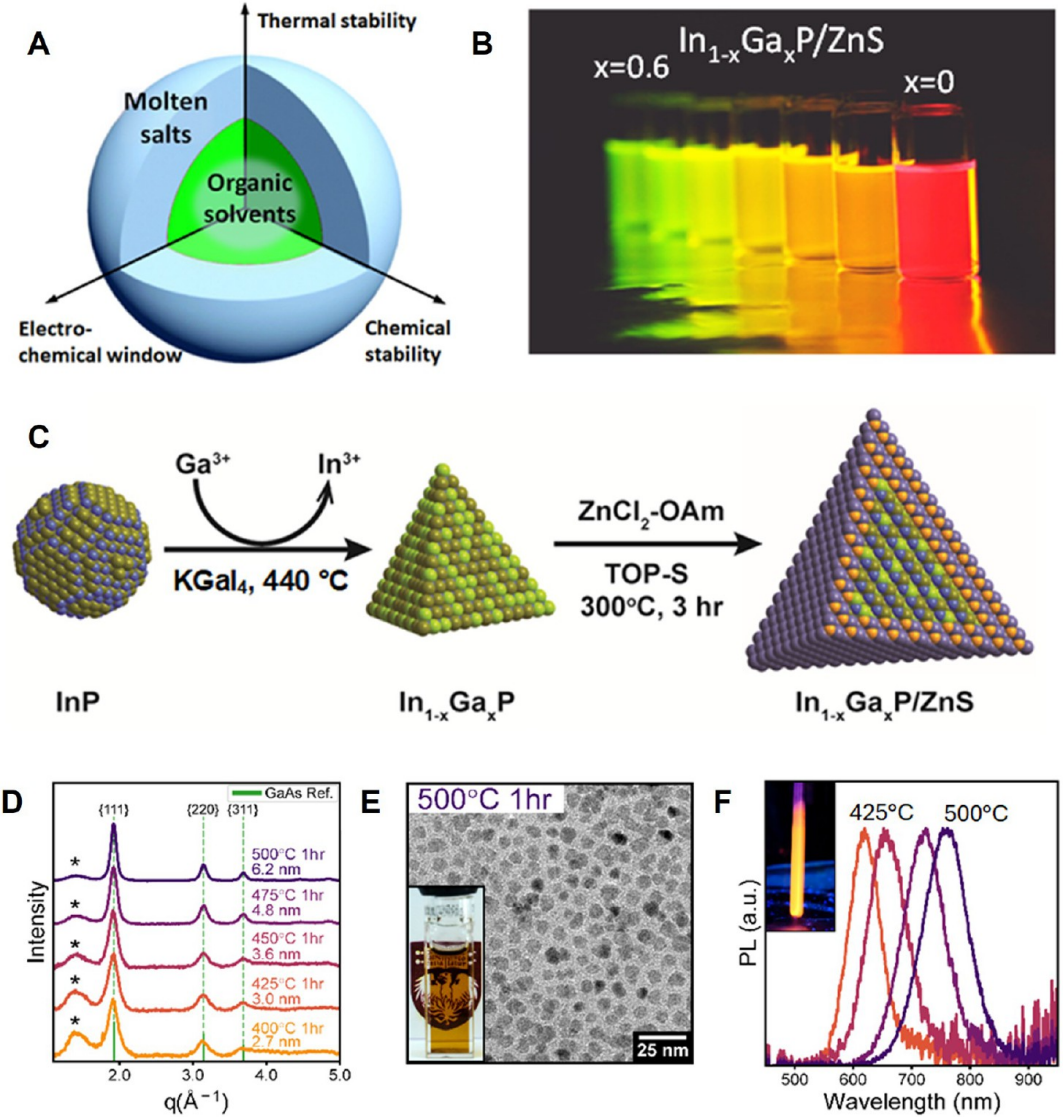
(A) Comparison of molten inorganic salts with organic solvents. (B) Photograph showing the range of emission colors produced by core–shell In_1–*x*_Ga_*x*_P/ZnS samples with varying gallium content derived from the same 4.0 nm InP NCs. (Red emission is from the InP/ZnS sample corresponding to *x* = 0). PL QY was 60–90% for all samples containing 0–50 mol % gallium. (C) Reaction scheme describing the conditions for the In-to-Ga cation exchange and the subsequent ZnS shelling steps. (D) Powder X-ray diffraction patterns of colloidal GaAs NCs directly synthesized in a molten salt at temperatures from 425 to 500 °C. The (*) peak originates from X-ray scattering from the organic ligand shell. (E) Transmission electron microscopy (TEM) image of colloidal GaAs NCs synthesized in a molten salt at 500 °C. The inset shows the same sample as colloidal solution in toluene. (F) Room-temperature PL from GaAs NCs synthesized at 425 to 500 °C, with inset photo of GaAs NCs synthesized at 425 °C under UV (ultraviolet) illumination. Panels (B) and (C) adapted from ref 268. Copyright © 2023 American Chemical Society. Panels (D), (E), and (F) reprinted with permission from ref 282. Copyright © 2024 The American Association for the Advancement of Science.

The discovery of colloidal solutions in molten salts offered an opportunity for the synthesis of novel NC materials. The first generation of synthetic methods used the conversion of indium pnictide (InP, InAs) NCs into ternary In_1–*x*_Ga_*x*_P and In_1–*x*_Ga_*x*_As QDs by cation exchange reactions.^218,240,241,268^ In these reactions, the InP and InAs NCs are first synthesized using traditional organic solvents, stripped of their native organic ligands, and transferred into molten salt reaction media, either with “bare” surfaces or with all-inorganic ligands such as III-halide salts or metal chalcogenides.^241,268^ Next, they are annealed at temperatures from 380 to 500 °C (Figure 8C). By carefully tuning the Lewis acidity and redox potential of the molten salt, it is possible to preserve the size and avoid aggregation or sintering of NCs, thus retaining their original size distribution. To isolate the annealed NCs, the reaction is cooled, and solidified salt matrix is dissolved in an appropriate solvent (formamide, acetonitrile, *etc*.) and the NCs, which are insoluble in the solvent, are recovered by centrifugation. Finally, an epitaxial wide-bandgap shell of ZnS or ZnSe is grown around the III–V cores after making the NCs colloidal in organic solvents by addition of appropriate surface ligands (*e.g*., oleylamine, oleic acid, *etc*.). Importantly, this approach results in highly luminescent In_1–*x*_Ga_*x*_P/ZnS QDs, with PL QYs in the range of 60–90% (Figure 8D). Molten salt solvents are thus far the only route that has resulted in highly emissive In_1–*x*_Ga_*x*_P and In_1–*x*_Ga_*x*_P QDs over a wide composition and size range, having bandgaps tunable throughout the visible and NIR.

Despite the apparent straightforwardness of the cation exchange process, there are significant knowledge gaps related to the mechanistic details of this process. For example, modeling the kinetics of the cation exchange by solving Fick’s diffusion equations using the diffusion coefficients reported for corresponding bulk III–V semiconductors^283^ reveals a discrepancy of many orders of magnitude between theoretical predictions and experimental data.^240^ The diffusion model predicts ∼30 years for half-complete exchange vs. less than 1 h experimentally, an obvious knowledge gap in our understanding of the cation exchange mechanism in III–V NCs.

The cation exchange reactions are just the first step in the exploration of molten inorganic salts as solvents for the synthesis of novel colloidal NCs. A logical next step is the direct synthesis of colloidal NCs in molten salts. As an intermediate step, a biphasic mixture of KGaCl_4_ molten salt and alkylamines was shown to produce crystalline GaN and AlN NCs.^269^ Finally, in 2024, it has been demonstrated that colloidal III–V NCs can be efficiently nucleated and grown directly in molten inorganic salts.^282^ For example, Ga_2_I_4_ dissolved in KGaI_4_ salt (melting point 230 °C) can simultaneously be a reducing agent for AsI_3_ and a gallium source to prepare GaAs NCs according to the reaction 3Ga_2_I_4_ + AsI_3_ → GaAs + 5GaI_3_. This reaction typically proceeds at 400 to 500 °C, synthesized GaAs NCs are recovered as a powder by dissolving salt matrix, and oleylamine/ZnCl_2_ ligands are installed on the NC surface to enable colloidal dispersion in toluene or other nonpolar solvents. Powder XRD patterns (Figure 8D) show ZB GaAs with increasing crystallite size for NCs synthesized at increasing reaction temperatures. TEM images of GaAs NCs synthesized at 500 °C (Figure 8E) show well-separated particles with a reasonably narrow size distribution (∼15%) that form a stable colloidal solution in toluene. The formation of NCs from molecular reagents shows that molten salts can balance NC nucleation and growth kinetics, similar to colloidal synthesis in conventional organic solvents. GaAs NCs synthesized between 425 and 500 °C show room temperature PL (Figure 8F), but samples synthesized at 400 °C or lower temperatures did not show any detectable PL at room or low temperature. As such, it appears high-temperature synthesis is required to produce emissive GaAs QDs.

There is every reason to believe that molten salts will further expand the scope of synthesizable NC materials. From the practical side, questions remain about whether molten salt colloidal synthesis can be scaled to produce gram-to-ton scale amounts of NCs. While not as widely appreciated, many critical industrial processes use molten salt solvents. For example, the production of aluminum using the Hall–Héroult process uses electrochemical reduction of alumina in molten Na_3_AlF_6_ solvent at ∼1000 °C. Given the production of aluminum occurs on a multimillion-ton-per-year scale, there are no intrinsic problems with scaling molten salt chemistry if needed.

### Metal Borides

Over the last three decades, the advantages of colloidal NCs have been explored for semiconductors, metals, and oxides, leaving the field with a conspicuous absence of boron­(B)-based colloidal nanomaterials. Bulk metal borides (Figure 9, M_*x*_B_*y*_) that are suitable for extreme conditions due to increased hardness and wear resistance remain insufficiently explored at the nanoscale despite their attractive properties. They traditionally require high-temperature, high-pressure synthesis processes and thin-film fabrications.^287,288^ Metal borides can be clasified as boron-rich (BR) metal borides: hexaborides (MB_6_, M = Ca, Sr, Ba, LA, Ce),^288–290^ diborides (MB_2_, M = Mg, Ti, Hf, Zr, Al, V),^291–294^ and metal-rich (MR) metal borides: M_3_B (M = Co, Ni, Pd).^295–298^ All of these are of significant interest due to their wide range of structural characteristics, properties, and potential applications, and they are tunable through the choice of metal and B substructures.^287,288^

**Figure 9:**
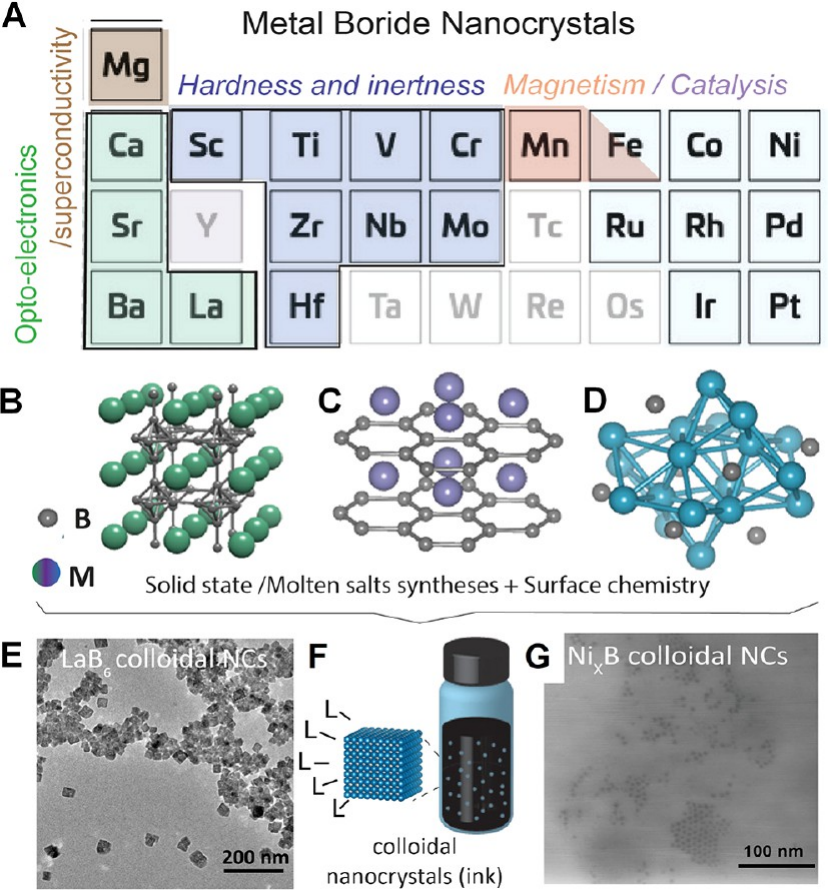
(A) Periodic table representation of the metal borides, their properties, and applications. (B–D) The most common crystal structures for metal borides: (B) MB_6_ (Pm3m), (C) MB_2_ (*I*4/*mcm*), and (D) M_3_B (*Pnma*). Reproduced from ref 284. Copyright © 2024 American Chemical Society. (E) Representative TEM image for colloidal LaB_6_ NCs. Reprinted with permission from ref 285. (Copyright © Protesescu et al.). (F) Cartoon representing the colloidal nature and surface chemistry for metal boride NCs. (G) Representative TEM image for colloidal Ni_3_B NCs. Reproduced from ref 286. Copyright © 2023 American Chemical Society.

Colloidal MB_6_ NCs, with attractive optoelectronic properties,^285^ could find utility in tandem solar cells and upconversion materials. Regarding the mechanical properties, hardness values of metal borides were only studied in bulk and vary from 10 to 35 GPa for MB_6_ and MB_2_.^299–303^ Very few reports about the hardness of microcrystalline films show the potential of TiB_2_ nanoparticles with hardness >40 GPa. In comparison, the stoichiometric bulk reaches only 25 GPa.^304^ This is due to the excess of B atoms on the surface, which are prone to creating B–B covalent substructures.

Typically, the synthesis of metal borides involves high-temperature methods, ranging from 1000 to 1600 °C, which have been widely explored. However, when it comes to nanosizing these materials, the available methods have certain limitations due to the unique nature of B–B bonding (B–B_interoct_, B–B_intraoct_).^305^ The exploration of metal borides NCs is still in its infancy, with solid-state and molten salt syntheses emerging as promising approaches.^305^ Those methods include the use of borohydrides (highly reactive), B halides, B oxide, or amorphous B (often requiring additional substances like sodium or magnesium).^305,306^

Solid-state synthesis has successfully produced phase-pure nanocrystalline MR-M_*x*_B_*y*_ and BR-M_*x*_B_*y*_ at relatively low temperatures.^44,284^ This method’s simplicity makes it appealing for large-scale production, although understanding reaction mechanisms is vital for controlling size and properties. In a typical synthesis, the metal halides are preball milled with NaBH_4_ (used as the B source and reducing agent when needed) and then mixed at temperatures below 500 °C without the addition of any solvent for 3 to 48 h to obtain metal boride NCs in a powdered form. After the purification steps, the surface of the NCs will be functionalized to achieve stable colloidal suspensions in polar or nonpolar solvents, as dictated by the ligands on the surface (Figure 9E–G).^284–286^

While offering some control over size and morphology, molten salt synthesis faces challenges in achieving phase purity, especially for MR-M_*x*_B_*y*_, and requires high temperatures (700–900 °C).^305,307^ This method is efficient when the B source (elemental B, NaBH_4_, or B_2_O_3_) is reacted with a metal halide (usually chloride) in a eutectic salt mixture such as LiCl/KCl or Na_2_B_4_O_7_ /KCl. Several reports have demonstrated that this method produced small crystalline nanosized particles (<10 nm); however, it still required high temperature (700–900 °C).^305,306,308,309^

Both methods have challenges in controlling size, morphology, and phase purity. The washing process introduces an amorphous oxide layer on metal boride NCs, affecting their properties.^285,286^ Alternative purification methods or postsynthetic processes are necessary to remove this layer for optimal performance. Surface studies suggest the increased potential for mesoscopic multicomponents ink preparation (NCs and additives) but also indicate a common core–shell structure in metal boride NCs, influencing their properties and solution processability.^285,286^ For instance, Hong et al.^286^ demonstrated that the surface of Ni_3_B NCs is dominated by Ni^2+^ species (oxide and hydroxides), which facilitate the surface functionalization with anions such as tetrafluoroborate or amines (common ligands for such sites), leading to stable colloidal suspensions and enabling the deposition of these inks via solution processing (Figure 9G).^286^

While the potential of colloidal metal boride nanomaterials for a variety of applications has been recognized, significant challenges remain in fully unlocking their capabilities at the nanoscale. Despite their promising properties—such as high hardness, wear resistance, and optoelectronic performance—methods to synthesize and control the size, morphology, and phase purity of metal boride NCs are still under development. The high-temperature, high-pressure synthesis methods traditionally used for bulk metal borides are not easily adaptable to the nanoscale, with solid-state and molten salt methods offering some promise but still facing limitations in terms of scalability and controlling the physical characteristics of the NCs.

Moving forward, there is a clear need for more efficient synthesis techniques that can overcome these issues, as well as further investigation into alternative postsynthetic and functionalization processes. The development of strategies for stable colloidal suspensions and better control over size and surface characteristics will be crucial for advancing the application of M_*x*_B_*y*_ NCs. These improvements will be particularly important in fields like quantum computing, catalysis, upconversion materials, and wear-resistant coatings, especially in harsh conditions where current materials underperform. Additionally, addressing the underlying reaction mechanisms in synthesis could pave the way for more precise tailoring of these materials’ properties, ultimately accelerating their integration into real-world technologies.

### Lead-Halide Perovskites

The past decade of nanoscience with NCs has been a testimony to enhancing the material scope of colloidal NCs. Parallel to the development of increasingly covalent and structurally hard NCs (*vide supra* for recent inroads into the challenging synthesis of III–V NCs, NCs in molten salts, and metal boride NCs), lead-halide perovskite NCs represent an antipode at the opposite end of the semiconductor material spectrum: an unusually ionic and structurally soft NC, with the compositional formula APbX_3_, where A is a monovalent cation and X a halide.

Kickstarted by the first hot-injection synthesis of colloidal all-inorganic CsPbX_3_ (X = Cl; Br; I) NCs in 2015,^310^ and preceded by a nontemplated synthesis of CH_3_NH_3_PbBr_3_ colloids in 2014,^311^ such lead-halide perovskite NCs have rapidly and profoundly changed the research landscape of colloidal NCs. Less than a decade after their discovery, these rather ionic and structurally soft NCs already represent a commercial product, available, *e*.*g*., from Sigma-Aldrich and Avantama, and are about to enter the market for down-converting display applications,^312,313^ as pixelated emitter structures^314^ and/or as composite emitter films in liquid-crystal displays.^315^ The embarrassingly facile synthetic access to semiconductor nanostructures of high optoelectronic quality may soon also proliferate additional classical optoelectronic devices spanning from lasers^316^ to LEDs,^317–319^ photodetectors,^320^ scintillators,^321,322^ security tags,^323,324^ luminescent concentrators,^325^ and solar cells.^326^ Importantly, lead-halide perovskite NCs are increasingly being recognized also beyond the colloidal-NC community, in emerging fields such as photonic quantum technology, while triggering curiosities also in presently more explorative schemes, such as neuromorphic computing.^327^

The attractivity of halide perovskite NCs can be attributed to a compelling set of features: first, their facile low-temperature synthesis, enabled by comparably weak chemical bonds, renders the NC preparation accessible to many laboratories around the world and amenable to economic scale-up on an industrial scale. Second, the composition is easily tuned at both the A-site and X-site via facile and rapid ion-exchange reactions. Third, this material class offers superior optical properties, *e*.*g*., near-unity PL QYs^328^ narrow-band emission,^329^ tunable across the visible spectrum^310,330^ (Figure 10A), and rapid radiative decay (few nanoseconds at room temperature^310,331^ and down to sub-100 ps at 4 K).^332,333^ Especially the rapid radiative decay, in conjunction with near-unity PL QYs, renders perovskite NCs so unusually bright: perovskite NCs represent one of the fastest cavity-free emitter platforms ever reported. Such rapid emission has been rationalized in terms of their bright triplet fine structure^334^ and single-photon superradiance at cryogenic temperature.^333^

**Figure 10:**
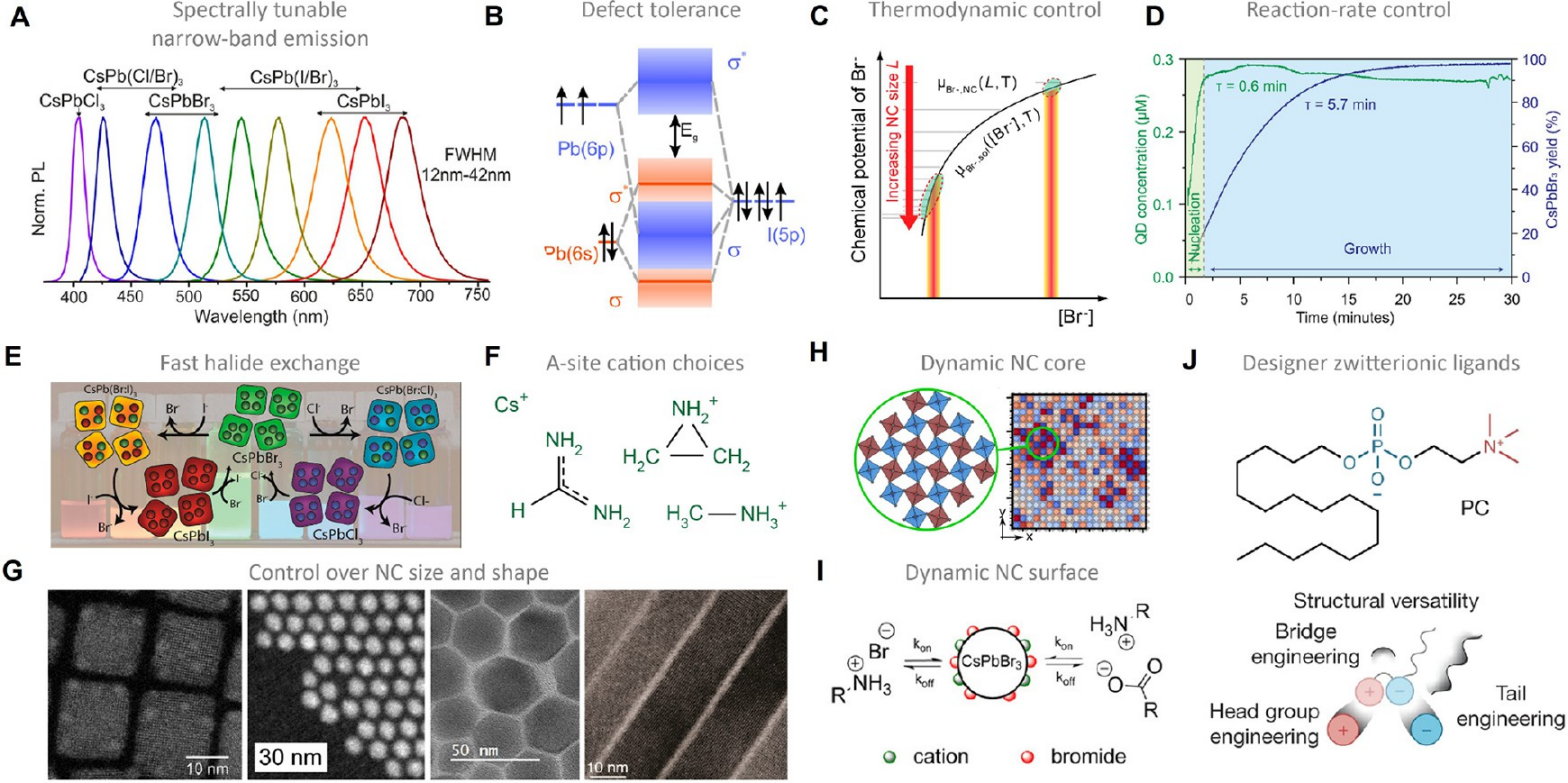
Lead-halide perovskite NCs. (A) Narrow-band PL is achieved across the entire visible range, with spectral tunability primarily via compositional control, and typically finer adjustment via size and shape control. Reproduced from ref 310. Copyright © 2015 American Chemical Society. (B) The high electronic quality is attributed to defect tolerance emerging from the Pb-halide bond. Reproduced from ref 342. Copyright © 2016 American Chemical Society. (C, D) Various synthesis routes exist, including hot-injection and ligand-assisted reprecipitation, and the synthesis can be fine-tuned via, *e.g*., (C) thermodynamic or (D) kinetic control. (C) is reproduced from ref 344. Copyright © 2018 American Chemical Society. (D) is reprinted with permission from ref 3. Copyright © 2022 The American Association for the Advancement of Science. (E–G) The parent crystal structure offers a multitude of NC core engineering possibilities, with compositional tuning through (E) fast X-site anion exchange reactions and (F) the choice of the A-site cation, next to (G) size and shape control. (E) is reproduced from ref 330. Copyright © 2015 American Chemical Society. (G) is reproduced from refs 3,410–412 . Copyright © 2022 The American Association for the Advancement of Science. Copyright © 2016, 2020, and 2024 American Chemical Society. (H–J) Compared to more covalent NCs, the rather soft and ionic perovskite crystal structure results in (H) pronounced structural dynamics in the NC core and (I) altered surface chemistry, characterized by dynamic ligand binding, calling for (J) ligands expressly designed for perovskite NC surfaces. (H) is reprinted with permission from ref 395. Copyright © 2023 Elsevier Inc. (I) is reproduced from ref 346. Copyright © 2016 American Chemical Society. (J) is reprinted with permission under CC BY 4.0 from ref 393. (Copyright © 2023, Morad et al., open access).

Notably, the outstanding optical properties of lead-halide perovskite NCs have been achieved in the complete absence of an electronically passivating epitaxial inorganic shell, and with a structurally soft NC material exhibiting large ion mobility. While the former already presents a major advance compared to previously studied II–VI, IV–VI, or III–V NCs, which typically require laborious and sophisticated core/shell synthesis routes to achieve high PL QYs, the latter seemingly contrasts previous design principles primarily aimed at avoiding structural defects in the NCs and their surfaces.^335,336^

The conundrum was resolved by realizing the peculiar electronic defect tolerance of lead-halide perovskites (Figure 10A):^337^ thanks to the antibonding character of the valence-band edge and the close energetic proximity of Pb­(6p) atomic orbitals to the conduction-band edge, the material sustains a nearly defect-free *electronic* structure even for comparably defect-rich *physical* structures of the bulk material and the NC surface.^328,338–342^ However, the electronic defect tolerance has limits and still requires a sufficient degree of passivation via ligands^328^ and/or suitable electrostatic engineering^341^ at the NC surface, motivating further surface-chemistry efforts (*vide infra*).

Among the synthetic approaches yielding isolable and processable colloidal lead-halide perovskite NCs, the hot-injection synthesis route in apolar solvents^310^ and the ligand-assisted reprecipitation route in mixed solvents^313^ are the most commonly employed to date. Especially the former excels in yielding superior size homogeneity (typically <10%), size control, and PL QY (typically 50–90%). Compared to II–VI, IV–VI, and III–V NCs, lead-halide perovskite NCs can be synthesized at much lower (even room) temperatures,^3^ and the synthesis proceeds more rapidly, typically within (sub)­seconds, yielding NCs of unusual brightness, even in the absence of an epitaxial inorganic shell.^310,343,344^

Since 2015, a surge in synthetic developments has sought to deepen the understanding of the lead-halide perovskite NC formation and growth mechanism (Figure 10B),^3,37,344^ quantify the reaction yield and stoichiometry,^3,345^ further enhance the size and shape uniformity (Figure 10C),^3,344^ elucidate ligand binding and exchange (Figure 10D),^346,347^ investigate the surface structure,^328,338^ or explore alternative paths to the hot-injection synthesis route.^313,348^ An initial target has been to devise alternatives to the original two-precursor scheme

$$2\;\mathrm{Cs}\left(\mathrm{oleate}\right)+3\;\mathrm{Pb}{\mathrm{Br}}_{2}\overset{\;}{\to }2\;\mathrm{CsPb}{\mathrm{Br}}_{3}+\mathrm{Pb}{\left(\mathrm{oleate}\right)}_{2}$$

to overcome its incomplete stoichiometry control and intrinsically limited reaction yield, with lead oleate and related complexes as significant side products.^346^ Departing from two precursors to three precursors,^317,345^ one each for the A, Pb, and X ion, achieved higher control over the final A:Pb:X stoichiometry, suppression of halide vacancies, and near-unity reaction yields.

As for other NC platforms, control over composition, size, and shape constitutes an important goal. Compositional control capitalizes on the vast chemical space offered by the multielement ABX_3_ parent composition and its solid solutions, as well as the high ion mobility (Figure 10C). Structural, electronic, and thermal tunability were achieved via the choice of the **A-site** cation, thus far using Cs, FA (formamidinium), MA (methylammonium), and/or AZ (aziridinium),^349^ as well as via the various (**X-site**) halide anions (Cl, Br, and/or I) incorporated either through altered initial precursors or postsynthetically via fast anion-exchange reactions.^326,330,350^ While the X-site anion has provided the widest bandgap tunability, spanning the entire visible spectrum, the A-site cation increasingly emerges as a powerful additional handle to fine-tune the static and dynamic structure,^351,352^ charge transport,^353^ bandgap broadening,^354^ emission lifetime,^323^ and single-photon purity.^355^ For **B-site** compositional tuning, we refer the reader to here below, where tin-halide perovskite NCs^356–362^ are discussed as the most notable lead-free metal-halide perovskite alternative^363–372^ studied to date. Control over NC size and shape is reaching a mature stage (Figure 10C). Initial efforts targeted an increase of the nanocube fraction at the expense of competing shapes such as NPLs or nanosheets, through alkylamine-free synthesis schemes employing secondary amines,^373^ quaternary ammonium salts,^317^ or TOPO.^374^ More recent developments explored the synthesis of anisotropic NCs such as NPLs^375–379^ and NRs/-wires,^380,381^ exploiting control via ligands, temperature, concentration, and/or thermodynamic vs kinetic regimes.^344^

Enabled by the large and growing community working on colloidal perovskite NCs, continued synthetic advances are being made at a rapid pace and on various fronts. Selected notable examples include: (i) thermodynamically (instead of kinetically) controlled reactions;^344^ (ii) droplet-based microfluidic platforms for fast parametric screening;^382^ (ii) templated synthesis schemes in confined spaces, *e*.*g*., in mesoporous Si,^383^ and (iv) a slow room-temperature reaction^3^ (proceeding within up to 30 min instead of typically within less than a second) utilizing weakly binding acid and TOPO. The latter route yields spheroidal NCs of high size uniformity, well-separated nucleation and growth stages, and, unlike previously explored synthesis routes, decouples the ligand choice for the final product (here attachable *post*-synthesis) from the ligand choice for the nucleation and growth stage.

Unsurprisingly, the fundamentally different core of perovskite NCs also translates into fundamentally different NC surface chemistry (Figure 10D). For example, the well-known oleic acid and oleylamine ligands, commonly employed for the more established, *e*.*g*., III–V, II–VI, or IV–VI semiconductor NCs, bind only weakly to perovskite NC surfaces (Figure 10D), with an unusually rapid exchange between a surface-bound and free ligand state,^346,347^ compromising the NCs’ optical performance and stability.^384^ Sensitive acid–base equilibria^385^ and the propensity of proton transfer from surface-bound oleylamine (in its protonated form as oleylammonium) to the surface-bound oleic acid (in its deprotonated form, as oleate),^346^ further compromise the stability and render this originally, and still widely used, ligand pair a suboptimal choice for perovskite NCs.

The task is clear: the community needs to find better binding ligands for perovskite NCs, ideally by rational design and expressly catering to their unusually ionic NC core. One contemporary design strategy is to replace the most covalently bound ligand headgroup,^384^ *i.e*., oleate binding to lead, by softer alternatives, such as softer carboxylates, phosphonates, or other X-type ligands. Furthermore, the search for better binding ligands clearly revealed the need for properly acknowledging the specifics of perovskite NC surfaces, with factors such as binding affinity, steric hindrance at the binding site, entropic contributions, and interactions between solvent and ligand headgroup. At present, promising ligand examples include didodecylammonium bromide^328^ and various other quaternary ammonium,^386,387^ phosphonate,^388^ diphosphine-based bidentate,^389^ and zwitterionic ligands,^390^ including sulfobetaines,^391^ natural lecithin,^392^ and a library of recently reported designer phospholipids.^393^ Especially the latter confer improved long-term colloidal stability, environmental stability (against humidity, heat, and irradiation), and stability against dilution (Figure 10D).^393^ A practical advantage of the phospholipid ligand platform is its modularity, which entails design freedom not only for the ligand binding groups and ligand (zwitterionic) bridge, but also for the ligand tail, yielding greatly enhanced solvent compatibility, an important asset for NC integration into established large-scale industrial processing chains. Being agnostic to the considered NC surface, ligand tail engineering naturally also represents a connection point between the various NC fields discussed in this perspective, inviting accelerated discovery via cross-fertilization efforts.

How can one further develop the optical performance and how the stability of perovskite NCs? Addressing both questions, and especially the former, may require developing a better understanding of the underlying crystal structure. For example, the structural softness of the APbX_3_ parent materials makes perovskite NCs not only nanostructures with “soft surfaces” (as for lead chalcogenide NCs)^394^ but nanostructures that are soft throughout, including in their core region. Combined with a pronounced anharmonicity of vibrational modes at room temperature and shallow energy landscapes, this translates into a propensity toward unusually large dynamic disorder at room temperature. Therefore, the generally handled crystallographic prescriptions (*e.g*., CsPbBr_3_ being orthorhombic, and MAPbBr_3_, FAPbBr_3_, and AZPbBr_3_ being cubic at room temperature) are to be interpreted as temporal and spatial averages only, with significant fluctuations in time and space.^352,366,395–397^ As a direct consequence, the electronic wave functions partially localize, which, at elevated temperatures, partially slows down the rapid radiative decay.^333,398^ Remarkably, for some compositions (CsPbBr_3_ and MAPbBr_3_ NCs) but not for others (*e.g*., FAPbBr_3_ NCs),^352^ the disorder is mostly dynamic in nature and can be frozen out by cooling to liquid-helium temperatures. Below about 10 K, both CsPbX_3_ and MAPbX_3_ NCs display high structural order, with well-defined vibrational spectra,^352,397^ and excel electronically, with rapid^333^ and spectrally narrow emission (down to <20 μeV for individual CsPbI_3_ NCs at 3 K).^399^ Clearly, continued efforts toward understanding and controlling the dynamic perovskite crystal structure as well as the NC core/ligand interface (and, if developed eventually, a core/shell interface) can light the way to advances in the optical performance of these emitters.

A pressing challenge remains the stability of lead-halide perovskite NC in polar environments, which is of major relevance for long-term storage in humid air but also in the context of solvent compatibility (involving polar solvents) during industrial processing. In this respect, the known motto “easy to make, easy to break” alluding to the low formation energies and ionic bonding of the lead-halide framework appears as a hard nut to crack for synthetic chemists.

In principle, realizing core/shell NCs with epitaxial core/shell interfaces could offer a remedy, as routinely done for, *e.g*., III–V, II–VI, or IV–VI NCs. The soft halide perovskite lattice may, at first sight, even be advantageous as it relaxes the typically stringent requirements for interfacial lattice match. In practice, however, the realization of core/shell structures is nontrivial, related to the highly dynamic structure with pronounced ion migration, preventing atomically sharp interfaces, and limited chemical stability, inducing decomposition potentially before achieving shell growth.^400^ Nevertheless, few promising steps toward perovskite core/shell NCs have been taken with more inert and soft shells, such as those grown via colloidal atomic-layer deposition^401–403^ or thin metal-oxide gel coatings obtained via a nonhydrolytic sol–gel approach,^404^ apart from following more traditional shell deposition approaches.^405^

Another point of attention is the facile reduction of Pb, requiring special care in electron microscopy,^406,407^ and complicating, in some cases even limiting, applications involving charge transfer, *e.g*., in photocatalysis, photodetection/-imaging or solar cells. Fundamental studies, such as employing electrochemistry^408,409^ and accompanying structural and electronic characterization, may help probe the stability limits.

In conclusion, lead-halide perovskite NCs continue to fascinate researchers and, within merely a decade, have established themselves as an essential member of the NC family. They equally serve as a fundamental-science playground (how can a semiconductor display textbook-like optical properties at cryogenic temperature but highly entropic behavior at room temperature?), constitute a serious challenge for the synthetic chemist (how can one develop suitable NC core and surface chemistries for such ionic and structurally soft compounds?), and constitute building blocks for a growing number of classical and quantum devices (how can one fully leverage their scalabilty and tunability while also meeting the stringent stability requirements in industrial processing and device longevity?). Clearly, their second decade is off to a flying start and time will tell which application fields, from catalysis to quantum technology, will profit next from their unusual NC properties.

### Tin-Halide Perovskites

Accompanying the rapid advances in synthesis, characterization, and device applications of lead-halide perovskites, the toxicity of lead has driven efforts to replace lead with tin. However, challenges arise from the unstable nature of the Sn^2+^ oxidation state and the limited understanding of associated chemical processes during synthesis. Addressing this gap, recent research has demonstrated an optimized synthetic route to obtain stable, tunable, and monodisperse ASnX_3_ (A = Cs, FA, X = I, Br) NCs, with size- and composition-tunable optical properties.^356–361,404^ The synthesis method involves combining a precursor mixture of SnX_2_, standard ligands (oleylamine and oleic acid) with a Cs-oleate precursor, resulting in cuboidal NCs with 6 to 10 nm lateral size in the cubic (*Pm*3*m*, CsSnBr_3_, Figure 11A,B) and γ-orthorhombic (*Pnma*, CsSnI_3_, Figure 11C,D)^360^ phases, which exhibit notable colloidal stability. The key to achieving this stability is using excess precursor SnX_2_ and substoichiometric Sn:ligand ratios. Structural, compositional, and optical investigations, coupled with density functional theory (DFT) calculations, elucidate the mechanism of NC nucleation and growth, revealing the formation of (R-NH_3_^+^)_2_SnX_4_, a 2D Ruddlesden–Popper (RP) nanosheet (Figure 11E–G), as a competitive intermediate and stable product. When CsSnX_3_ NCs are assembled in thin films, if residual RP nanostructures are present, a preferential positioning of those 2D nanosheets at the substrate interface below the CsSnX_3_ NCs demonstrates the capabilities of self-assembly to form long-range ordered superstructures.^360,362^

**Figure 11:**
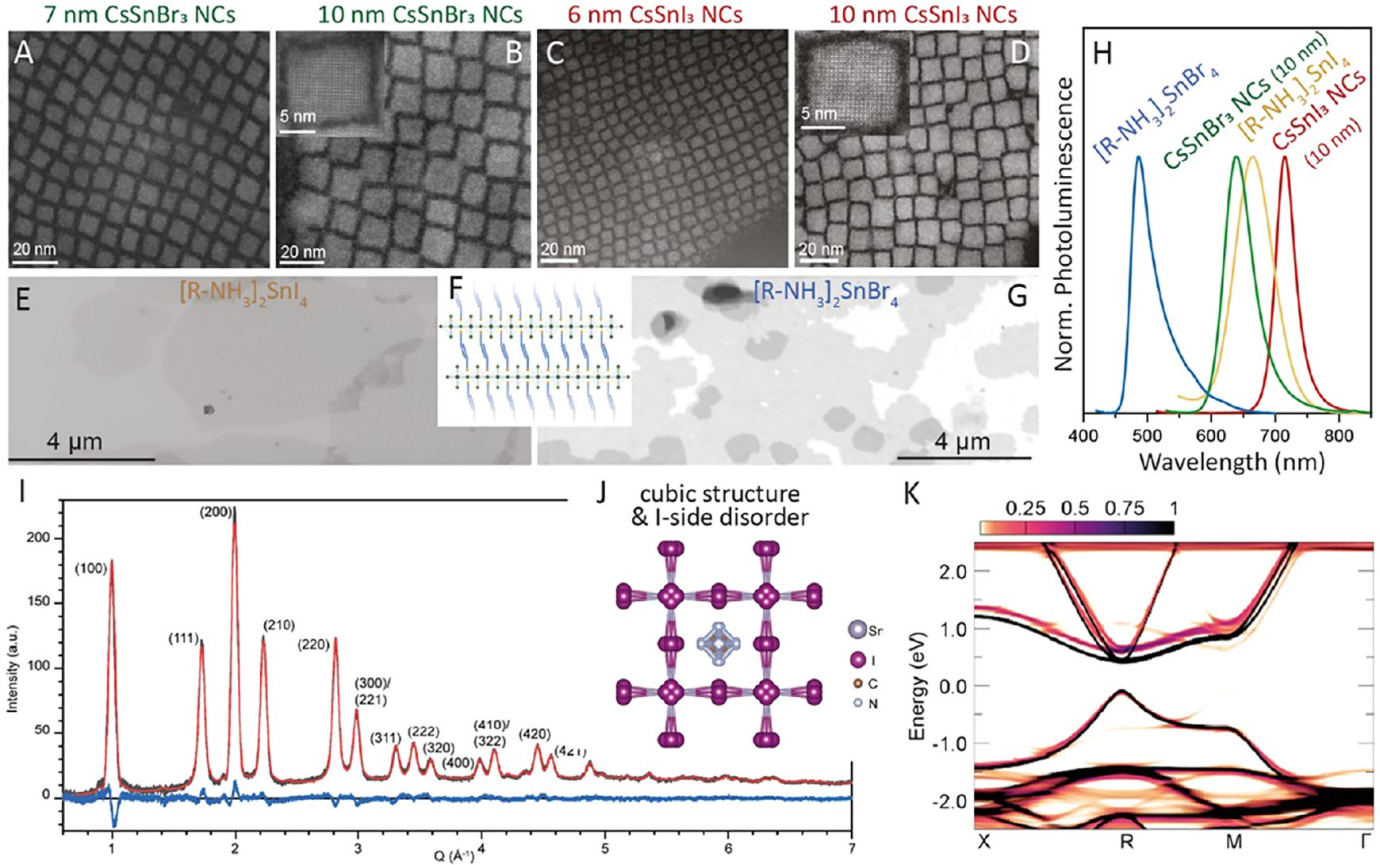
Tin-halide perovskite NCs. (A, B) TEM images for 7 and 10 nm CsSnBr_3_ NCs, respectively; the inset in (B) represents a high-resolution TEM image of a single CsSnBr_3_ NC. (C, D) TEM images for 6 and 10 nm CsSnI_3_ NCs, respectively; the inset in (D) represents a high-resolution TEM image of a single CsSnI_3_ NC. (E) Scanning electron microscope (SEM) image of (R-NH_3_^+^)_2_SnBr_4_ nanosheets. (F) Cartoon representation for the (R-NH_3_^+^)_2_SnBr_4_ crystal arrangement. (G) SEM image of (R-NH_3_^+^)_2_SnI_4_ nanosheets. (H) PL spectra of (R-NH_3_^+^)_2_SnBr_4_, 10 nm CsSnBr_3_ NCs, (R-NH_3_^+^)_2_SnI_4_, and 10 nm CsSnI_3_ NCs, respectively; R = oleyl. (I) Synchrotron wide-angle X-ray total scattering data of a colloidal solution of FASnI_3_ NCs (black), DSE simulation (red trace), and residual (blue curve). (J) Employed disordered cubic model in (I) (*Pm*3̅*m* space group), accounting for iodide disorder. (K) Momentum-resolved electron spectral function of FASnI_3_ calculated using the disordered structure in a 2 × 2 × 2 supercell and the band structure unfolding technique. Panels (A), (B), and (H) were adapted with permission under CC BY 3.0 from ref 362. (Copyright © 2024 Royal Society of Chemistry, open access). (C), (D), and (H) were adapted with permission under CC BY 4.0 from ref 360. (Copyright © 2022 Gahlot et al., Advanced Materials published by Wiley-VCH GmbH, open access). (E), (F), (G), and (H) were adapted with permission CC BY 3.0 from ref 361. (Copyright © 2024 Royal Society of Chemistry, open access). (I–K) were adapted from ref 414. Copyright © 2023 American Chemical Society.

Ion-exchange processes have been demonstrated following paths as the lead analogues in engineering the composition of Sn-halide perovskite nanostructures. A straightforward cation exchange process was achieved, where 2D RP nanostructures transitioned to 3D NCs by adding A-cation oleate to, for example, obtain ASnI_3_ (A = Cs, FA) from [R-NH_3_]_2_SnI_4_ in suspension or at the solid-liquid interphase of thin films.^361^ Anion-exchange processes between iodide and bromide counterparts further showcase the ability to modulate optical properties (Figure 11H).^361^

Fully rationalizing their optical properties at room temperature will require deeper studies into structural dynamics on the time scale of phonons. In this context, a recent study of FASnI_3_ NCs pointed toward a particularly pronounced halide disorder within the on-average cubic structure (Figure 11I,J). Translating into disorder also in their electronic structure (Figure 11K), such structural dynamics likely explain their rather weakly pronounced UV–vis absorption features at room temperature. In concert with the growing literature body on tin-halide perovskite NC syntheses, this structural-dynamics example highlights the need for additional studies targeting the fundamental understanding of their structural and optical characteristics.

On a more general note, ASnX_3_ NCs, as the most studied lead-free perovskite NC analogue to date, represent an important platform for evaluating the extent to which lead replacement can preserve, or in some cases even augment, the attractive features already found in APbX_3_ NCs. While the chemical stability of ASnX_3_ NCs could be addressed via encapsulation methods,^413^ further research is required to gain a deeper understanding of their optoelectronic properties and self-doping phenomena to advance to device-level materials. Moreover, tin halide perovskite nanostructures provide an excellent platform for gaining a fundamental understanding to develop promising metal halide/chalcogenide perovskite NCs.

## Surface Chemistry

The impact of surfaces on the chemical and physical properties of NCs is hard to overstate. NCs a few nanometers in size have, by-and-large, a similar number of bulk and surface atoms, and analyzing, understanding and tuning the termination of NC surfaces by adsorbents or ligands have become an integral part of NC science.^415^ In particular, the chemical schemes introduced about 10 years ago to classify the ligand/NC interaction,^416^ have led to highly effective ligand-exchange methods—even for emerging NC materials—and deeper insight into the relation between the surface structure and the NC properties. In parallel with this progress came the realization that NC surfaces are intrinsically heterogeneous, where facets, edges, and corners offer a variety of binding sites for which the weakest link rather than the average ligand can determine the NC properties.

### Ligand-NC Interactions

Following the work of Anderson et al.,^424^ it has become common practice to classify the interaction between surface ligands and surface atoms using the L, X, Z scheme introduced by Green to describe ligand coordination to metal centers.^425^ These three classes differ by the number of electrons contributed by the ligand to form a two-electron bond with a surface atom. In their neutral form, L-type ligands are Lewis bases that contribute two electrons, X-type ligand radicals that contribute one electron, and Z-type ligands Lewis acids that contribute no electrons.^426^ Initial studies on CdE (E = S, Se, Te) and PbE NCs mostly indicated surface passivation by X-type ligands, such as carboxylates or halides, for which the negative charge on the ligand compensates for the positive charge on excess cations.^417,418,426^

The chemical formula ME­(MX*_n_*), where ME represents the stoichiometric NC core and MX*_n_*, a surface moiety consisting of excess cations M^n+^ and X^–^ ligands, succinctly represents this NC class. However, as outlined in Scheme 1, a much wider variety of binding motifs emerged from more recent work on, for example, metal oxides,^420,427^ metal halides,^328,346^ or metal pnictides,^422,423,428^ which can be terminated by pairs of ions, or a combination of L-type and X-type ligands. Building on the classification scheme, such NCs can be represented as ME­(X)_2_ or ME­(MX*_n_*)­(L), respectively.

**Scheme 1:**
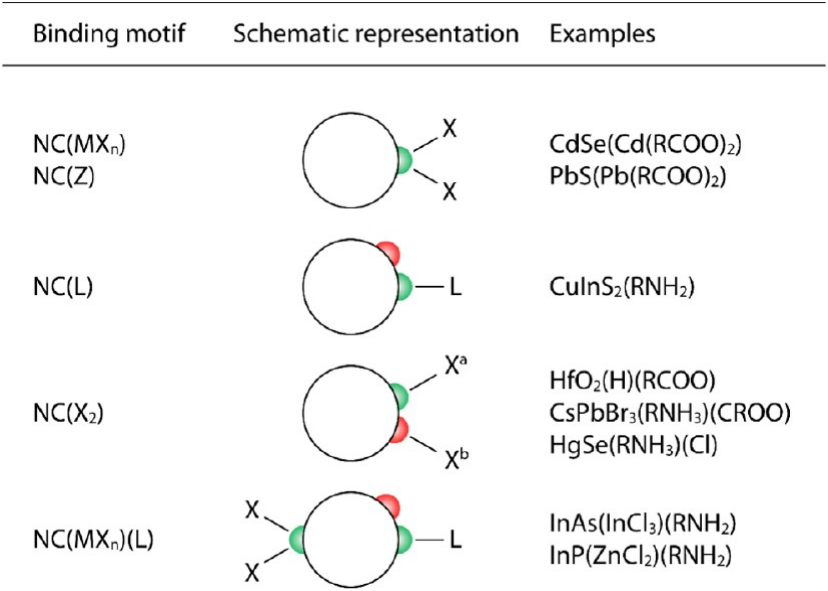
Representation of Different Binding Motifs, and Listing of Exemplary NC/Ligand Combinations^a^

a References from top to bottom: CdSe,^417^ PbS,^418^ CuInS_2_,^419^ HfO_2_,^420^ CsPbBr_3_,^346^ HgSe,^421^ InAs,^422^ InP.^423^.

A major benefit of classifying ligands was the insight that the salt MX*_n_* formed by the combination of one or more X-type ligands and an excess metal cation could be seen itself as a surface moiety within the same classification scheme.^416^ Here, metal carboxylates or metal halides, such as Cd­(Ac)_2_ or ZnCl_2_, are a case in point, where Ac^–^ or Cl^–^ can be interpreted as X-type ligands, or the entire salt as a Z-type ligand given the Lewis-acid character of the Cd^2+^ and Zn^2+^ metal center. What supported this conclusion was the observation that exposure of ME­(MX*_n_*) to L-type ligands led to the displacement of the salt MX*_n_* from the NC surface through coordination by the L-type ligands. As shown in Figure 12A, this process can be readily monitored through solution ^1^H NMR. Whereas purified, oleate-capped CdSe NCs exhibit a set of broad resonances that are characteristic of the oleyl chain of NC-bound oleate,^417^ addition of butylamine (BuNH_2_) results in the appearance of accompanying narrow resonances that can be assigned to cadmium oleate displaced from the NCs. Interestingly, by quantification of the NMR resonances of bound and displaced ligands, the surface coverage of oleate can be determined as a function of the BuNH_2_ concentration. As shown in Figure 12B, the oleate coverage drops quickly upon addition of small amounts of BuNH_2_, yet a residual oleate fraction of 20–25% remains bound, even at the highest BuNH_2_ concentrations. This observation was interpreted as CdSe NCs offering a diversity of binding sites with different displacement energies.^429^ A similar approach was later used to probe the binding strength of MX*_n_* moieties to a range of NCs, which invariably showed that NC surfaces are intrinsically heterogeneous and offer binding sites with a range of displacement or desorption energies.^429–433^ In particular, for colloidal NPLs—2D NCs with an atomically precise thickness—it was shown that the stronger binding sites correspond to the center of a crystal facet, while the edges and corners that end these facets offer the weaker binding sites.^434^

**Figure 12:**
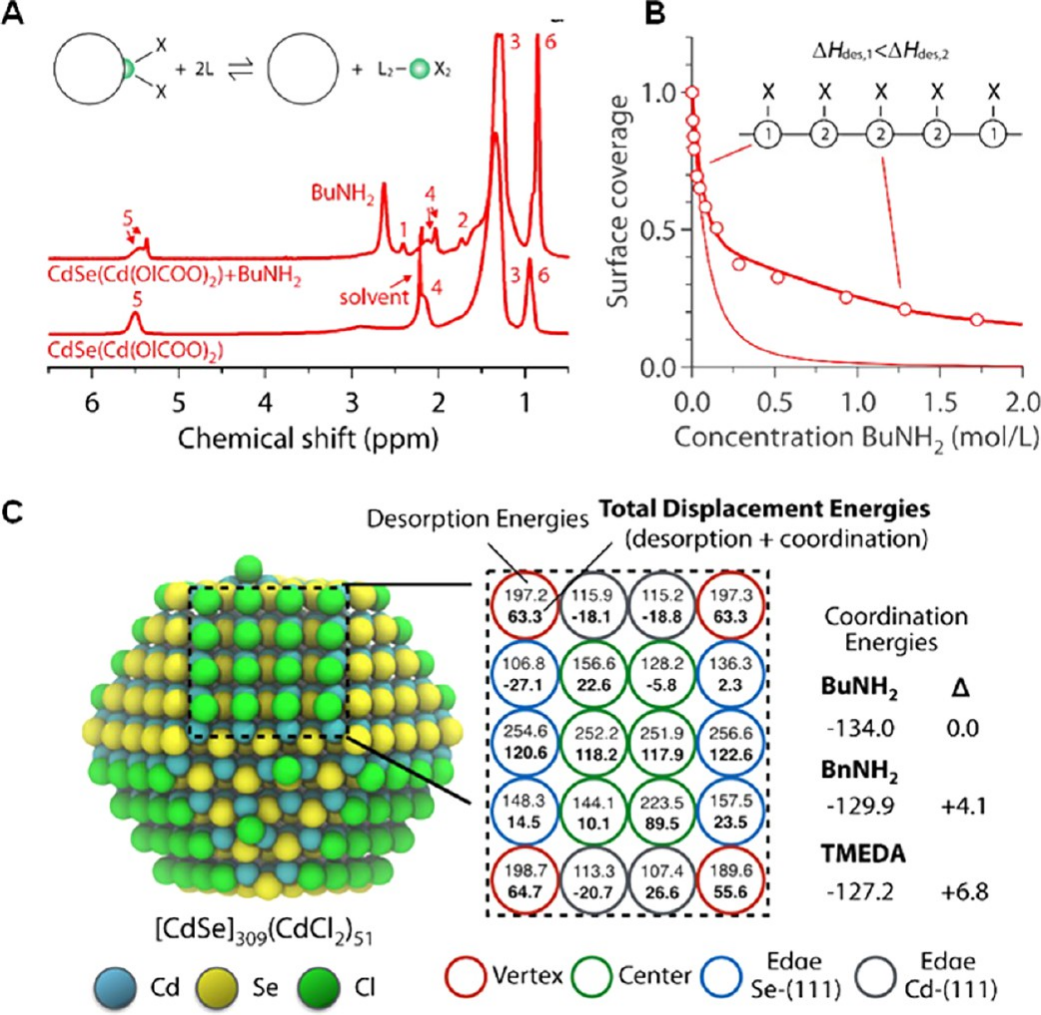
Ligand displacement by Lewis bases. (A) ^1^H NMR spectra of a dispersion of oleate-capped CdSe NCs (bottom spectrum) before and (top spectrum) after addition of butylamine. The broad resonances can be assigned to bound oleate, while the appearance of accompanying narrow resonances is indicative of the displacement of cadmium oleate from the NC surface through complexation with butylamine. (B) Surface coverage of oleate as a function of butylamine concentration, in comparison with expected isotherms assuming (thin line) all identical binding site and (bold line) two sets of binding sites with different displacement energy. (C) Calculated, site-dependent displacement energy of CdCl_2_ from the (100) facet of a [CdSe]_309_(CdCl_2_)_51_ model NC. Chloride is used as a substitute for oleate in DFT calculations. Adapted from ref 429. Copyright © 2018 American Chemical Society.

### Passivation of Surface-Localized Electronic States

Desorption of ligands from NC surfaces is commonly seen as creating dangling bonds that can trap photogenerated charge carriers and thereby quench PL.^415^ The study of ligand displacement turned this hand-waving argument into a tangible concept that helped to understand, control, and undo the formation of such trap states. Here, a key step was the combination of the experimental observation that CdX_n_ displacement from CdSe NC abruptly quenched the PL,^416^ and the computational finding that undercoordinated surface selenium invariably leads to surface-localized states.^335^ This insight spurred a variety of studies in which the opposite process—exposure of NCs to excess metal salts to eliminate undercoordinated surface anions—was successfully used to enhance the PL efficiency of semiconductor NCs, in some cases up to 100%.^435,437^ Alternatively, the PL of colloidal CdSe NPLs was made robust under ligand displacement by overcoating the CdSe edges by a crown of CdS, a wider-bandgap material that prevents excitons within the CdSe core from reaching defects at the CdS outer edge.^434^ While the electronic passivation of core NCs by surface ligands may not be the best approach in view of long-term stability, the extension of these surface passivation schemes to such core/shell systems offers a clear path forward to NCs with efficient and stable PL (Figure 13).^436^

**Figure 13:**
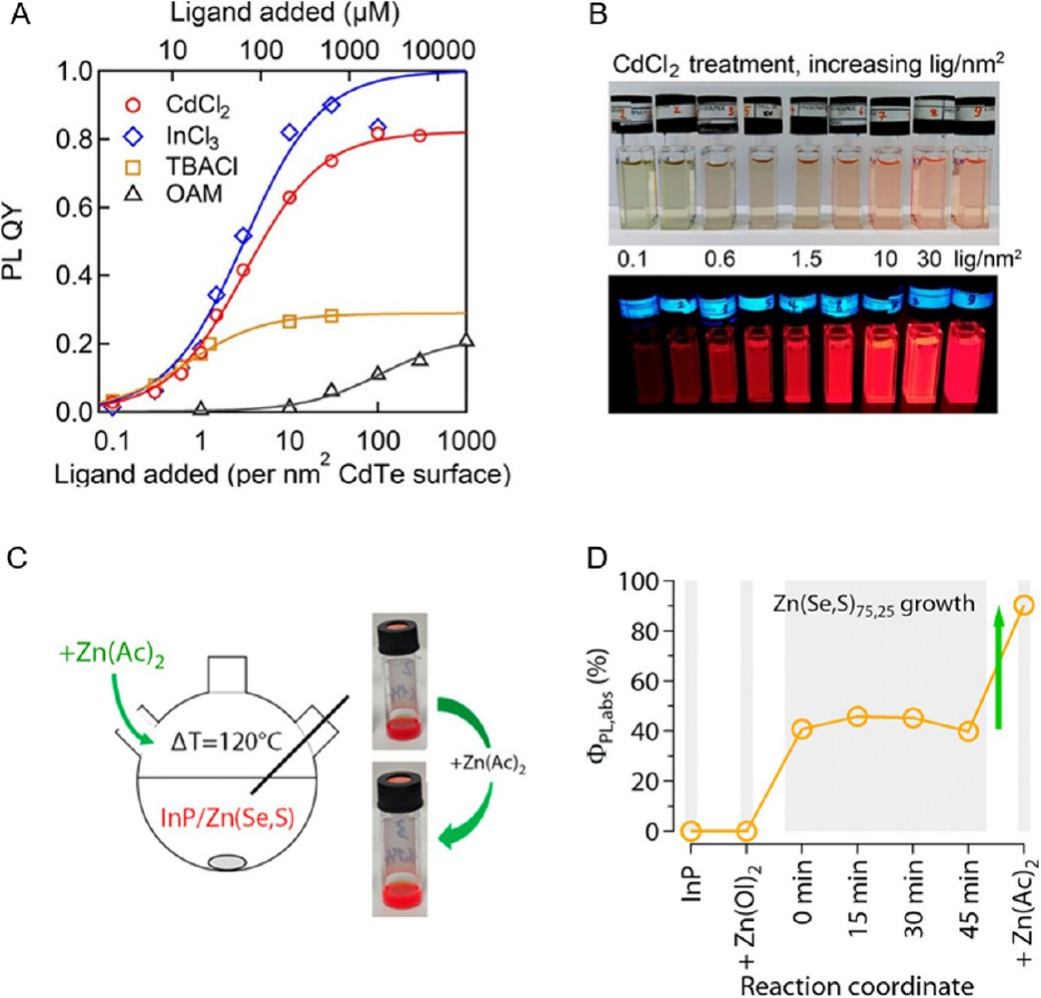
Impact of metal salt binding on PL efficiency of core and core/shell NCs. (A) PL QY as a function of the number of ligands added per nm^2^ of CdTe surface area. TBACl = tetrabutylammonium chloride; OAM = oleylamine. (B) Pictures of cuvettes containing CdTe exposed to different amounts of CdCl_2_. Ligand displacement by Lewis bases. Panels (A) and (B) reproduced from ref 435. Copyright © 2018 American Chemical Society. (C) Outline of a synthetic procedure in which InP/ZnSe NCs are treated with zinc acetate after synthesis. (D) Variation of the PL QY during the synthesis of InP/ZnSe NCs, where the final addition of zinc acetate increases the PL QY from 40 to 90%. Adapted from ref 436. Copyright 2024 American Chemical Society.

### Localizing Ligands on NC Surfaces

In large part, the current understanding of NC surface chemistry is the result of the judicious use of chemical analysis methods. In particular, the application of solution NMR spectroscopy^346,438,439^ to study surface ligands has been highly instrumental.^440^ By means of diffusion ordered spectroscopy, solution NMR enables researchers to distinguish between bound ligands and residual reagents, and to assess, thereby, sample purity. As a result, NMR spectroscopy has become a standard tool to identify surface-bound ligands, quantify ligand surface concentrations, and monitor ligand-exchange reactions *in situ*. Moreover, more recent work highlighted that ligand resonances are also affected by the heterogeneous character of the ligand shell. Even the first studies analyzing surface ligands by solution NMR pointed out the heterogeneous broadening of ligand resonances, possibly reflecting a multitude of chemical environments.^441,442^ More recent work showed that this variety of chemical environments is directly linked to the ligand–solvent interaction, where well-solvated ligands give rise to much narrower resonances than poorly solvated ligands.^438^ Furthermore, by analyzing the recovery of spectral holes formed by selectively saturating part of a ligand resonance, different ligand pools could be identified within a single broad resonance that reflects less-solvated facet localized and more solvated edge-localized ligands.^443^ Such studies provide a detailed map of the NC surface, where ligand localization, ligand displacement and trap-state formation appear as highly related phenomena that must be controlled in full to enhance long-term operational stability of NCs.

### Theoretical Investigations

A large variety of theoretical methods have been employed to study QDs, with effective-mass, *k·p*,^444^ and pseudopotential theory^445^ among the early contenders. While successfully revealing many of the important size- and shape-dependent electronic properties, such methods emerging from the realm of solid-state physics are less well suited to capture the rich and intricate surface chemistry of colloidal QDs. In this respect, calculations based on atomistic models represent a compelling alternative. Until the early 2010s, efforts to model QDs were mainly modeled using tight-binding calculations^446,447^ (TB) and DFT calculations, with the former being predominant, especially for large structures. The TB method featured surface passivation with pseudohydrogen atoms to emulate bulk coordination also at the surface of QDs, which were typically modeled with a spherical shape obtained by cleaving the bulk material. This passivation ensured neutrality by adjusting the pseudohydrogen charge to match the inorganic core’s charge. TB was essential for exploring, among others, the effect of quantum confinement^447^ and dynamics such as the Auger effect and electron–phonon interactions.^448,449^ Nonetheless, it overlooked the crucial influence of the surface, key to understanding core-ligand interactions and the emergence of surface traps.

Up until 2011, atomistic models of QD described using DFT primarily relied on small stoichiometric models. A notable example is the Cd_33_Se_33_ model, introduced by Puzder in 2004.^450^ This model, and slight variations of it, was extensively used by various research groups, including those led by Prezhdo and Kilina, to study phenomena such as nonradiative recombination, phonon bottlenecks, and the impact of L-type ligands on electronic structure.^451–454^ However, these models were mostly small QD clusters, lacking clear surface facets and exhibiting large reorganization energies. A significant breakthrough occurred in 2011 when Voznyy first modeled a nonstoichiometric CdSe QD (Figure 14A).^455^ This model was more aligned with the nonstoichiometry observed in experiments. It featured an excess positive charge in the core, which was balanced by carboxylate ligands, mimicking the oleate ligands used in experimental settings. Importantly, this QD model also presented well-defined facets, effectively representing a nanocrystallite. This development catalyzed a series of studies, particularly focusing on CdSe and PbSe, by the same research group.^456–458^ These studies paved the way for more advanced modeling of QDs. The success was partly due to the fact that these models could be scaled in size to match experimental observations.

**Figure 14:**
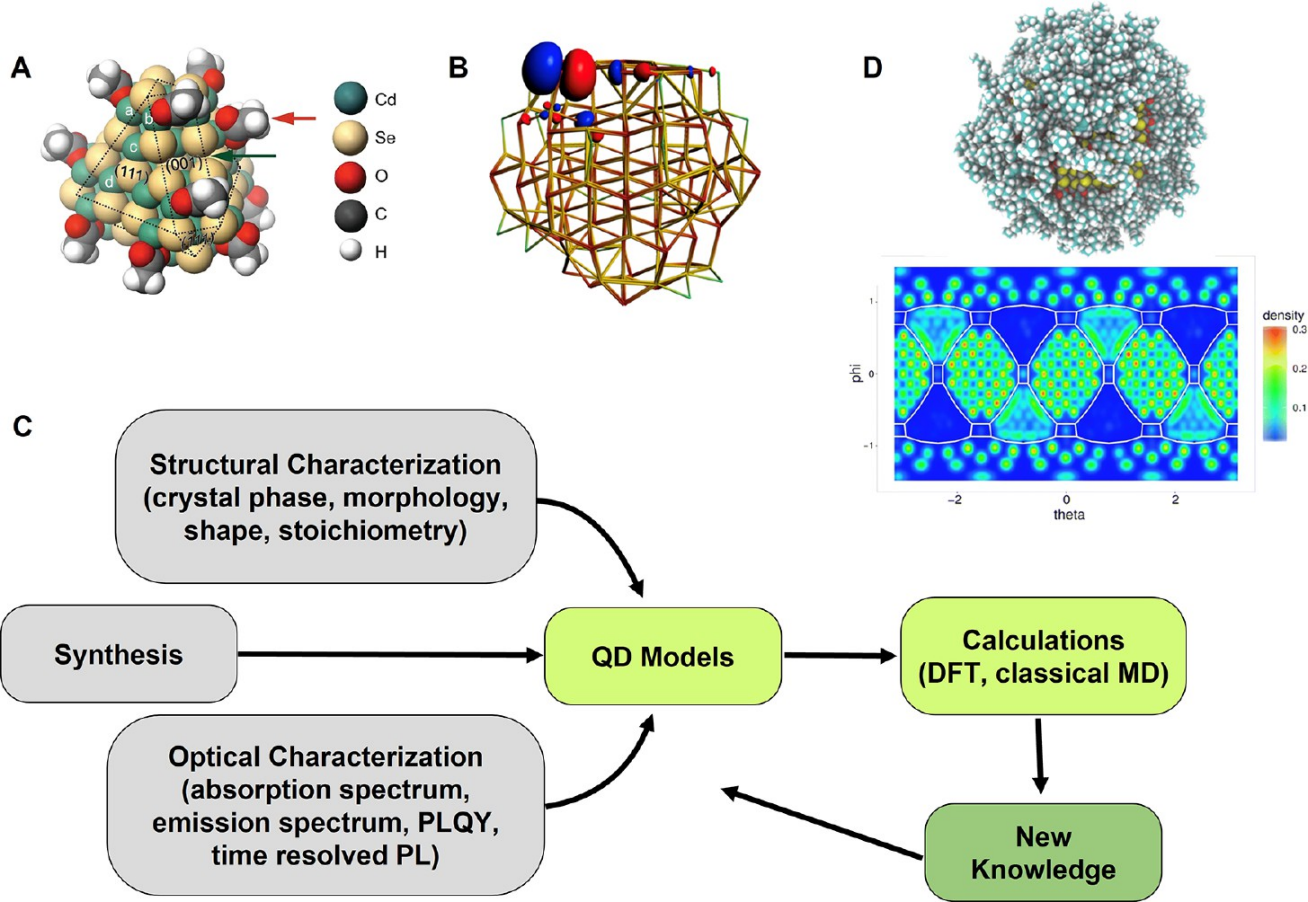
(A) First nonstoichiometric CdSe model as proposed by Voznyy et al. Reproduced from ref 455. Copyright © 2011 American Chemical Society. (B) Molecular orbital corresponding to a trap state localized on a Se dicoordinated atom on a CdSe QD model. The trap state emerged upon displacement of a CdCl_2_ Z-type ligand pair. Reproduced from ref 335. Copyright © 2017 American Chemical Society. (C) Typical workflow procedure to prepare a QD model for any type of semiconductor, from II–VI to perovskite QDs. (D) (top) QD model of 4 nm passivated with oleate ligands with a surface density of about 3.5 ligands nm^–2^. Extensive classical MD simulations have been carried out to understand where the ligands most likely bind; (bottom) 2D map of a cuboctahedron CdSe QD. It represented a density map showing the most probable positions where the ligands bind. Reproduced with permission from ref 468. Copyright © 2023 Royal Society of Chemistry.

In the same time frame, notable work was conducted by Zherebetskyy and co-workers, who explored ligand passivation using oleate ligands on 5 nm PbSe nanostructures.^459^ Drawing inspiration from Owen and colleagues,^416^ Houtepen, Infante et al., in 2017, undertook a comprehensive study on the effects of different ligands (L-, X-, and Z-type) on trap formation in II–VI semiconductor QDs.^335^ Their findings highlighted that removing surface Z-type ligands induces the formation of deep trap states. This process stabilizes nonbonding, dicoordinated chalcogen atoms on the QD surface (Figure 14B). Such changes were linked to reductions in PL QY, as previously observed by the Owen group when Z-type ligands were removed. Furthermore, the study proposed postsynthetic treatments with Z-type ligands as an effective method for mitigating these surface traps, a technique subsequently validated in later research.^435^ This advancement sparked a series of studies that merged computational models of atomistic QD structures with experimental approaches to analyze the surfaces of various QDs. Notable contributions in this area were made by Giansante et al.,^460,461^ who developed efficient models for ligand-capped, nonstoichiometric PbS NCs. Concurrently, Nazdani et al. conducted research to quantify the phononic properties of PbS, revealing that phonon modes exhibit lower energy at the surface, attributed to mechanical softness.^394^ Advancements in computer architectures have enabled DFT studies on 4 nm QDs, revealing unexpectedly small HOMO–LUMO gaps, contradicting quantum confinement. This issue stems from surface facets, which in bulk are stabilized by reconstructions. Applying these reconstructions to QD models restores expected band gaps.^462^ Other recent developments within DFT modeling of QDs include reconstructing “fuzzy” QD band structures through Bloch orbital expansion from real-space orbitals to k-space. This approach provides a fresh perspective on finite QD clusters, offering deeper insights into identifying surface traps and determining core–shell energy alignment in confined systems, independent of bulk-derived values.^463^

The modeling of QDs has significantly advanced in recent years. Currently, the standard approach to QD modeling typically involves either a combined experimental-theoretical effort within the same study or sequential efforts, as illustrated by the work on colloidal CsPbBr_3_ perovskite QDs. The synthesis of these QDs was reported by the Kovalenko group in early 2015,^310^ and the first model was published by ten Brinck and Infante in early 2017.^328,338^ This progression marks a notable acceleration in the field, contrasting with the 20-year gap between the initial hot-injection synthesis of CdSe QDs and the emergence of their first nonstoichiometric model.

The standard method for constructing atomistic QD models involves a consistent collaboration with experimentalists on a case-by-case basis. A combination of different elemental analyses, such as SEM- energy dispersive X-ray spectroscopy and X-ray photoelectron spectroscopy, offers insights into the stoichiometry of the material. This is pivotal in determining the QD termination, for instance, by an excess of metal cations or anions. Additionally, TEM measurements reveal the exposed facets of the QD. Subsequently, a QD model is assembled that aligns with the crystalline phase, stoichiometry, and morphology observed in the experiments. Maintaining the charge-balance condition is critical as it ensures that all ions in the material are in their most stable thermodynamic oxidation state. Overlooking this aspect carries the risk of artificial n- or p-doping of the material, yielding poorly defined QD models that significantly diverge from experimental findings. Once the QD model is prepared, the structure undergoes relaxation, and its electronic structure is computed. The entire procedure is outlined in a workflow presented in Figure 14C. On these refined models, further studies can be conducted. These may range from a simple analysis of the electronic structure to more complex investigations. For example, one might examine the effects of detaching surface ligands as observed in experiments, or study time-dependent processes like the rate of trapping and nonradiative decays.

A significant limitation of DFT is its high computational cost. This limitation hinders the inclusion of solvent and full-sized ligand molecules in the simulations, which are critical for understanding the roles of ligand–ligand, ligand-core, and ligand-solvent interactions in stabilizing QDs in organic solvents. It also impedes the execution of long-time scale simulations, such as those required in molecular dynamics. Due to these constraints, it becomes essential to consider more computationally efficient alternatives, like methods based on classical force fields (FFs). An inherent challenge with classical FFs, however, is the development of accurate FF parameters. A notable advancement in this area was made following Rabani’s work on CdSe QDs.^464^ In 2017, Cosseddu and Infante developed and implemented an Adaptive Rate Monte Carlo algorithm, which was instrumental in obtaining precise FF parameters for modeling core–core and core–ligand interactions in CdSe QDs.^465^ A significant advantage of this method is its ability to include ligands with long alkylic chains directly in the QD model and to add solvent molecules, facilitating the simulation of ligand-passivated QDs in various solvents. Thanks to these developments, as of this year, a diverse range of FF parameters are available, extending from II–V to III–V and lead-halide perovskites.^466,467^ Several published examples demonstrate the effectiveness of these parameters, providing detailed descriptions of the probable locations of ligands on CdSe and InAs^468,469^ and, more recently, organic ligands on perovskite QDs.^387^

A significant limitation of traditional FFs is their inability to capture electronic-structure details despite their capability to facilitate lengthy simulations up to the microsecond scale. Conversely, DFT methods offer valuable electronic-structure insights but are restricted to much shorter time scales, typically only a few tens of picoseconds. The recent advent of machine-learning-based force fields (ML-FFs) promises to bridge this gap.^470^ These innovative ML-FFs are poised to deliver DFT-level structural and electronic insights with computational speeds that are only slightly slower than classical FFs. With the development of ML-FFs tailored for QDs, we anticipate being able to extend the simulations to the nanosecond range. This advancement is crucial as it would enable one to accurately analyze phenomena like nonradiative rates, which can occur also in the hundreds-of-picoseconds time scale, and emission lifetimes, usually in the nanosecond time scale. Such capabilities contribute to the growing sophistication of calculations in the QD research field.

## Assembly

### Superlattices (SLs)

Colloidal steric-stabilized NCs coated with hydrocarbon ligand chains offer opportunities in the development of artificial solids with tailored properties due to their ability to self-organize with nanoscale precision into long-range ordered structures known as NC SLs. The past decade has seen a rising interest in shape-directed assembly, as discussed below.

The formation of NC SLs involves a complex equilibrium between enthalpic and entropic interactions,^471–475^ both of which contribute to the Gibbs free energy of the system. Enthalpic forces comprise weak dipole–dipole, Columbic, magnetic, and van der Waals forces between inorganic cores and ligands.^473,474^ The contribution from entropy includes the gain of free volume entropy^471^ associated with the space available for each NC to perform local motions, depletion forces^476,477^ and entropy associated with ligand configurations.^478^ In the case of entropy-driven crystallization, the system forms a periodic structure with the maximized packing density, which is, in this context, the volume fraction occupied by the NC core and ligand shell. Self-assembly of monodispersed spheres promotes the formation of the densest possible structure, typically favoring face-centered cubic and hexagonal close-packed arrangements.^473,479^ Recently, *in situ*, liquid cell TEM with single-particle resolution was utilized to image the formation of assembly in real-time from sterically stabilized Au NCs in nonpolar solvents.^480–482^ Multistep crystallization of Au NCs into SL, involving gas state, cluster state, polycrystalline, and single crystalline solid states, was thus unveiled.

Binary mixtures comprising combinations of semiconductor, magnetic and metallic spherical NCs can assemble into over 20 lattices isostructural with various atomic, ionic, and intermetallic compounds,^473,483,484^ or even in aperiodic quasicrystalline phases.^485–487^ Most of these structures would be estimated to have lower packing density than single-component face-centered cubic and hexagonal close-packed lattices, if the spheres are considered exclusively as hard particles. However, higher packing density is seen experimentally (by careful analysis of the inter-NC distances in diverse SLs)^488^ and also results from the deeper theoretical analysis that considers the ligand deformability. In particular, the orbifold topological model^489^ accounts for the collective bending of the ligands away from the axis of contact between NCs with the formation of vortices, substantially increasing the structure’s overall packing density of the lattice.

Advancements in the colloidal synthesis of NCs and their surface modification have enabled repeatable synthesis of a range of size- and shape-controlled NC building blocks. The particle morphology can thus be harnessed to explore a broader structural space for SLs, beyond the lattices known from the atomic world. Single-component NC SLs with anisotropic building blocks include periodic^490–496^ or aperiodic structures.^492,497^ The structure behavior of assemblies from shape-anisotropic NCs can be explained by directional entropic forces, which lead to dense local packing.^498^ During the assembly process, such NCs tend to align along the flat facets to maximize entropy and minimize the system’s free energy, leading to assemblies with long-range order.^499^ Various binary NC assemblies had been observed combining spherical and nonspherical NCs, such as triangular nanoplates,^483^ NRs,^500–502^ branched octopods,^503^ nanowires.^504^ At the same time, only a few reports concern binary NC SLs made exclusively from anisotropic NCs. For instance, columnar SLs from the mixture of nanodisks and NRs,^505^ thin-film SLs from PbTe nanocubes and triangular LaF_3_ nanoplates,^506^ GdF_2_ rhombic nanoplates and tripodal Gd_2_O_3_ nanoplates^507^ (Figure 15A−D).

Recently developed lead halide perovskite NCs in the form of sharp, monodisperse cubes make for very attractive shape-engineered building blocks for self-assembly.^310^ Lead halide perovskite nanocubes have been reported to form single-component superstructures adopting a simple-cubic packing arrangement.^373,508–514^ Remarkably, these SLs displayed an intriguing phenomenon known as superfluorescence (SF) at cryogenic temperatures, which manifests itself as a red-shifted emission band and a remarkable 20-fold acceleration in radiative decay under high excitation density. The discovery ignited the exploration of multicomponent SLs with perovskite NCs to achieve precise control over the positioning and orientation of these coherent light emitters.^515–517^ Obtained results differed significantly from the outcomes observed in the self-assembly of all-spherical NCs.

Specifically, when CsPbBr_3_ nanocubes are coassembled with large spherical dielectric NaGdF_4_ or magnetic Fe_3_O_4_ NCs, the binary ABO_3_-type SL isostructural with cubic CaTiO_3_ perovskite structure forms with cubes occupying B and O sites (Figure 15E). The formation of this perovskite-type SL had not previously been observed in the colloidal crystallization of steric-stabilized NCs emerging due to the cubic shape of lead halide perovskite NCs. According to orbifold topological model, the distinctive sharpness of nanocubes promotes the bending of capping ligands around corners and edges and forming ligand vortices when two NCs approach each other, leading to the much higher packing density of the lattice compared to the hard-sphere case. Utilizing the nonequivalence orientation of B and O cubes, the third component, truncated cubic PbS NCs, was incorporated on a slightly larger B-site, yielding a ternary ABO_3_-type SL.^515^ Beyond perovskite-type SLs, structures of AB_2_ and ABO_6_ compositions and three SL-types identical to those commonly found for binary mixtures of spherical NCs (NaCl, AlB_2_, and CuAu-types) can be formed in which perovskite nanocubes serve as a small component of the lattice.^517^ Moreover, coassembly of sharp, nontruncated CsPbBr_3_ nanocubes with thin LaF_3_ nanodisks (1.6 nm in thickness, 6.5–28.4 nm in diameter) yields columnar structures with AB, AB_2_, AB_4_, and AB_6_ stoichiometry (Figure 15F). These SLs comprise columns of disks and cubes forming a 2D periodic pattern or 3D structures that feature face-to-face contacts between cubes and disks of similar size.^516^ Combination of nanocubes with large and thick NaGdF_4_ nanodisks gives rise to the orthorhombic SL resembling CaC_2_ structure with pairs of CsPbBr_3_ NCs on one lattice site (Figure 15G).^517^ Generally, the cubic shape and facile ligand-deformability at the vertices and edges yield denser lattice packing than in all-sphere systems. Various binary SLs with large volume fractions of perovskite NCs exhibit characteristic signatures of SF arising from the coherent coupling of emission dipoles in the exited state at cryogenic temperature. The formation of multicomponent perovskite NC-only SLs, using CsPbBr_3_ NCs of different sizes and shapes (dodecahedra and cubes) as building blocks, facilitates efficient NC coupling and Förster-like energy transfer from strongly to weakly confined CsPbBr_3_ NCs, along with enhanced exciton diffusivity compared to single-component NC assemblies.^518^

**Figure 15:**
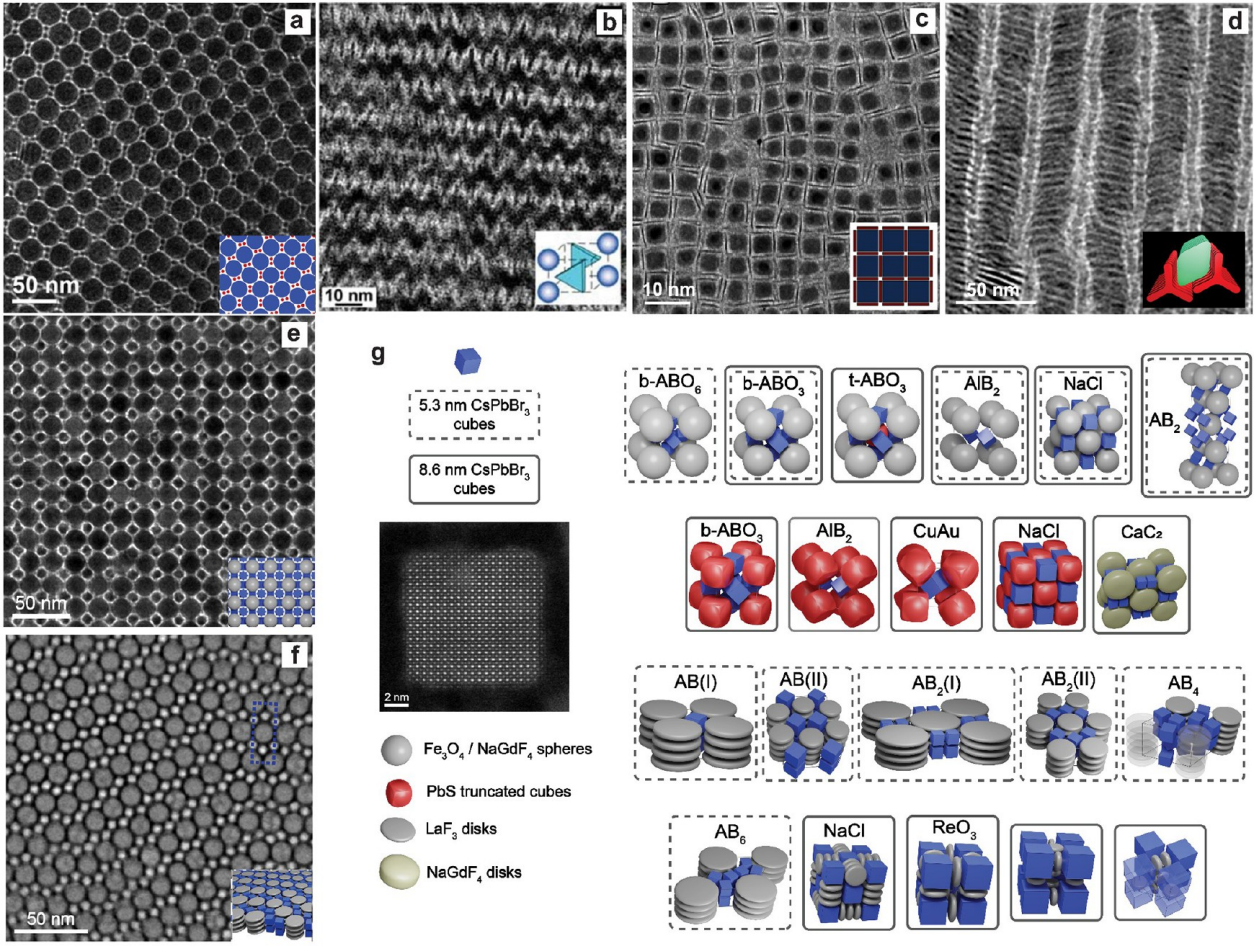
Examples of binary NC SLs comprising anisotropic shape NCs: TEM images of binary SLs formed by coassembly. (A) LaF_3_ nanodisks and CdSe/CdS NRs (AB_2_-type). Reproduced from ref 505. Copyright © 2015 American Chemical Society. (B) LaF_3_ triangular nanoplates and Au nanospheres. Reproduced with permission from ref 483. Copyright © 2006 Springer Nature. (C) PbTe nanocubes and LaF_3_ triangular nanoplates. From ref 506. Copyright © Elbert et al., some rights reserved; exclusive licensee AAAS. Distributed under a CC BY-NC 4.0 license http://creativecommons.org/licenses/by-nc/4.0/. Reprinted with permission from AAAS. (D) Gd_2_O_3_ tripodal nanoplates GdF_3_ rhombic nanoplates. Reproduced from ref 507. Copyright © 2013 American Chemical Society. (E) CsPbBr_3_ nanocubes and NaGdF_4_ nanospheres. Reproduced from ref 517. Copyright © 2022 American Chemical Society. (F) High-angle annular dark field scanning transmission electron microscopy image of columnar AB_2_(II)-type binary SL assembled from CsPbBr_3_ nanocubes and LaF_3_ nanodisks. Reproduced from ref 516. Copyright © 2021 American Chemical Society. (G) Diversity of binary SLs obtained from CsPbBr_3_ nanocubes combined with nanospheres, truncated nanocubes, and nanodisks. Reproduced from ref 517. Copyright © 2022 American Chemical Society.

Creating multicomponent NC SLs comprising anisotropic NCs involves a highly complex interplay of various parameters. In this context, precise control over the shape of building blocks is a guiding factor in designing distinctive functional materials with collective properties. This approach unlocks a remarkable array of multicomponent NC SLs previously unobserved in all-spherical systems. Further developments in rationalizing multicomponent SLs made of various shapes of NCs via modeling and theory will provide guidance for the design of advanced functional materials.

#### Liquid-Phase TEM Imaging of NC Self-Assembly

While electron microscopy has been instrumental in elucidating the final structures formed through NC self-assembly, and reciprocal-space techniques like SAXS have provided valuable insights into the NC self-assembly process, there’s still much to explore regarding the kinetic pathways and mechanisms involved. Understanding these dynamics is crucial for fine-tuning self-assembly processes and designing materials with tailored properties. Real-time video microscopy has been a prevalent method for studying the assembly of micron-sized colloids. However, techniques for investigating the self-assembly dynamics of colloidal NCs were limited until the invention and commercialization of liquid-phase TEM tools, including the microfabricated liquid cells which separate the liquid environment from the vacuum inside the TEM, and the liquid cell holders which not only provide mechanical support to the liquid cells but also allow liquid flows into the liquid cells before and during imaging.^519–524^

Liquid-phase TEM has enabled observation of the dynamics of various NC assemblies in aqueous solutions.^525–530^ In 2013, Liu et al. reported the liquid-phase TEM imaging of positively charged gold nanospheres in aqueous solution forming 1D chains.^525^ Later, Alivisatos and co-workers observed the tip-to-tip assembly of positively charged gold NRs in aqueous solution using liquid-phase TEM (Figure 16A).^527^ Tracking and analysis of numerous individual gold NR trajectories revealed the anisotropic interaction potential between charged gold NRs leads to the tip-selective attachment. Chen and co-workers utilized low-dose liquid-phase TEM to observe the linear chain formation of negatively charged gold triangular nanoprisms in the tip-to-tip configuration (Figure 16B).^528^ Tracking the size distribution of prism chains over time revealed that the chain followed the reaction-limited step-growth polymerization. Later, the same research group reported the liquid-phase TEM imaging of extended SL formation from negatively charged gold triangular nanoprisms.^530^ The electron-beam irradiation initiated the assembly process by increasing the local ionic strength through radiolysis, thereby screening the electrostatic repulsions among nanoprisms. Particle tracking analyses and computer simulations revealed that the translational ordering of the SLs arose from orientational randomness among the nanoprisms within individual columns and the assembly followed a nonclassical crystallization pathway involving an amorphous, dense intermediate phase.

**Figure 16:**
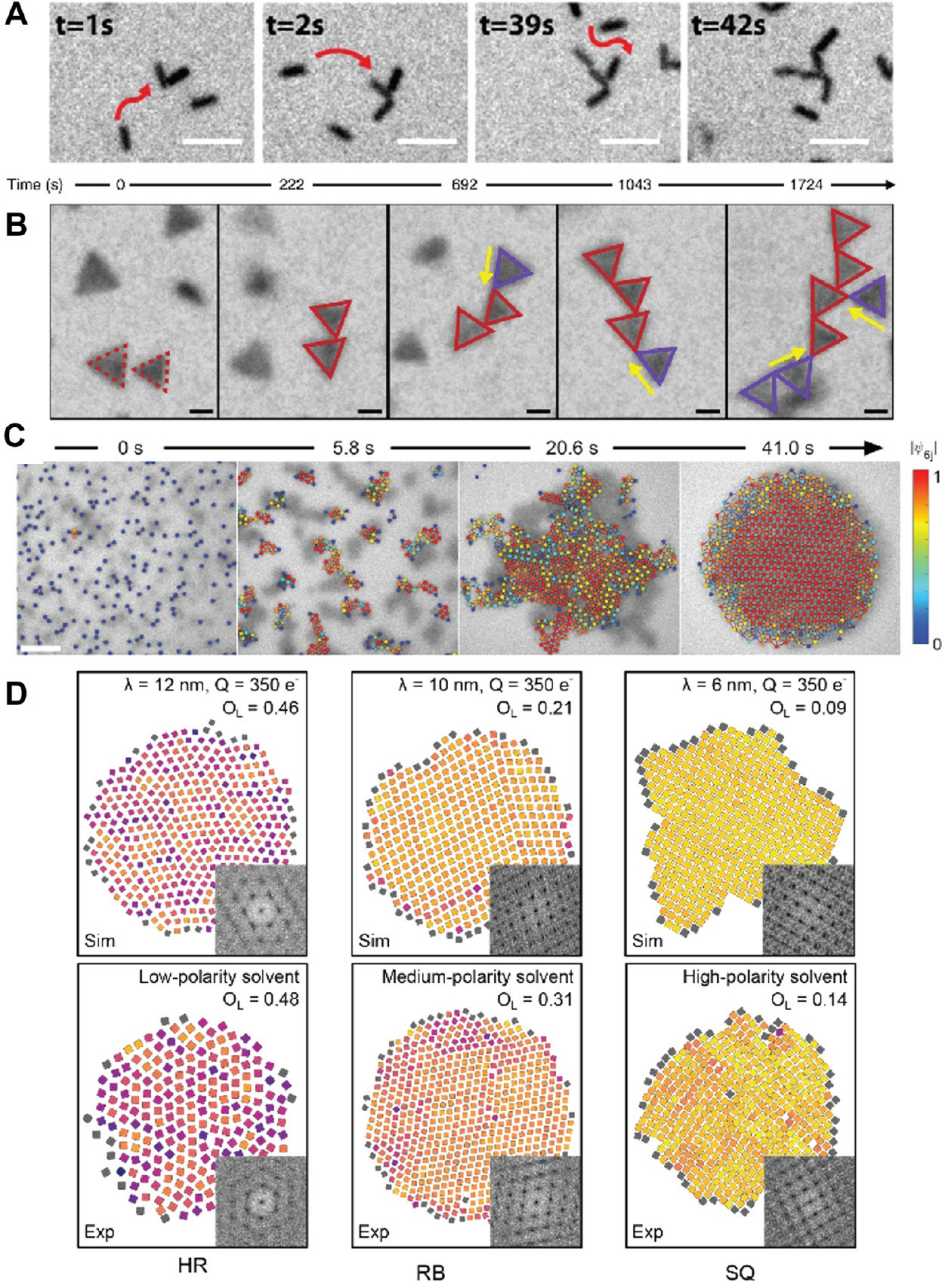
*In situ* liquid-phase TEM studies of nanoparticle self-assembly. (A) Time-lapse TEM images showing tip-to-tip attachment of gold NRs. Reproduced from ref 527. Copyright © 2015 American Chemical Society. (B) Time-lapse TEM images showing chain growth of gold triangular nanoprisms. Reproduced with permission under CC BY 4.0 from ref 528. (Copyright © 2017 Springer Nature, open access). (C) Time-lapse TEM images showing the self-assembly process of gold nanospheres, with NC centroids color-coded according to the modulus of 6-fold bond-orientational order parameter |ψ_6*j*_|. Reproduced from ref 480. Copyright © 2022 American Chemical Society. (D) Snapshots from simulations and liquid-phase TEM experiments with particles colored according to the offset order parameter *Q*_L_. Gray particles have fewer than three nearest neighbors and were excluded from the *Q*_L_ calculation. The insets are fast Fourier transform patterns of a subregion of the image to highlight the symmetry of a single-crystalline grain. Simulation parameters (solvent conditions) are indicated above each simulation (experimental) snapshot. λ, electrostatic screening length; Q, charge per nanocube; Sim, simulation; Exp, experiment. Reproduced with permission from ref 536. Copyright © 2024 Springer Nature. Scale bar, 100 nm (A), 50 nm (B), 500 nm (C).

In addition to studying self-assembly in aqueous solutions, liquid-phase TEM has proven valuable for investigating NC assembly in nonaqueous environments.^480,481,531–536^ In 2012, Alivisatos and co-workers reported liquid-phase TEM imaging of drying-mediated 2D NC SL formation.^531^ Trajectory analysis and coarse-grained modeling suggested that the Pt NCs exhibited slow diffusion, and the assembly was primarily propelled by capillary forces and solvent fluctuations. Later, Zheng and co-workers observed that the *in situ* formed PtFe_3_ NCs in a viscous medium self-assemble into chains, which then clump and fold into a loosely packed hexagonal SL.^534^ Particle tracking and diffusion analyses suggested that long-range dipolar forces and van der Waals interactions are the driving forces for the initial chain formation and later 2D SL formation, respectively. Recently, Ye and co-workers employed polymer-grafted NCs suspended in various organic solvents as model systems to investigate NC self-assembly into highly ordered 2D SLs with liquid-phase TEM.^480,536^ The electron-beam irradiation activates NC motion and modulates NC diffusivity, while the solvent’s nature largely dictates NC interactions and self-assembly pathways. A multistep crystallization pathway comprising four distinct stages—gas state, cluster state, polycrystalline state, and single crystalline state—was observed in the Au nanosphere assembly as well as the Au nanooctahedron assembly (Figure 16C).^480^ In addition, the formation of square-like (SQ), rhombic (RB), and hexagonal rotator (HR) phases was observed in the gold nanocube assembly as the solvent polarity decreased with an increasing ratio of octane to butanol (Figure 16D).^536^ Deep learning assisted-image segmentation and multiorder-parameter analysis revealed that the evolution of lattice translation order and particle orientational order was largely decoupled during the assembly of the hexagonal rotator phase, while strong coupling was observed for the square-like phase. In contrast, the degree of coupling varied at different stages of the assembly process for the rhombic phase. They also demonstrated real-time control of solid–solid phase transitions in the gold nanocube assembly *via in situ* rapid solvent exchange.

Despite significant advancements in the study of nanoparticle self-assembly using liquid-phase TEM, there are several challenges and perspectives to be further explored. First, both electron beam and solvents have been utilized to initiate and guide the *in situ* self-assembly of NCs in liquid-phase TEM. However, more understanding of the interplay between the electron beam, solvent, and NCs are needed. Systematic experimental exploration with varied dose rates and solvent compositions in combination with computer simulations could shed light on the underlying mechanisms of observed NC dynamics. Second, *in situ* self-assembly of NCs with different shapes and compositions have been studied using liquid-phase TEM; however, all the studies only use one type of NCs to form single-component SLs. In contrast, a library of multicomponent NC SLs, especially binary NC SLs, has been achieved in *ex situ* experiments. Therefore, direct liquid-phase TEM imaging of the nucleation and growth of multicomponent NC SLs can provide valuable insights into the kinetic pathways underlying their formation. Third, the liquid-phase TEM studies of NC self-assembly produce large and complex data sets, which pose great challenges in the image processing and quantitative data analysis. Machine learning, especially deep learning, has been utilized for the image segmentation of the liquid-phase TEM images. Further integration of artificial intelligence with liquid-phase TEM can be explored to achieve automated and high-throughput data acquisition, processing, and analysis.

### Gel Assembly

NCs have been assembled into porous gel networks through the controlled introduction of attractive interactions between what are otherwise stably dispersed colloidal particles with overall repulsive interparticle interactions.^537,538^ Traditionally, NC gels have been formed by partial displacement of their stabilizing surface ligands, which typically leads to the formation of strong, largely irreversible bonds directly between the inorganic cores.^539^ For example, desorption of alkanethiols from metal sulfide NCs follows their controlled oxidation, leading to the formation of disulfide bridges between the NC surfaces, fusing them into a highly porous network. After careful solvent removal, aerogels may be formed that retain the optical signatures of quantum confinement in the case of semiconductor NC building blocks or that serve as efficient electrocatalysts in the case of transition metal NCs.^540–542^

Conversely, to make reversible gel networks that can respond to varied physical and chemical stimuli, forming chemical links that bridge between functionalized ligands on neighboring NCs has emerged as a versatile strategy.^543–545^ Such NC gels have been synthesized from both semiconductor and metal NCs and the linker approach enables ready intermixing of compositionally distinct building blocks.^546,547^ Linking can be accomplished with dynamic covalent bonds, for example, by adjoining aldehyde-terminated ligands with a bifunctional hydrazide linker to create a bis-hydrazone bridge.^545^ Gelation was reversed by displacing the linkers with a monofunctional hydrazide, thus capping the functional groups on the ligands and deconstructing the network to recover a flowing NC dispersion.^545^ Metal coordination links are another option for dynamic bonds to assemble NC gels. Either inorganic capping ligands (*e*.*g*., chalcogenidometallates clusters)^548,549^ or organic ligands bearing suitable functional groups^543,544^ can be linked by a variety of metal ions to form 3D NC networks. A strong chelator like ethylenediaminetetraacetic acid can extract the linker ions to recover the flowing dispersion.

By linking terpyridine-functional ligands with cobalt ions in the presence of excess chloride, thermoreversible gels were prepared from plasmonic ITO NCs (Figure 17A).^544^ The IR plasmonic absorption of the NCs shifted substantially to lower frequency upon assembly, and the shift was fully recovered when the system was heated above the gelation temperature, a process that was highly cyclable (Figure 17B). ITO NCs make ideal building blocks for reversible assembly; having excellent chemical and structural stability, they resist the irreversible fusing that commonly plagues conventional plasmonic metal NCs like gold. The cobalt ligand exchange equilibrium governing the NC linking could be shifted by varying the amount of chloride in solution, thus chemically tuning the gelation temperature (Figure 17C):

$$\underset{\mathrm{NCs}\;\mathrm{linked}}{{\left[\mathrm{Co}{\left(\mathrm{Tpy}\right)}_{2}\;\right]}^{2+}\;+4\;{\mathrm{Cl}}^{-}\;}⇌\underset{\mathrm{Ncs}\;\mathrm{dispersed}}{2\;\mathrm{Tpy}+{\left[{\mathrm{CoC}l}_{4}\;\right]}^{2-}\;}$$

**Figure 17:**
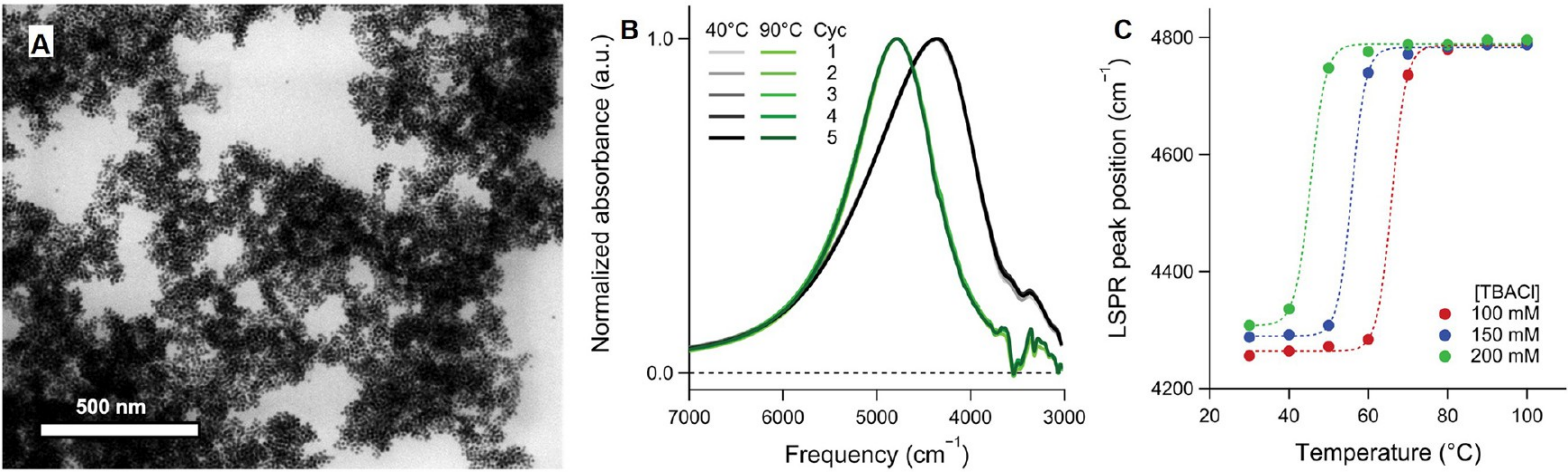
Thermoreversible Indium tin oxide (ITO) NC gels assembled by metal coordination links. (A) Bright-field scanning TEM image of the resulting porous network of NCs. (B) Cycling of plasmonic absorption between dispersed (90 °C) and gelled (40 °C) NCs. (C) Chemically tunable gelation temperature by concentration of tetrabutylammonium chloride, monitored by *in situ* Fourier transform infrared spectroscopy (FTIR) spectroscopy of the plasmon resonance peak. Adapted with permission from ref 544. Copyright © 2022 The American Association for the Advancement of Science.

Different sizes and compositions (*i.e*., tin doping concentrations) of ITO NCs could be readily intermixed, while the gel structures observed by SAXS and their optical properties were highly reproducible even upon repeated thermal cycling.^547^

The versatility of NC gels to create porous architectures with high internal surface areas, to readily incorporate multiple components without constraints on size or composition, and to rapidly restructure and change their properties in response to selected stimuli is unmatched by other assembly strategies. In principle, equilibrium gels with spatially uniform, stable structures can be created by linking^550,551^ though this has yet to be demonstrated with NC building blocks. A wealth of opportunities remains to be explored in tuning their properties and realizing their potential for applications ranging from catalysis to electrochemical storage and conversion and optical switching.

### Photolithography/2D Patterning

NCs with colloidal stability provide an ideal platform for constructing NC-based devices via a solution process.^552–554^ Utilizing NCs enables the efficient and cost-effective fabrication of high-performance single devices.^555–557^ However, real-world applications necessitate the simultaneous integration of multiple components within complex device architectures.^558–560^ Therefore, there is an urgent need to develop nondestructive and precise patterning strategies tailored for colloidal NCs.^561^

Currently, various techniques are utilized for patterning NCs, including photolithography,^562^ transfer printing,^563^ inkjet printing,^564^ and doctor blading.^565^ Each method has undergone systematic optimization regarding resolution, throughput, fidelity, and cost per patterned element.^566^ While all patterning techniques have merits, photolithography is preferred for manufacturing highly complex structures. As a representative of parallel printing processes, photolithography is well established in terms of resolution, fidelity, and integration in manufacturing. In addition, its scalability and cost-effectiveness further contribute to its widespread adoption in the industry.^567^

Traditional photolithography processes rely heavily on photoresists, which require using polymers to create templates prior to NC patterning.^568–571^ This adds complexity to the process (Figure 18A). In addition, existing photopolymer lithography techniques struggle to meet the demands of constructing fine NC structures via the solution process. For example, polymers can swell in solvents, affecting pattern resolution, while the presence of organic molecules reduces the NC filling density within pixels.^572^ Additionally, capillary forces during solvent drying result in an uneven pattern layer thickness.^573,574^ More importantly, the introduction of various chemicals, such as developers and chemical etchants, during the patterning process inevitably affects the intrinsic physical and chemical properties of the NC film.^575^ It is, therefore, a significant challenge to overcome the limitations of polymer photoresists and achieve efficient patterning of NCs without compromising their inherent properties.

**Figure 18:**
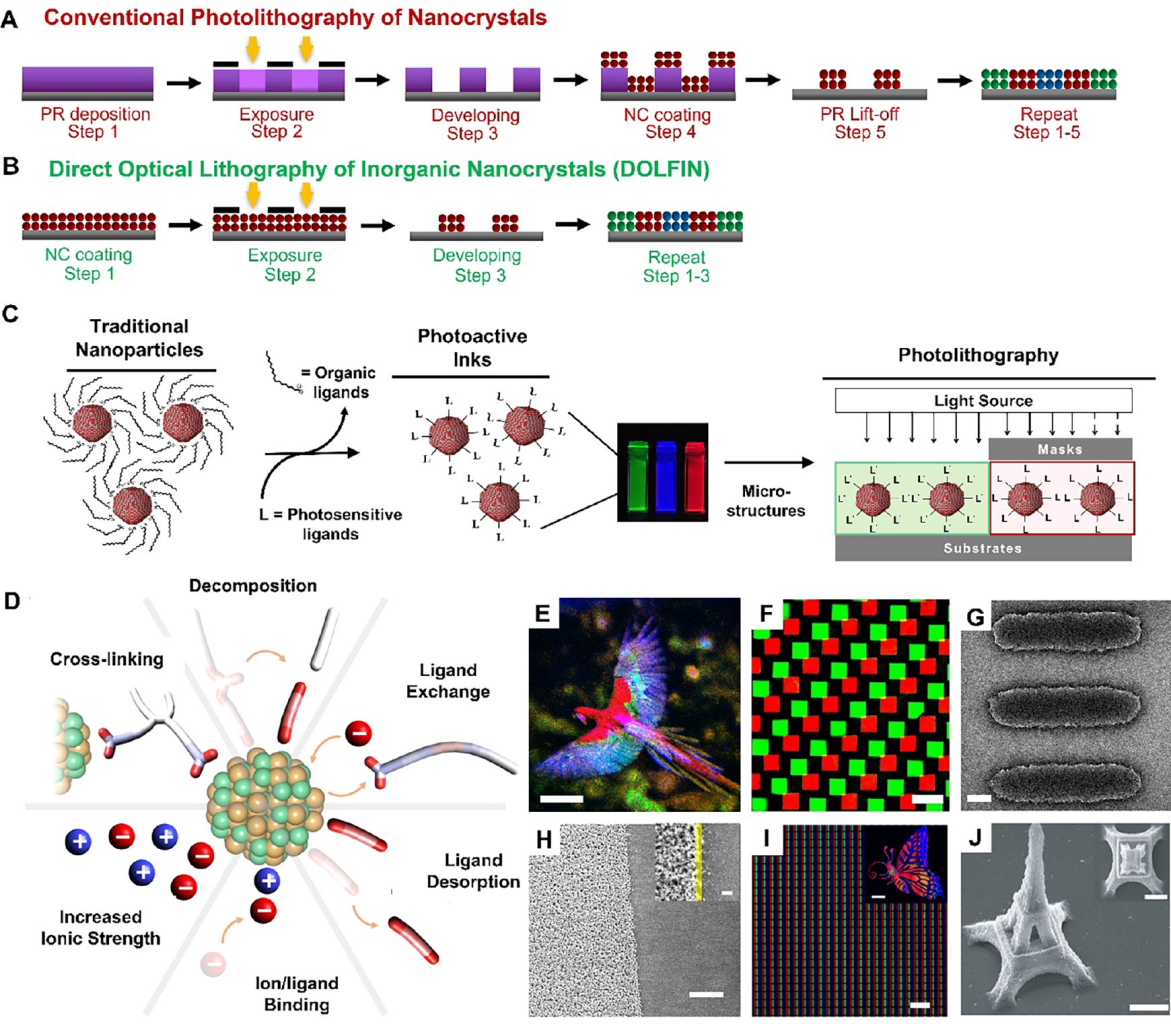
Patterning NCs through (A) a conventional photolithography process and (B) the direct optical lithography of inorganic NCs (DOLFIN). (C) Schematic diagram of the preparation of DOLFIN Inks and the exposure process. (D) Various types of chemical changes in photosensitive ink under irradiation. Reproduced from ref 582. Copyright © 2023 American Chemical Society. (E) Fluorescent multicolored image composed of RGB NC patterns fabricated by repeated DOLFIN processes. Reproduced with permission from ref 576. Copyright © 2017 The American Association for the Advancement of Science. (F) Red and green double color patterned film with squares of 250 μm. Reproduced with permission under CC BY 4.0 from ref 605. (Copyright © 2022 Zhang et al. open access). (G) SEM image of patterned “bare” NCs from photosensitive inks. Reproduced from ref 577. Copyright © 2019 American Chemical Society. (H) SEM image of the edge of the patterns. Inset: highlight of the boundary region. Reproduced with permission under CC BY 4.0 from ref 581. (Copyright © 2023 Xiao et al. open access). (I) Fluorescence image of RGB pixels obtained from PGMEA solvent. The inset shows a complex large pattern. Reproduced from ref 603. Copyright © 2025 American Chemical Society. (J) SEM images of a model of the Eiffel Tower constructed with 3D printing by a fs laser. The inset shows the top view. Reproduced with permission from ref 604. Copyright © 2023 The American Association for the Advancement of Science. Scale bars, 5 mm (E), 500 μm (F), 500 nm (G), 2 μm (H) and 5 μm (J).

Unlike traditional photolithography, the recently emerged direct optical lithography of functional inorganic nanomaterials (DOLFIN) represents a photoresist-free lithography process achieved by altering the stability of NCs through selective light exposure (Figure 18B).^576^ This method greatly simplifies the steps involved in NC patterning without modifying the existing lithography equipment. Typically, only three steps—NC coating, exposure, and development—are required to pattern a single type of NCs. Multilayer patterns can be obtained by repeating the three steps with a mask aligner system. In addition, since this technique eliminates the use of traditional polymers during the lithography process, it effectively avoids the aforementioned issues associated with photoresists, ensuring nondestructive and high-precision patterning of NCs.

To implement a DOLFIN, photosensitive inks composed of NCs and photochemically active molecules must first be prepared (Figure 18C). The design and selection criteria for photochemically active molecules are as follows: (i) they should not affect the colloidal stability of the NCs; (ii) under light exposure, they should undergo changes that induce variations in the stability of the NCs themselves; (iii) the resulting products after the changes should not affect the intrinsic properties of the NCs.

Based on these criteria, photochemically active molecules can be classified into three categories according to their interactions with NCs. One category is multifunctional ligand molecules, which not only act as ligands to provide colloidal stability to NCs but also exhibit photosensitive properties, undergoing chemical changes upon excitation by light of a specific photon energy. For example, in molecules such as thiazole, thiocarbamate, and xanthate, thiol groups serve as functional groups that attach to the surface of NCs. However, these molecules are unstable under light exposure, and the resulting small molecules can no longer ensure the stability of NCs in the corresponding solvents.^577^

Another category is additive molecules, which only serve as photosensitive elements in the photosensitive ink and do not interact with NCs. Representatively, photoacid generators release acidic protons under light exposure, changing the type of surface ligands on NCs and thus affecting their solubility.^576^ Molecules such as bis­(fluorophenyl azide) and bis-diazo change the stability of NCs by triggering cross-linking of the original surface ligands through free radicals generated in the presence of light.^578–580^ The third type of molecules exhibits no significant interaction with NCs before light irradiation. However, after irradiation, these molecules transform into ligands and regulate the solubility of NCs through interactions. In a typical example, Xiao and co-workers demonstrated the possibility of photoamine generators (PamGs) as photoactive molecules for NC patterning. The decomposition of butylamine-based PamGs releases primary amines, which bind to exposed surface cations of NCs, reduce the solubility of NCs in polar solvents, and passivate the defects created during the lithography process.^581^

Under light exposure, six types of chemical changes occur in photosensitive inks,^582^ including cross-linking,^583–586^ decomposition,^587–590^ ligand exchange,^174,591,592^ ligand desorption,^593,594^ ion/ligand binding,^577,581,595^ and increased ion strength (Figure 18D).^596^ All these chemical changes ultimately lead to variations in the intermolecular forces between NCs, resulting in changes in their solubility or dissolution rate, thereby achieving patterning.^597,598^

In terms of molecular design, the structure of the photosensitive ligands can be adjusted to ensure that the ligands used in NC inks have different absorption spectra covering different regions of the UV–vis spectrum. In this way, we can utilize a wide range of photon energies, including DUV (254 nm), near-UV (*e*.*g*., i-line, 365 nm), blue (*e.g*., h-line, 405 nm), and visible (450 nm), for direct patterning of NCs. In addition, the preparation of photosensitive inks is not limited by the type of NCs.^577^ Various NCs, such as metals, oxides, perovskites, and semiconductor QDs, can be transformed into photosensitive inks through ligand exchange or additive methods, thereby achieving precise patterning through DOLFIN technology.^576,599–601^ In particular, for luminescent NCs, which are considered the most promising luminescent materials for displays, the patterning layer can fully retain the photoluminescent properties. Moreover, interference is minimized in the sequential patterning of multiple layers (Figure 18E,F). Currently, using the DOLFIN process, uniform NC patterns with a 700 nm lateral size can be achieved with a resolution close to the limit of photomasks (Figure 18G)^577^ with the edges of the patterned regions being sharp and clean with roughness below 200 nm (Figure 18H).^581^ The photosensitive inks not only meet the need for high resolution with small features but also allow for large-area patterning.^602^ Recently, through molecular design, DOLFIN has been further advanced to enable efficient patterning using i-line and h-line light sources in industry-friendly solvents. (Figure 18I) This development enhances DOLFIN’s compatibility with mainstream industrial photolithography processes, paving the way for its widespread adoption as a universal additive manufacturing technology in real-world applications.^603^

More importantly, the NCs patterned by DOLFIN retain their intrinsic properties and can be further processed to control characteristics such as optical properties and porosity to meet practical application requirements.^581,596^ Additionally, photosensitive inks are suitable for various lithography methods. Through techniques such as direct laser writing, NCs can be assembled at the sub-100 nm scale.^577^ With femtosecond laser technology, 3D printing of NCs can be realized based on 2D assembly (Figure 18J).^604^ We believe that the emergence and development of DOLFIN technology provide a powerful approach for patterning NCs, which will be widely applied in quantum light-emitting diodes, photodetectors, and diffractive optical elements in the future.

### 3D Printing

Colloidal inorganic NCs have been powerful building blocks for making inexpensive and efficient 2D solid-state electronic and optoelectronic devices.^2,553,606^ However, integrating NC building blocks into 3D device platforms is still challenging.^607^ Relevant techniques, such as 3D printing, are thus critical for incorporating NCs in advanced integrated circuits, optics, 3D displays, and others.

Several 3D printing strategies have been developed for NCs during the past five years based on understanding several key concepts of colloidal NCs, including colloidal stability, interparticle interactions, and surface (photo)­chemistry. One straightforward approach is the nozzle-based extrusion of NC inks, which solidify into 3D objects. Early examples using this approach include the omnidirectional printing of Ag NCs to form microscale interconnects for flexible electronics.^608^ More recently, Kim and co-workers used nanoscale nozzles to form a femtoliter meniscus of QD inks and produced vertically freestanding nanopillars (Figure 19A).^609,610^ By adjusting the ink rheology with polystyrene additives, 3D nanopillars composed of red, green, and blue QDs can be sequentially printed with a lateral dimension of 620 nm and a pitch of 3 μm.^609,611^ However, this approach suffers from limited printing resolution (restricted by the nozzle sizes) and deficiency in forming complex 3D structures.

**Figure 19:**
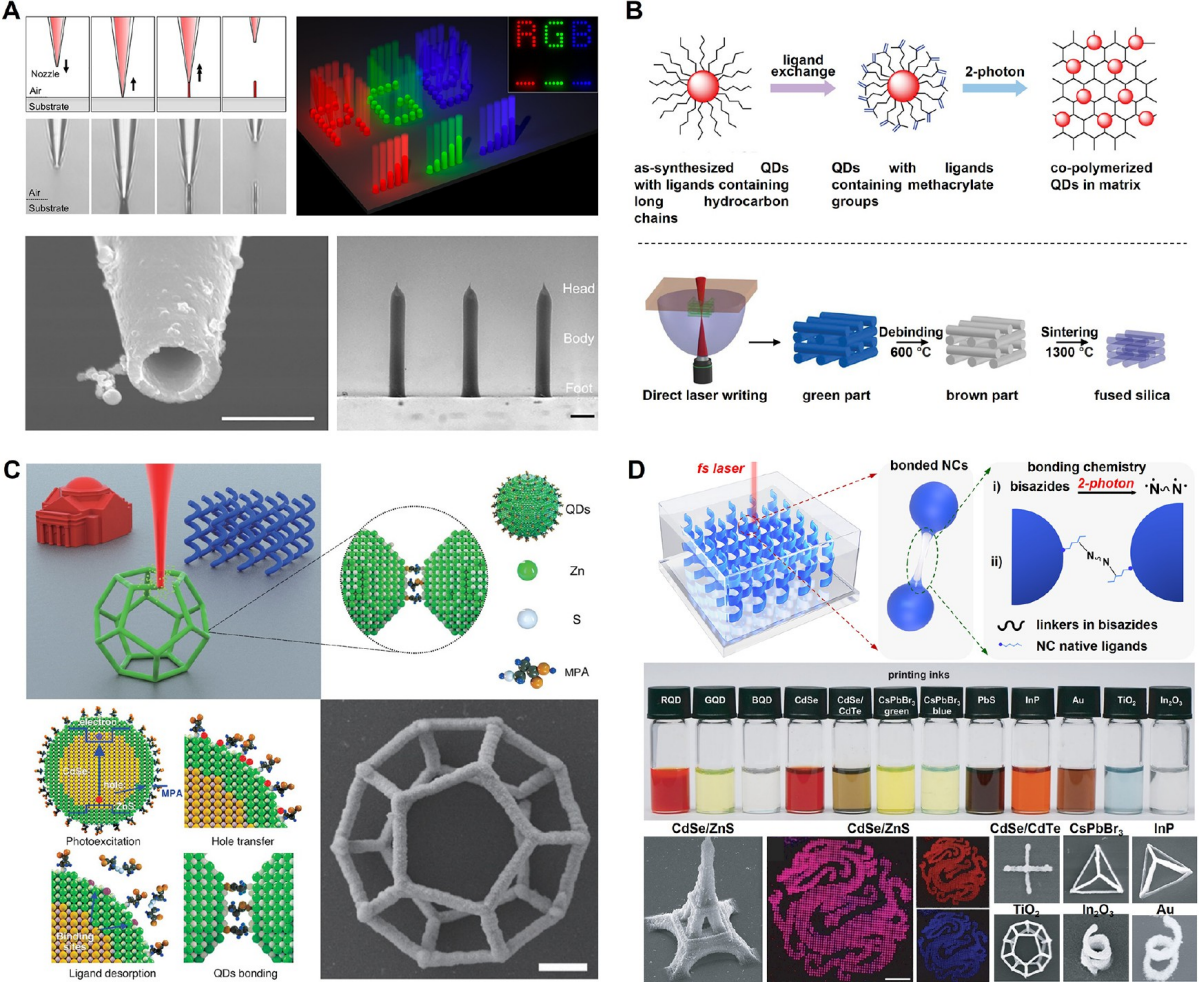
Representative strategies for 3D printing of NCs. (A) Nozzle-based printing of NC inks. The nanoscale nozzles form femtoliter meniscus of QD inks, which solidify into nanopillars illuminating in red, green, and blue. Reproduced from ref 609. Copyright © 2020 American Chemical Society. (B) Printing NCs with photocurable organic resins. NCs (*e*.*g*., silica or QDs shown here) with suitable surface ligands can be mixed with photocurable resins. The mixture can be 3D-printed via laser writing apparatus. Subsequent sintering at high temperatures is necessary to burn off the organic matrices but typically cause volume shrinkage. Adapted with permission under CC BY 4.0 from ref 616. (Copyright © 2023 Advanced Materials, Kirchner et al. open access); ref 631. (Copyright © 2021 Rapp et al. open access). (C, D) 3D printing of NCs via direct photochemical bonding. (C) Scheme of the mechanism of resist-free, direct 3D nanoprinting of CdSe/ZnS core/shell QDs via PEB and the printed dodecahedron of densely packed QDs. Reproduced from ref 618. Copyright © 2022 The American Association for the Advancement of Science. (D) Scheme of a general approach for direct 3D printing of inorganic nanomaterials (3D Pin) using bisazide-based linkers, the photograph of printable NC inks, and SEM images of printed complex 3D structures with various compositions. Reproduced from ref 604. Copyright © 2023 The American Association for the Advancement of Science.

Interparticle interactions originate from the relatively weak van der Waals forces between NCs (in the order of a few kJ/mol), which cannot provide sufficient mechanical strength to retain the 3D shapes. Printing intricate structures, especially those containing overhanging components, requires bonding between the building blocks—the atoms, molecules, or small pieces of materials.^604,612^ For instance, the formation of strong metal–metal bonds and covalent C–C bonds (bond energy ∼ 350 kJ/mol) sets the basis of the powder bed fusion printing of metals and the photopolymerization-based 3D printing of plastics, respectively. A commonly used strategy to increase the mechanical robustness of 3D-printed NCs is to mix NCs with printable organic resins (Figure 19B).^613,614^ Ligands containing similar molecular structures or reactive groups with the resins are frequently used to improve the compatibility of inorganic NCs and organic resins (Figure 19B, top right).^615,616^ During printing, the organic components are photochemically bonded, producing mechanically stable matrices to host NCs. As this approach is fully compatible with commercial two-photon polymerization printing techniques, arbitrary 3D structures can be obtained with nanoscale resolution (sub-100 nm).^613^ However, the printed inorganic–organic hybrids have an inorganic mass fraction far below 50%. To increase the material purity, the organic components need to be “burned-off” at high temperatures (from 500 to over 1000 °C, Figure 19B).^615,617^ Such harsh postprinting procedures can lead to significant structural shrinkage and are incompatible with NCs of many compositions, such as semiconductor QDs.^607,618^ Therefore, this strategy is mostly applied to metal oxide NCs, whose sizes and chemical integrity are less sensitive to sintering and impurities, and shows promise in printing fused silica glass and mechanical metamaterials.^615,617,619,620^ Other than photocurable resins, glass^621^ and 3D patternable hydrogels^622^ can also serve as matrices for positioning inorganic NCs in 3D. However, the presence of all these matrices reduces the material purity and impairs the properties of NCs, especially for semiconductor NCs.

3D printing of NCs with high material purity and versatility requires direct chemical bonding between NCs. The surface ligands, which are critical to NC growth, colloidal stability, and inter-NC communication,^415^ play a key role in the bonding chemistry. Liu et al. reported a resin-free, direct 3D printing method for QDs.^618^ This method, termed photoexcitation-induced chemical bonding (PEB), relies on a series of photoexcited chemical transformations of ligands on the QD surface, the subsequent interparticle bonding reactions, and the solidification of QDs into 3D objects from their colloidal inks (Figure 19C). The inks used for printing are aqueous solutions of CdSe/ZnS QDs capped with bidentate, 3-mercaptopropionic acid (MPA) ligands. The thiol terminals of MPA ligands are bound to the NC surface, leaving the carboxylate terminals extended in the solvent. When triggered by the 780 nm fs laser, the two-photon excitation of CdSe/ZnS QDs generates energetic holes that can induce the oxidation and detachment of MPA ligands. The resultant uncoordinated metal sites then bond with the carboxylate groups of MPA ligands on adjacent QDs. QDs bridged by the bidentate ligands ultimately solidify into 3D objects whose shapes follow the laser paths as the reactions only occur at the focal point. Using this method (PEB), arbitrary 3D structures composed of densely packed QDs are printed, and the finest feature is below 80 nm. However, PEB relies on the effective photoexcitation of QDs, proper energy alignment between QDs and ligands, and the capability of binding/detachment of the bidentate ligands. These requirements restrict PEB to specific combinations of semiconductor QDs and surface ligands.

More recently, Li et al. developed a general photochemical bonding strategy (coined as 3D Pin) for a broad range of colloidal NCs, including semiconductors, metal oxides, and metals.^604^ The material versatility can be traced to the nonspecific photochemical bonding between the native ligands on the NC surface by the bisazide linkers added to NC inks (Figure 19D). Through one-photon or two-photon processes (*e*.*g*., 780 nm irradiation), the bisazide linkers can photogenerate reactive nitrene intermediates at both ends and bridge the ligands on adjacent NCs via C–H insertion, forming mechanically stable 3D objects of cross-linked NCs. The photochemical bonding starts from the light absorption of bisazide cross-linkers and thus does not rely on the optical properties of NC building blocks. The bonding reaction requires only C–H groups, which are abundant in typical NC ligands. The combined features render 3D Pin effective in printing over 10 semiconductors (II–VI, III–V, IV–VI, and lead halide perovskite-based NCs), metals (Au), metal oxides (In_2_O_3_, TiO_2_), and their mixtures (Figure 19D). Using a 780 nm fs laser printing setup, 3D Pin can construct arbitrary 3D structures of NC solids with porosity smaller than 5%. Another advantage of 3D Pin is the minimal amounts of organic components in the printing inks, which converts to a high inorganic mass fraction (∼90%) in the final 3D structures. The amount of organic components can be further lowered by using postprinting chemical^623^ or thermal treatments, implicating the building of all-inorganic 3D structures and functional devices.

3D printed structures of NCs are promising in various applications. For example, 3D printed luminescent QDs or perovskite NCs by extrusion,^609,610^ matrices,^616,621,622,624^ or direct photochemical bonding^604,618^ strategies can preserve their intrinsic size- and composition-dependent photoluminescent properties. Related applications include 3D holographic displays, multilevel anticounterfeiting, and optical storage. Beyond this, the geometric designs at the subwavelength level can introduce structure-dictated optical properties not available in the NC building blocks, as exemplified by optical metamaterials.^625^ Handed helices composed of CdSe/ZnS semiconductor QDs show a broadband chiroptical absorption from 400 to 1000 nm.^604^ The peak anisotropic factor is 0.24, or 20 times higher than those exhibited in self-assembled chiral helices of CdTe QDs. Compared to the optical absorption or emission, achieving excellent electronic properties in 3D printed NC structures, except for those composed of metals, is more challenging. The presence of organic components, even in small fractions, can hamper the interparticle charge transport.^626^ It is thus desirable to develop direct photochemical bonding strategies to 3D-print all-inorganic materials, which involve inorganic linkers between adjacent NCs. Very recently, Son and co-workers reported an extrusion-based 3D microprinting method to solidify inorganically capped NCs via metallic ion bonding and switched solvent polarity.^627^ Although the printing resolution and structural complexity need to be improved, this method may potentially print structures with good charge transport. Additionally, the mechanical properties of 3D printed structures of NCs remain largely unexplored.^628,629^ Recent studies in mechanical engineering have suggested that the sizes of building blocks and elementary units are critical for the mechanical properties of the constructed structures.^630^ Recent work also showed that printed CdSe/ZnS QD based pillars exhibited both high compressive strength (∼1 GPa) and large fracture strain (∼55%), which are hard to coexist in traditional materials.^604^ 3D printing of NCs with different compositions, sizes, and surface chemistries may offer additional degree of freedom in designing mechanical metamaterials with unprecedented properties.

To sum up, 3D printing of NCs is still in its infancy. Like 3D printing techniques for other functional materials,^553^ the goal of NC 3D printing is to make structures not only geometrically but also functionally complex and provide a disruptive technology for 3D-printing fully functional devices with various components. To this end, from the chemistry perspective, developing 3D printing chemistry with better control of the material’s purity and complexity and understanding how the printing chemistry affects the properties of obtained structures would be important. Existing knowledge of the surface chemistry (organic,^415^ inorganic,^2,554^ and photochemistry^576,584,602^ ) of NCs may provide useful guidelines. From the engineering perspective, the printing chemistry innovations (photosensitivity, cross-linking efficiency, new printing mechanisms, *etc*.) and apparatus optimizations are needed to improve the printing resolution, speed, and related parameters.

## Applications

### IR Sensing and Emitting Devices

As NCs have matured, their potential applications have expanded beyond narrow PL capabilities. However, this progress has introduced new challenges, particularly in reconciling optical features with charge conduction. The initial focus was on solar cells for light detection. Early interest stemmed from the ability of lead sulfide to absorb the NIR part of the solar spectrum^632,633^ and the potential use of multiexciton generation^634–636^ to overcome the Shockley–Queisser limitation in power conversion efficiency.

While the excitement around NC-based solar cells has waned with the success of perovskites, which, due to their defect-tolerant electronic structure, have achieved higher open-circuit voltage,^637,638^ neither perovskite nor organic electronic polymers have yet demonstrated IR photoconduction beyond 1 μm. Significant efforts have been invested in colloidal materials to expand their spectral ranges (SWIR: 1–2.5 μm, MWIR: 3–5 μm,^639,640^ LWIR: 8–12 μm,^557,641^ and even THz)^642–646^ where conventional semiconductors (In_1–x_Ga_*x*_As, InSb, Hg_1–x_Cd_*x*_Te) have dominated for decades. The cost-prohibitive nature of IR sensors, due to epitaxial growth requirements and the need for high-temperature manufacturing, poses a challenge for widespread adoption in mass-market applications.

The advent of alternative technologies^647,648^ for IR sensing extends beyond proving the concept of IR photoconduction and the potential promise of reduced fabrication costs. Emerging technologies must also offer additional functionalities and performance improvements to provide advantages over existing options. Colloidal QDs for IR sensing offer several advantages compared to their thin-film counterparts. The colloidal growth process eliminates epitaxial constraints, providing several benefits, including (*i*) reduced toxicity, as the substrate (Cd_1–*x*_Zn_*x*_Te, InSb, GaAs) is often the primary source of heavy metals; and (*ii*) decreased energy costs, as colloidal growth occurs at much lower temperatures (in the range of 50–250 °C) compared to the typical temperatures for epitaxy (500–800 °C).

Since the substrate is removed, it alleviates optical constraints associated with it. For illustration, consider In_1–*x*_Ga_*x*_As semiconductor growth on an InP substrate that absorbs below 900 nm, rendering the active layer artificially blind in the visible spectrum. Lastly, colloidal growth simplifies the coupling to the read-out integrated circuit (ROIC). Typically relying on complementary metal-oxide-semiconductor (CMOS) (Si-based) technology, the coupling between the light-absorbing layer and the ROIC usually involves small metallic bumps (often made of In), a process with limited yield that also restricts pixel pitch reduction. With NCs that can be directly deposited onto the ROIC surface, pixel sizes below 2 μm have been demonstrated,^649,650^ achieving diffraction-limited operation and leading to improved image quality.

#### Transitioning NCs from Single Pixel to Camera

While demonstrations of IR photoconduction using NC films were reported 20 years ago, the transition to imagers is more recent. Initial efforts were concentrated on two materials, PbS^649,651–653^ and HgTe,^107,654–657^ which are indeed the most mature. These materials not only need to exhibit some degree of air stability and intrinsic photoconduction but also must be compatible with relatively large-scale synthesis for imager applications. Initially focused on the NIR spectrum (around 940 nm, facilitating the switch from solar cells), efforts are now directed toward face recognition in smartphones, with a current trend shifting toward longer wavelengths, typically around 1400 nm, corresponding to the water absorption band. In this spectral range, In_1–*x*_Ga_*x*_As is the main existing technology with a cutoff wavelength of ≈1.7 μm when lattice-matched on an InP substrate. However, achieving spectral tunability for In_1–*x*_Ga_*x*_As requires compromising epitaxial matching on the InP substrate, making growth more challenging and expensive.

In contrast, NCs can be easily tuned by a simple change in size while offering high material maturity at least up to 5 μm.^658,659^ PbS cameras up to 2 μm (with several megapixel sensors^660^) have been demonstrated, with efforts focused on photovoltaic operation. For HgTe, both SWIR and MWIR cameras have been demonstrated, operating in various modes, including photovoltaic,^654^ photoconductive,^107,656,661^ and phototransistor^655^ modes. The photoconductive mode, in particular, is interesting for designing cost-effective devices since they can rely on a single fabrication step, which also facilitates obtaining homogeneous large-scale films.

However, the transfer onto the ROIC for commercialization raised additional requirements. One of them relates to flatness, as hopping conduction, due to its inherent short diffusion length, allows for thin films (100–1000 nm) only to conduct charge efficiently. Achieving high-quality, homogeneous films over the ≈1 cm surface of the ROIC requires that the ROIC’s roughness and flatness are far below the targeted device thickness, as shown in Figure 20B,C. This necessitates additional steps compared to In_1–*x*_Ga_*x*_As to flatten the top dielectric and optimize contact thickness. NC deposition (Figure 20D,E) can be conducted either at the wafer scale, with demonstrations up to 12 in. by STMicroelectronics,^649^ or at the die level. Several groups have reported impressive high-quality images, and such cameras are now commercially available.

**Figure 20:**
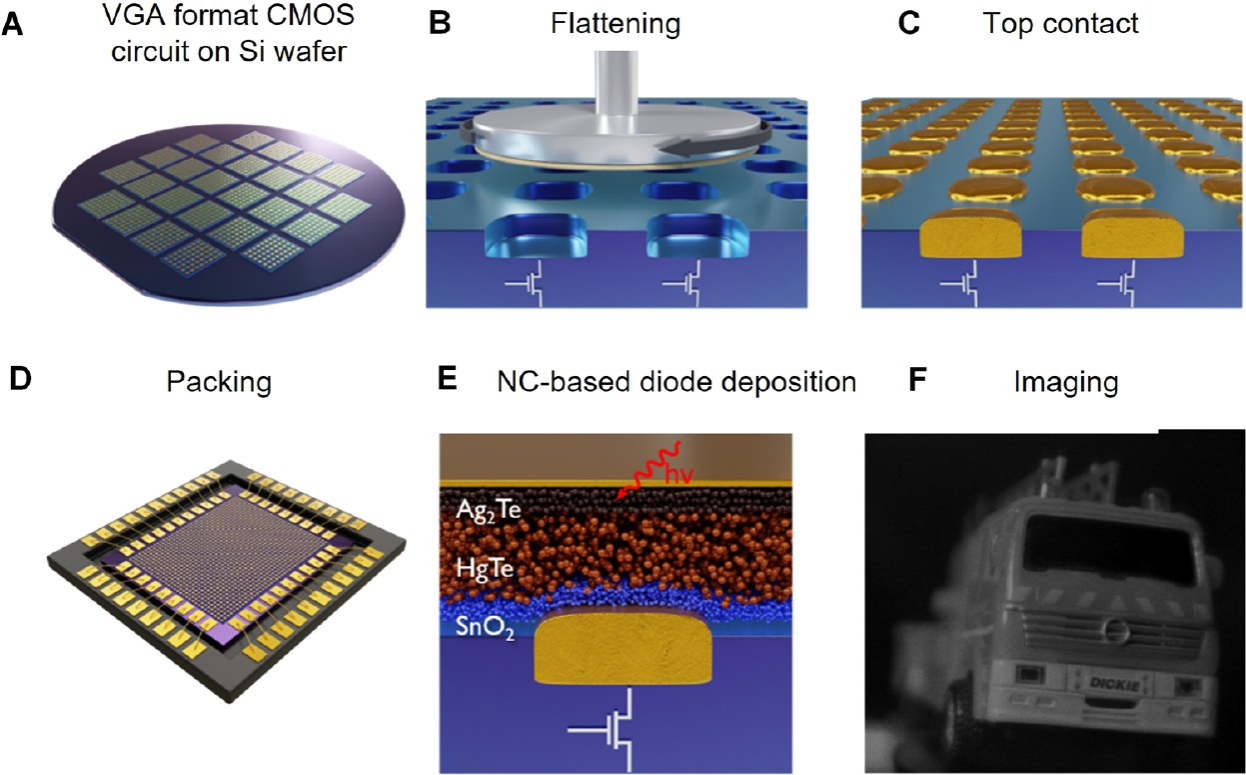
(A) Step one is dedicated to the *video graphics array* format array that is fabricated on an 8-in. wafer. (B) This wafer is then polished to obtain a flat surface. (C) Electrode contacts are grown with a top gold plating to minimize amalgam formation with the HgTe NCs deposited later. (D) The wafer is then sliced and packaged. (E) The diode stack is deposited by spin coating; the top Au electrode is 20 μm thick and thus is semitransparent. Parts (A–E) are adapted with permission from ref 654. Copyright © 2023 Alchaar et al. (F) Image acquired with an HgTe NCs-based sensor, operated in photoconductive mode.

It would be a mistake to assume that achieving an image means only technological developments such as packaging remain. Better synthesis methods are still necessary: PbS faces issues with oxidation, and HgTe struggles with poor thermal stability^662^ (tending to sinter at the operational temperature of the ROIC due to the Joule effect) and has a propensity to form amalgams^663^ with certain metals, both of which contain heavy metals. Recent efforts have focused on the emergence of III–V NCs (InAs^664–666^ and more recently InSb)^667^ or silver chalcogenides,^668–671^ but these developments are still at the material scale.

At the single-pixel level, considerable success has been achieved by coupling the absorbing layer to photonic structures to enhance absorption,^672–675^ shape the spectral response,^676,677^ or even achieve an actively tunable response.^678–680^ Transferring such concepts to the ROIC level remains technologically challenging,^654,657^ not only due to the processes involved but also because resonator concepts need to remain valid at the pixel size, which tends to be only a few wavelengths wide. To date, only the vertical Fabry–Perot concept has been implemented at the image sensor level, while concepts based on gratings are limited to single-pixel devices. Thus, the combination of NCs and CMOS is still in the early stages of synergic interaction. This will require the development of new *operando* characterization tools.^661,681^

#### HgTe-Based Plasmonic Device Architectures

Despite recent achievements in the morphology control of mercury chalcogenide QDs,^102^ enhancing their emission in the IR spectral range is still a challenge because radiative recombination efficiency drops dramatically for longer wavelengths according to Fermi’s golden rule. To address this issue, researchers focused on engineering of the device architecture by coupling of HgTe QDs with plasmonic structures.^106,108,672^ The choice of the plasmonic structures appears to be rather complicated since it has to match the excitation and emission bands of the QDs.^682^ Moreover, alongside an enhanced excitation efficiency, several competitive processes may occur, including the Purcell effect and plasmon-assisted energy dissipation. The incorporation of periodic plasmonic structures as part of the vertical diode stack became a rather common approach to enhance absorption and emission across the NIR to MWIR ranges, and gold plasmonic gratings are widely used.^682^ Modifying the plasmonic grating geometry allows us to tune the dispersion of the plasmons, therefore enhancing the emission and also favoring directional light.^99,683^ Recently, the integration of metallic gratings as both the electrodes and multiresonators in the HgTe QDs-based photodetector has been reported.^673,684^ These gratings were shown not only to enhance strong broadband absorption of HgTe QDs but to reduce IR light reflection losses due to the absence of a conventional ITO electrode layer. Additionally, combining the gold gratings of different periods within a single device allowed for selective response through SWIR to MWIR ranges.

Although relatively low-cost, easily fabricable, and providing flexible tunability, the metal grating can ensure only up to a 2-fold increase in IR performance of HgTe QDs. To achieve higher efficiency, more sophisticated structures are to be developed. As recently demonstrated by Sergeev et al.,^685,686^ periodically arranged metasurfaces (plasmonic nanoantennas) supporting bound states in the continuum (BIC) modes can significantly enhance the spontaneous NIR emission rate of HgTe QDs (Figure 21A). More than an order of magnitude stronger PL signal was detected from a ML of HgTe QDs deposited on a plasmonic metasurface with a dielectric spacer between them (Figure 21B). Given that the BIC mode spectrally matched the emission maximum, the observed PL enhancement and its spectral shaping were assigned to the Purcell effect. Moreover, it was found that the QDs’ emission along the normal to the metasurface was suppressed, and the highest NIR PL intensity was detected at 20° (Figure 21C). This effect occurred due to the nature of the BIC mode, as confirmed by numerical simulations.

**Figure 21:**
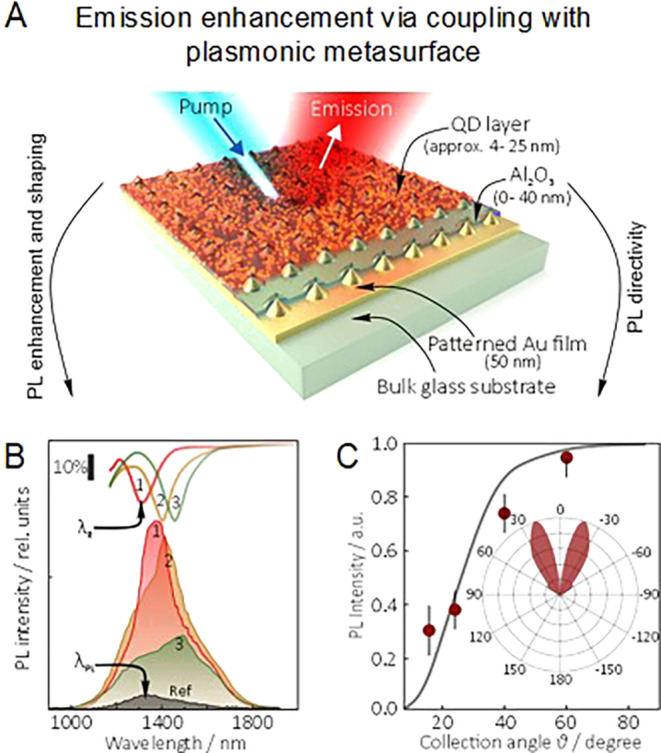
(A) Sketch of the HgTe QDs/plasmonic metasurface (ordered array of nanoantennas). (B) HgTe QDs’ PL enhancement and (C) directionality achieved through interaction with a BIC-supporting plasmonic metasurface illustrated in (A). Figure reproduced with permission from ref 686. Copyright © 2023 Wiley-VCH GmbH.

Overall, the coupling of the HgTe QDs with plasmonic metasurfaces is a promising research avenue, offering exciting opportunities in terms of both the fundamental science and device engineering. The number of studies on the above-discussed use of metal gratings, metasurfaces, or such nontrivial approaches as an optical equivalent of acoustic resonators^687^ is growing rapidly and can advance subsequent development of next-generation IR-emitting and sensing devices.

### Lasers and Laser Diodes

Colloidal QDs are attractive materials for implementing wavelength-selectable light-amplification and lasing devices.^316,688–693^ In addition to being compatible with inexpensive and readily scalable chemical techniques, QDs offer multiple advantages derived from a zero-dimensional (0D) character of their electronic states. These include a size-tunable emission wavelength, low optical-gain thresholds near one exciton-per-dot on average, and high temperature stability of lasing characteristics stemming from a wide separation between QD’s discrete energy levels.^689,693^

It has been more than three decades since the first demonstration of QD lasing.^694^ These early studies employed CdSe NCs embedded in a glass matrix—the samples akin to standard colored glass filters.^695^ Following this discovery, it took three years to realize lasing with epitaxial QDs^696^ and six more years to demonstrate the effect of amplified spontaneous emission (ASE)—a precursor of lasing—with colloidal QDs.^688^

The most recent advance—the realization of ASE with electrically stimulated QDs^697^—has brought the field of colloidal QD lasing very close to its primary objective—the demonstration of an electrically pumped laser oscillator or a laser diode. If realized, such devices would open the door to a new laser-diode technology that is based on highly flexible solution-processable colloidal nanomaterials rather than traditional epitaxially grown III–V semiconductors. This would help resolve the challenge of integration of photonic and electronic circuits and, in particular, allow for facile preparation of optical amplifiers and lasers directly on top of a Si wafer. Implementing such on-chip optical-gain devices would foster a further increase in the complexity of integrated CMOS circuits, enhance scalability in traditional and quantum photonics, and push sensitivity limits in on-chip diagnostics.

A primary difficulty in realizing technologically viable colloidal QD lasing devices has been the extremely fast Auger recombination of optical-gain-active multicarrier states.^688,689,698^ In a QD, ‘light-emitting’ band-edge electron and hole energy levels are at least 2-fold degenerate. Hence, a single electron–hole pair (a single exciton) does not generate optical gain because stimulated emission by a conduction-band electron is compensated by absorption arising from an electron remaining in the valence band. This implies that the realization of optical gain requires a higher-order, multicarrier state such as a charged exciton (a 3-carrier state or a trion) or a biexciton (a four-carrier state). In fact, the biexciton is the most common optical-gain state that can be generated using, for example, high-intensity optical excitation of charge-neutral QDs.

While the biexciton does produce stimulated emission needed for light amplification, it can also decay via an Auger process during which an electron–hole recombination energy is transferred via Coulomb interactions to a third carrier residing in the same dot (Figure 22A). Auger recombination directly competes with stimulated emission and thereby impedes laser action.

**Figure 22:**
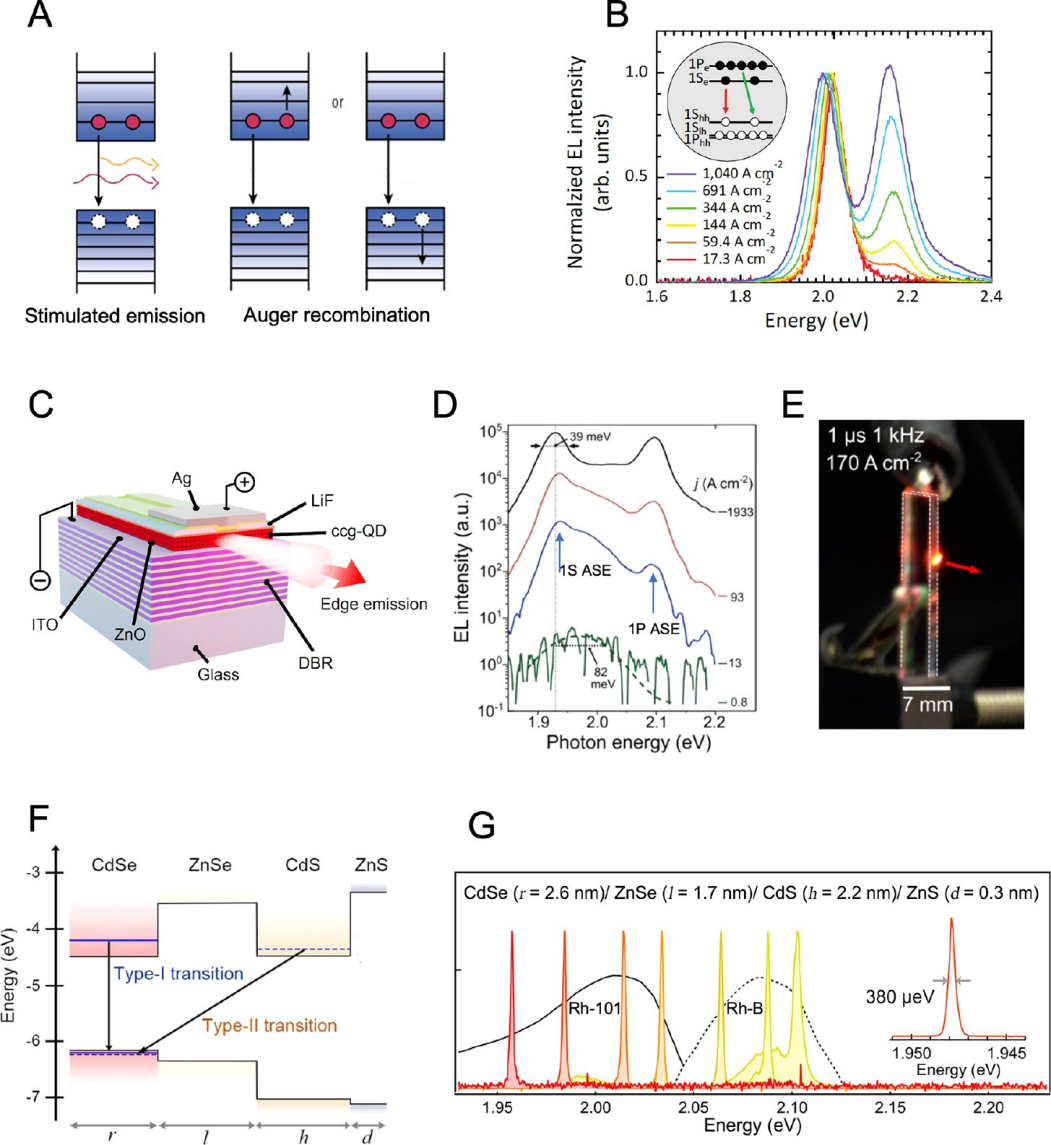
(A) In charge-neutral QDs, light amplification arises from stimulated emission by biexcitons (left). This process competes with biexciton decay via nonradiative Auger recombination (right). During Auger recombination, the electron–hole recombination energy is transferred via Coulomb interaction to another electron or hole within the same dot. (B) Normalized EL spectra of a current-focusing LED with a charge injection area of 0.015 mm^2^ as a function of *j* for excitation with 1 μs, 100 Hz square-shaped voltage pulses. Adapted with from ref 704. (Copyright © 2021 Springer Nature Limited open access). Emission peaks at 2.03 and 2.16 eV correspond to the 1S and 1P transitions, respectively (indicated by red and green arrows in the inset). The EL spectra are normalized to match the 1S peak amplitude. The recorded spectra show a gradual increase in the relative intensity of the 1P band versus the 1S feature with increasing *j*. This indicates the increasing filling of the 1S level, followed by the filling of the 1P state (inset). (C) An ASE-type LED that features a BRW formed by an underlying DBR composed of 10 Nb_2_O_5_/SiO_2_ bilayers, and a top silver electrode. Ccg-QD denotes a compact continuously graded quantum dot. (D) Current-density-dependent EL spectra of the BRW device exhibit the transition from broad-band 1S spontaneous emission (green line) to 1S and 1P ASE (blue, red, and black lines). The device was excited using 1 μs, 1 kHz voltage pulses. (E) The BRW device exhibits bright edge-emitted ASE clearly visible in daylight. The instantaneous emitted power reaches ∼2 kW cm^–2^ at *j* of ∼2 kA cm^–2^. Panels (C), (D), and (E) adapted with permission under CC BY 4.0 from ref 697. (Copyright © 2023 Ahn et al. open access). (F) Radial profiles of electron and hole confinement potentials in a type-(I+II) CdSe/ZnSe/CdS/ZnS QD. Here *r, l, h*, and *d* denote the radius of the CdSe core, the thickness of the ZnSe barrier, the CdS interlayer thickness, and the thickness of the outer ZnS shell, respectively. (G) Spectrally tunable lasing spectra obtained with the type-(I+II) QDs whose dimensions are indicated in the figure. The lasing line can be continuously tuned from 1.96 to 2.10 eV (590 to 634 nm) by varying a resonant wavelength of a Littrow cavity. As illustrated in the inset, the observed line width is 380 μeV (=1.2 Å). For comparison, the spectra of lasing efficiencies of traditional Rhodamine dyes (Rh-101 and Rh–B), are shown by gray shading. Panels (F) and (G) adapted with permission from ref 710. Copyright © 2024 Hahm et al.

In conventional (nonengineered) colloidal QDs, Auger recombination is characterized by very short lifetimes (tens to hundreds of picoseconds) that rapidly decrease with decreasing QD size following a well-documented volume scaling or “V-scaling”.^689,698,699^ The development of approaches for controlling Auger decay has been an essential part of the QD lasing research.^689,693^ One such approach entails the incorporation of compositional gradients into a QD interior for realizing a slowly varying (“smooth”) carrier confinement potential, which suppresses Auger decay by reducing overlap of the ground and excited states of the energy-accepting carrier.^700,701^

Recently, this approach was successfully implemented with so-called continuously graded QDs that comprised a CdSe core enclosed into a Cd_*x*_Zn_1–*x*_Se shell wherein *x* varied from 0 to 1 in a radial direction.^702,703^ For improved stability, this core/shell structure was overcoated with a layer of ZnSe_1–*y*_S_*y*_ followed by a final ZnS shell. As a result of compositional grading, the biexciton Auger lifetime was extended to 2.4 ns, which yielded a biexciton PL quantum efficiency of 45%.^702^ For comparison, in standard (nongraded) CdSe QDs with similar confinement energy, the biexciton Auger lifetime is only 30 ps, and the corresponding biexciton PL QY is less than 1%.

The invention of continuously graded QDs instigated several important advances in the QD lasing field, including the realization of optical gain in electrically pumped devices, the development of lasers operating in a subsingle-exciton regime,^703^ and the recent demonstration of electrically excited ASE.^697^ The latter work provided important proof of the feasibility of colloidal QD laser diodes (QLDs).

Besides fast Auger recombination, the realization of a QLD is complicated by additional problems specific to electroluminescent (EL) devices.^704–706^ These include the poor stability of colloidal QD solids at high current densities (*j*) required for attaining an optical-gain regime and large optical losses in various charge-conducting layers that compete with optical gain generated in a thin EL-active QD region.

The challenge of insufficient stability of high-*j* devices has been recently resolved by incorporating “current focusing” elements into a standard LED architecture to reduce the size of a charge-injection area.^702,705^ This allows one to reduce device overheating (a primary degradation mechanism) by reducing the amount of generated heat and simultaneously improving heat exchange with an environment. A further suppression of overheating is possible by using not direct-current but pulsed excitation.^705^ This helps reduce heat accumulation due to periodic interjection of short heating cycles with long cooling periods. As a result, the device overheating is reduced by a factor of about τ_p_/τ_T_, where τ_p_ is the pulse duration and τ_T_ = *C*/*K* is a characteristic heat dissipation time (*C* is the heat capacitance of the active device volume and *K* is the heat exchange constant).

The ideas of “current focusing” were implemented in ref 705 by inserting in an LED device stack a LiF insulating layer with a narrow (300 μm) slit and an orthogonal electrode (anode) shaped as a narrow 50-μm-wide strip. This limited the injection area to 300 × 50 μm^2^ or just 0.015 mm^2^. By further using excitation with short 1-μs pulses separated by 10 ms periods, the researchers were able to push *j* to unprecedented levels of more than 1000 A cm^2^, which was almost 3 orders of magnitude higher than maximal current densities realized in standard LEDs.

Extremely high *j* obtained with devices of ref 705 led to a highly unusual EL regime when the intensity of the above-band-edge emission originating from the 1P electrons (1P band) was greater than that of the band-edge 1S feature observed for standard LEDs (Figure 22B). This indicated the realization of high per-dot excitonic occupancies of ∼8 excitons per QD on average, which were sufficient to achieve population inversion (that is, optical gain) for both the 1S and the 1P transitions.

While devices of ref 705 attained the regime of QD population inversion, they did not exhibit ASE with either electrical or optical pumping. This suggested that optical losses arising from non-QD device components overwhelmed optical gain generated in the QD layer. The problem of excessive optical losses was tackled in ref 707 by redesigning the LED device stack. In particular, Ahn et al. replaced an optically lossy MoO_*x*_ hole injection layer normally used in QD LEDs with an organic hole injection layer. They further replaced standard ITO as a cathode material with less optically lossy low-index ITO made by mixing ITO with SiO_2_. Importantly, the redesigned devices preserved good electrical characteristics, which allowed for attaining *j* of up to 560 A cm^–2^. This was sufficient to achieve full inversion of the QD band-edge transition, resulting in strong optical gain.

Due to reduced optical losses, the devices of ref 707 exhibited net-positive optical gain when cooled down to a liquid-nitrogen temperature. This enabled the researchers to achieve optically excited ASE in cavity-free devices, and lasing (laser oscillations) in devices supplemented by a distributed feedback cavity integrated into the bottom low-index-ITO electrode. However, none of these effects was present at room temperature or under electrical pumping (either at room or liquid-nitrogen temperature), which was tentatively attributed to thermally induced optical losses. This indicated that further advancements in device architecture were necessary to achieve a better optical gain/loss balance.

This task was accomplished in ref 697, which employed a Bragg reflection waveguide (BRW) to realize a more favorable optical-field distribution inside the device. To create a BRW, Ahn et al. assembled their devices on top of a distributed Bragg reflector (DBR), which formed a transverse cavity terminated by a silver (Ag) anode acting as the second reflector (Figure 22C). By adjusting the DBR parameters, the researchers were able to shape an optical field profile in such a way as to increase a field intensity in the QD active region and simultaneously decrease it in the optically lossy charge transporting layer. As a result, the BRW devices exhibited a large net-positive, room-temperature optical gain of ∼50 cm^–1^, as was inferred from measurements with optical excitation.

Importantly, these devices also displayed all signatures of ASE, then excited by short (1 μs) electrical pulses (Figure 22D). At low current densities, the device radiated weak spontaneous emission at 1.98 eV with a large line width of 82 meV. When *j* exceeded 13 A cm^–2^, two narrower (∼40 meV) peaks emerged in the EL spectrum. Their spectral positions (1.93 and 2.11 eV) were consistent with those of 1S and 1P ASE bands observed for optically excited QD films. Further, a *j*-dependence of EL spectra revealed a distinctive superlinear EL intensity growth above threshold current density *j*_th_ = 13 A cm^–2^, accompanied by the narrowing of the band-edge EL feature. Despite the small size of the emitting spot and a small duty cycle of 0.1%, the BRW devices produce bright edge emission visible in daylight (Figure 22E). As was assessed by a standard laser power meter, the instantaneous edge-emitted power reached high values of ∼2 kW cm^–2^, which further confirmed the realization of the ASE regime.

The studies of ref 697 provided strong evidence of feasibility of electrically driven QD laser oscillators or QLDs. Such devices can be realized by, for example, supplementing BRW-type devices with a distributed feedback grating, as in references.^706,707^ Another approach is by cleaving device edges to create a Fabry-P*é*rot cavity. This can be accomplished by employing high-precision cutting techniques such as ion milling.

A further important direction in the area of QLDs is the realization of NIR devices that would be especially useful in on-chip Si photonics and electronics, telecommunications, and sensing technologies. There are a number of promising results with optically excited NIR lasing devices employing PbS QDs.^708,709^ The next challenge in this area is the realization of optical gain and ASE with electrical excitation and, then, true laser action.

Overall, the colloidal QD field has reached a maturity suitable for real-world applications. In particular, ASE-type QD LEDs hold promise for display and projector technologies, where their highly directional, high-brightness output could offer significant advantages.

Another promising class of devices ready for real-world applications is broadly tunable liquid-state QD lasers, as recently documented in ref 710. These lasers utilize so-called type-(I+II) QDs, which feature near-resonant type-I and type-II transitions (Figure 22F). Though precisely controlled coupling between the two transitions, these QDs support “hybrid” direct/indirect biexcitons with slow, charged-exciton-like Auger dynamics. As a result, type-(I+II) QDs exhibit long optical gain lifetimes (up to ∼3 ns), enabling lasing even in low-concentration solution samples.

The demonstrated devices exhibited dye-laser-like characteristics but with several notable advantages. In particular, they featured an extended optical gain bandwidth, enabling wide-range spectral tunability of the laser line from a single QD sample (Figure 22G). Additionally, they demonstrated excellent operational stability even without the need for circulating the QD solution, whereas dye lasers rely on continuous circulation for stable operation. Elimination of circulation is an attractive feature of liquid-state QD lasers as it significantly simplifies their design, reduces costs, and enhances their suitability for miniaturization and integration with other devices.

### Manipulating Hot Carriers via Ultrafast Spin-Exchange (SE) Interactions

Hot, nonequilibrated electrons offer immense potential for advanced photoconversion and photochemistry. Beyond their enhanced reduction capabilities, these electrons exhibit expanded electronic wave functions, greater mobility, and the potential for extended transport ranges when stabilized in excited states. However, their practical utilization is limited by significant energy losses due to phonon emission, resulting in rapid carrier cooling.

Recent studies have demonstrated that hot electrons can be effectively harnessed through ultrafast SE interactions in manganese (Mn)-doped QDs.^711–713^ These interactions enable exceptionally rapid energy transfer (>10 eV ps^–1^),^711^ potentially allowing for the capture, stabilization, and utilization of hot electrons before they dissipate energy via phonon emission. This strategy presents a promising avenue for advancing photoconversion and photochemical applications.

In ref 712, the SE energy transfer rate was directly quantified through comparative studies of Auger recombination in the Mn-doped CdSe QDs and reference undoped samples (both samples contained a thin protective CdS shell).^712^ In the undoped sample, biexciton (XX) Auger decay proceeds via the recombination of an electron–hole pair, with the released energy transferred to a third carrier (electron or hole). As shown in Figure 23A (left), the biexciton Auger recombination is characterized by a time constant of τ_XX_ = 30 ps for a CdSe core radius of ∼2 nm (Figure 23B, black), consistent with the universal “volume scaling” of Auger lifetimes.^699^ In this process, the acceptor carrier gains energy equivalent to the QD bandgap (*E*_g_), allowing the energy-transfer (or energy-gain) rate to be estimated as *r*_A,gain_ = *E*_g_/τ_XX_, yielding approximately 0.06 eV ps^–1^.

**Figure 23:**
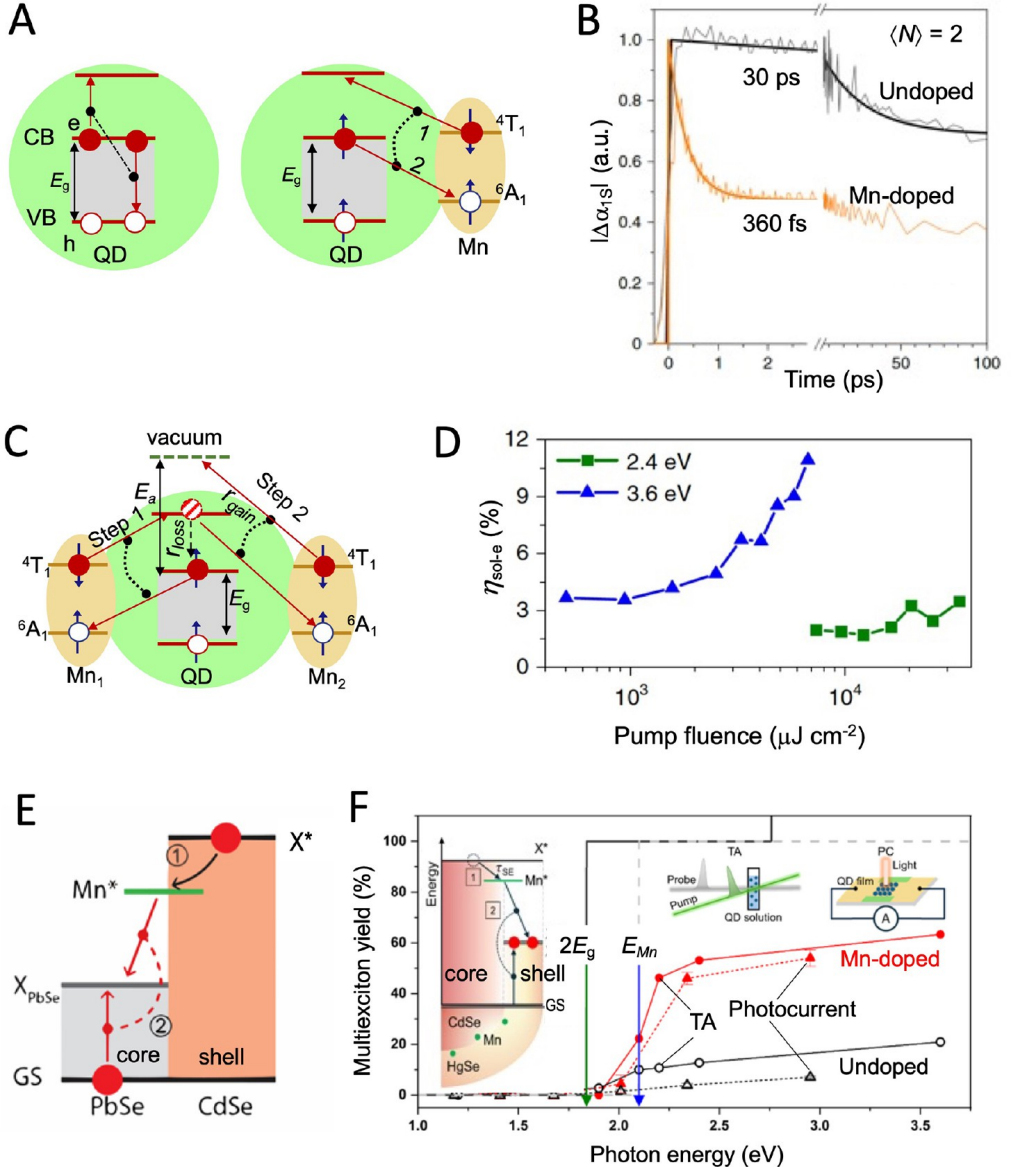
(A) Ordinary Auger recombination of a QD biexciton in an undoped QD (left) compared to SE-Auger recombination in a Mn-doped QD (right), involving a hybrid biexciton composed of the QD exciton and an excited Mn ion (Mn*) in its ^4^T_1_ state. The latter process occurs through two correlated SE steps: (1) spin-down transfer from Mn* to the excited electron state in the QD, followed by (2) spin-up transfer from the QD conduction band (conduction band = CB) to the Mn ion, restoring its ground-state (ground-state = GS) ^6^A_1_ configuration. VB = valence band. (B) Transient absorption (TA) measurements of biexciton decay in undoped (black trace) and Mn-doped (orange trace) CdSe QDs with a thin CdS shell (CdSe core radius ∼ 2 nm) reveal time constants of 30 ps and 360 fs, respectively (Δ*α*_1S_ represents the pump-induced change in the absorption coefficient at the band-edge 1S absorption peak). The pronounced acceleration of biexciton dynamics in the Mn-doped sample indicates that the rate of SE-type Auger interactions is significantly higher than that of ordinary Auger interactions. (C) QD ionization leading to electron emission occurs through a two-step SE-Auger re-excitation process, driven by successive energy transfers from two excited Mn ions. The high efficiency of this effect arises from the fact that the energy gain rate (*r*_gain_) from SE-Auger energy transfer exceeds the energy-loss rate (*r*_loss_) due to photon emission. (D) Internal quantum efficiency of solvated electron production (η_sol‑e_) using Mn-doped CdSe/CdS QDs excited by 190 fs pulses at 2.4 eV (green squares) and 3.6 eV (blue triangles). Panels (B) and (D) adapted with permission from ref 712. Copyright © 2022 Livache et al. under exclusive license to Springer Nature Limited. (E) Schematic representation of SE-CM in PbSe/CdSe core/shell QDs. This process occurs through two successive SE steps: (1) rapid SE-assisted capture of a hot exciton (X*) by the Mn ion, followed by its energy- and spin-conserving relaxation, resulting in the generation of two excitons (one dark and one bright) in the PbSe core. (F) Multiexciton yield as a function of photon energy for SE-CM in Mn-doped inverted CdSe/HgSe core/shell QDs, measured using TA spectroscopy (red circles) and a photocurrent technique (red triangles). Black symbols represent undoped QDs, which exhibit significantly weaker CM. The solid black line represents the multiexciton yield for ideal CM, where the quantum efficiency of photon-to-exciton conversion increases by 100% for each increment of photon energy by *E*_g_ above the CM threshold of 2*E*_g_. The dashed gray line represents the ideal SE-CM scenario, where the threshold is defined by *E*_Mn_. Adapted with permission from ref 714. Copyright © 2025 Noh et al.

The Mn-doped sample also exhibited a fast signal at comparable excitation levels; however, its time constant was almost two-orders of magnitude shorter (τ_XX,Mn_ = 360 fs; Figure 23B, orange). This striking reduction suggests a dramatic enhancement in the rate of Auger interactions, attributed to the involvement of a magnetic impurity. In this case, Auger recombination involved a hybrid biexciton, consisting of the intrinsic QD exciton and an excited Mn ion in its spin-3/2, ^4^T_1_ configuration. This process proceeds through two corelated SE steps (Figure 23A, right): (1) spin-down electron transfer from the 3d Mn shell to the excited (hot) electron state in the QD, accompanied by (2) spin-up electron transfer from the QD conduction band to the Mn ion. As a result, a hot exciton is generated in the QD, while the Mn ion relaxes to its ground-state spin-5/2, ^6^A_1_ configuration.

Based on these measurements, the SE-assisted energy transfer rate can be estimated as *r*_SE‑A,gain_ = *E*_Mn_/τ_XX,Mn_, where *E*_Mn_ = 2.1 eV represents the energy of the excited Mn ion. This yields a transfer rate of 8.4 eV ps^–1^. Not only is this value approximately 100 times higher than that of the conventional Auger process, but it also surpasses the energy-loss rate due to photon emission (*r*_loss_, generally less than ∼ 1 eV ps^–1^)^715^ by at least an order of magnitude.

These results provide direct experimental evidence that SE interactions enable efficient manipulation of hot carriers before they lose their kinetic energy to phonons.

The above assessment was recently confirmed by the demonstration of highly efficient SE-assisted photoemission driven by visible light pulses. This process was realized through a two-step SE-Auger re-excitation mechanism, in which a band-edge electron was excited to the vacuum state outside the QD via successive energy transfers from two excited Mn ions (Figure 23C).^712^ In undoped QDs, the second step of Auger re-excitation would typically be hindered by hot-electron relaxation through phonon emission. However, in Mn-doped structures, the exceptionally high SE energy transfer rate allows the hot electron to be efficiently excited to the vacuum state before undergoing phonon-assisted intraband cooling.

Yet another effect enabled by SE-Auger interactions is the high-yield production of solvated electrons.^712^ This process was realized with both UV and visible photons, with photon energies *E*_phot_ = 2.4 eV and 3.6 eV, respectively. In the UV-excitation case, generation of solvated electrons occurred via single-step Auger ionization, where a hot electron was ejected from the QD directly into the surrounding medium (water). In the visible-excitation case, the process proceeded through two-step SE-Auger ionization (Figure 23C). The maximum internal quantum efficiency (η_sol‑e_) reached 11% and 3.5% for *E*_phot_ = 3.6 eV and 2.4 eV, respectively (Figure 23D). These values compare favorably with other electron emitters, despite being achieved with significantly lower photon energies. For instance, studies of hydrogen-terminated diamond surfaces reported a η_sol‑e_ of ∼0.6% for deep-UV photons with *E*_phot_ = 5.86 eV.^716^

Ultrafast SE interactions can also facilitate the conversion of hot-carrier kinetic energy into additional electron–hole pairs, enabling high-efficiency carrier multiplication (CM). To achieve SE-driven CM (SE-CM), Jin et al.^713^ designed Mn-doped PbSe/CdSe core/shell QDs with a bandgap smaller than half of *E*_Mn_. In these structures, CM proceeded through two SE-mediated steps (Figure 23E): (1) SE energy transfer from a hot exciton delocalized throughout the QD to a Mn ion at the core/shell interface, followed by (2) energy- and spin-conserving relaxation of the excited Mn ion, generating two excitons (one bright and one dark) in the PbSe core. Due to the extremely short SE time scales, both SE steps occurred without significant interference from phonon emission, resulting in a high SE-CM efficiency. Notably, at *E*_phot_ = 2.4 eV—just 0.3 eV above the nominal SE-CM threshold defined by *E*_Mn_ = 2.1 eV—the multiexciton yield (the probability of generating a biexciton or higher-order multiexciton per absorbed photon) reached ∼50%, marking a more than 2-fold enhancement compared to undoped PbSe/CdSe QD reference samples.

In a more recent advancement, SE-CM was employed to enhance the photocurrent of a real-world photoconductive device.^714^ However, the Mn-doped PbSe/CdSe QDs studied in ref^713^ were not suitable for this demonstration, as the charge carriers generated through SE-CM remained confined within the PbSe core, effectively isolated from the external circuit by the wide-bandgap CdSe shell.

To address this limitation, Noh et al.^714^ developed Mn-doped inverted CdSe/HgSe QDs, where the lower-bandgap material (HgSe) was positioned in the shell region (Figure 23F, left inset). This design allowed both electrons and holes to be efficiently extracted into the external circuit. The QDs were characterized through TA spectroscopy in solution and photocurrent measurements in solid-state films (Figure 23F, top right insets). The results from both methods showed excellent agreement (Figure 23F, compare data shown by circles and triangles), clearly demonstrating the impact of SE-CM, as evidenced by a sharp increase in both the TA signal and the photocurrent just above the energy of the Mn spin-flip transition.

Although only recently discovered, ultrafast SE-type energy transfer has already demonstrated significant potential for advanced photoconversion applications. These include the generation of hot, free and solvated electrons by low-energy photons via SE-assisted photoemission, as well as enhanced carrier production through SE-CM. Such effects hold great promise for electro-optical devices and photochemistry. In the latter, the enhanced reductive power of hot or solvated electrons can be leveraged to drive challenging chemical reactions requiring a high reduction potential. Simultaneously, the pairwise generation of electron–hole pairs via SE-CM can improve the efficiency of multistep, multielectron/hole chemical reactions by alleviating bottlenecks associated with the waiting time between successive reduction/oxidation steps.

### Quantum Light Sources

An emerging part of nanoscience with NCs is quantum light generation. Conversely to classical sources, quantum light sources prepare states with defined correlations between photons, with single-photon states being the canonical example. Photonic quantum states exist as Eigen or superposition states within the vector space spanned by the photon’s spatiotemporal mode, frequency, and polarization. Entangled photons are then superposition states of a group of photons, where the state of each photon depends on the state of other photons, even if separated by large distances. The preparation of such quantum states is an outstanding problem, particularly when using device-compatible solid-state systems. Material systems are needed that allow the faithful, *i*.*e*., coherent transcription between electronic excitations and photons and with a defined number of quanta.^717^ Single photons that are indistinguishable in all degrees of freedom can be interfered with to build up higher-order quantum states and are, therefore, versatile building blocks and a desirable output for quantum light sources. Colloidal QDs have long been proposed for the emission of single photons owing to their size-confinement restricting the number of electronic excitations,^718^ effectively acting as a two-level system (TLS). QDs have indeed served as model systems in studies of single-emitter photophysics even before quantum light sources moved into the spotlight of engineering efforts. Key milestones included the demonstration of single-photon generation,^719^ Purcell enhancement of the emission,^720^ and studies of discrete spectral jitter,^721^ and decoherence.^722^ Years of continuous improvements in materials and measurement techniques, largely motivated by fundamental science questions, have recently led to renewed interest in QDs as single-photon emitters (SPEs), partially spurred by accelerating interest in practical quantum technologies. Several review articles have specifically addressed the application of QDs in quantum science more broadly, including applications in coupled excitonic arrays.^723,724^ Here, we discuss the most recent advances made in QDs as emitters of single photons in the context of specific performance metrics of future single-photon sources.

#### Single-Photon Purity

The single-photon purity of a quantum emitter describes the ratio between one-photon to multiphoton emission events, typically measured under pulsed laser excitation with a second-order intensity correlation measurement *g* ^(2)^(τ = 0). An ideal SPE does not permit simultaneous emission of multiple photons. As QDs can host multiple excitations, single-photon emission requires targeted optimization, either via spectral rejection of multiexcitons or increasing their nonradiative Auger rate. The former strategy relies on relatively large biexciton binding energies and high spectral stability with narrow emission lines, but is in principle straightforward to implement in analogy to epitaxially grown quantum wells.^725^ The latter strategy benefits from long radiative lifetimes of the emitter, which is detrimental for many applications. The Auger rate in II–VI QDs is increased with quantum confinement, which typically degrades the stability of the QD and induces spectral jitter at low temperatures.

Recent work on InP and lead-halide perovskite QDs has helped overcome these limitations. InP/ZnSe QDs have shown improved single-photon purity *g* ^(2)^(τ = 0) = 0.03 (average 0.19) compared to CdSe/CdS QDs with simultaneously improved spectral stability and reduced blinking, even at high excitation fluence close to saturation (Figure 24C).^254^ This remarkable behavior of near absence of blinking in the presence of fast (70 ps) Auger recombination—causing charge-induced intermittency in II–VI QDs—justifies further investigation. For applications not requiring a high degree of optical coherence at low temperatures, InP-based materials might provide the needed stability.

**Figure 24:**
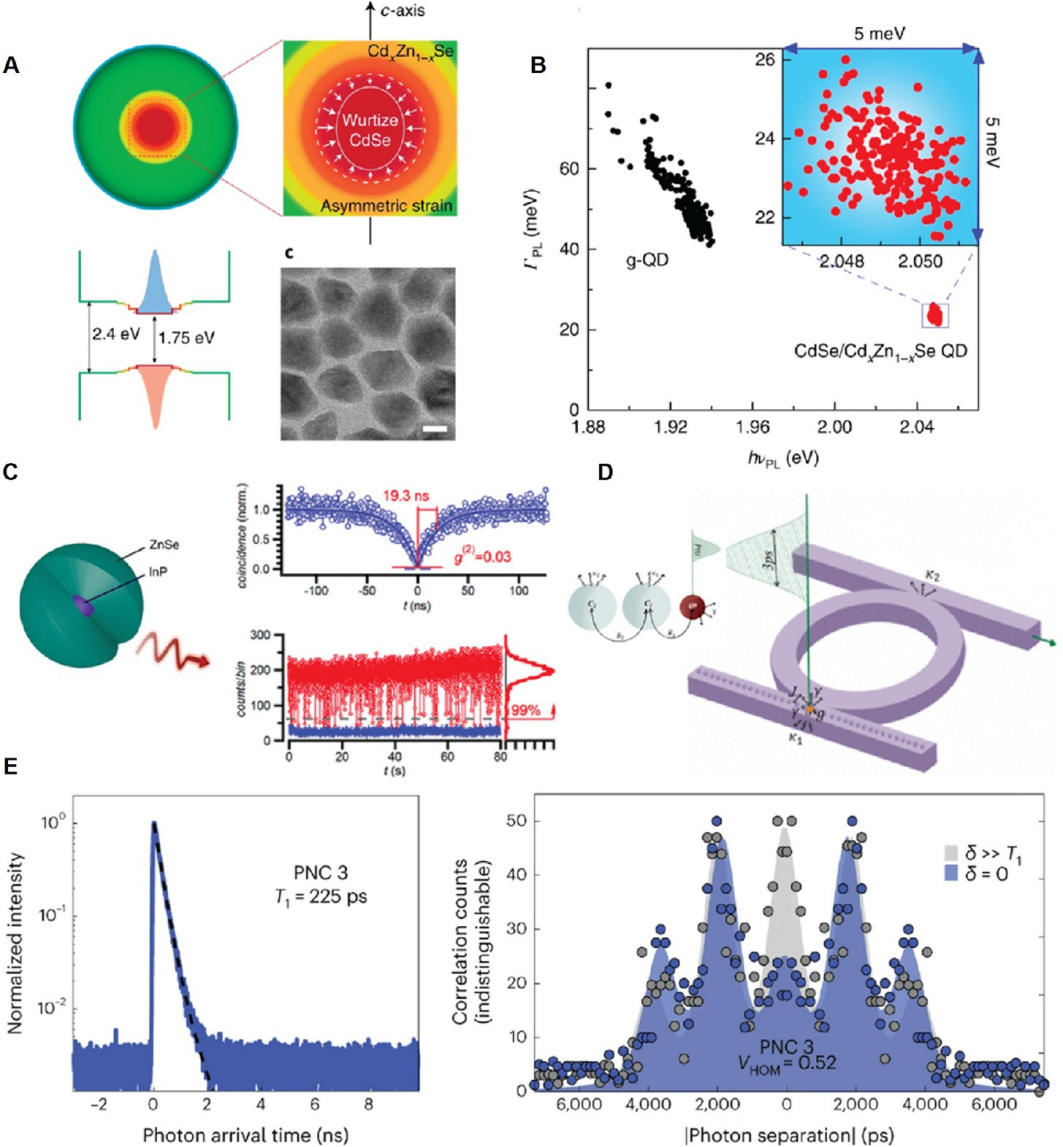
(A) Strain engineering of Wurtzite Cd_*x*_Zn_1–*x*_Se. (B) The highly strained thick-shell particles exhibit substantially narrower single-dot line width compared to state-of-the-art CdSe. Panels (A) and (B) reproduced with permission from ref 730. Copyright © 2019 Park et al. under exclusive license to Springer Nature Limited. (C) Antibunching signature and emission intensity trace of a single InP/ZnSe QDs. The emission is remarkably stable even in near-saturation conditions. Reproduced from ref 254. Copyright © 2017 American Chemical Society. (D) Proposed dual-cavity architecture for the coupling of colloidal QDs to SiN waveguides. The rational cavity design may yield on-chip single-photon sources with engineered performance. Reproduced from ref 748. Copyright © 2019 American Chemical Society. (E) The time-resolved PL of a single CsPbBr_3_ QD at 4K reveals purely radiative lifetimes of around 200 ps. Commensurate optical coherence times allow the amplitude interference of two indistinguishable single photons emitted from the same QD, manifested as a dip in the coincidence center peak recorded after a beam splitter (Hong-Ou-Mandel dip). Reproduced with permission from ref 736. Copyright © 2023 Kaplan et al. under exclusive license to Springer Nature Limited.

With the advent of perovskite QDs, several studies specifically addressed the single-photon purity. Initial experiments on CsPbX_3_ X = Br, I, showed room-temperature purities of ∼6%, but also revealed problems of particle instabilities and blinking.^331^ Quantum confinement has improved the best values to around 2%^331^ with a distinct increase in purity with decreasing size. Complementary work has further shown that single particles in solution can have average single-photon purities of 2% in the strong confinement regime, even though the stability of these particles did not permit extended single-particle spectroscopy.^726^ Single-photon emission is further retained for different stoichiometries of A-site cations from FA to cesium in APbBr_3_ QDs^727^ and with changing halide composition,^726^ furthering the prospect of color-multiplexed single-photon emitting devices using such QDs. Remarkably high count rates up to 9 × 10^6^ with purities around 2% at room temperature have enabled quantum random number generation conforming to National Institute of Standards and Technology standards.^728^ The critical challenge in increasing the single-photon purity of these QDs at room temperature via confinement will be to increase the long-term stability.

#### Optical Coherence, Coherent Fraction, and Spectral Stability

An ideal SPE behaves akin to a quasi TLS with a single Lorentzian line, the width of which is only determined by the spontaneous emission lifetimes without further decoherence. Solid-state SPEs are limited in the fraction of such coherently emitted photons by phonon side-bands. The coherence time is further reduced owing to spin- and phonon-bath interactions, and charge- and dielectric fluctuations further induce spectral jitter *i*.*e*., spectral diffusion. To render colloidal QDs suitable as coherent SPEs, the processes need to be minimized, which has also been summarized in terms of design rules for minimizing the optical emission line width.^729^

Decades of work in II–VI QDs have provided the microscopic origin to these physics. We have reached a good understanding of how lattice strain and spin–orbit terms induce fine-structure splitting, how resonant phonon-mediated exchange between fine-structure states leads to pure dephasing of the optical transition, and how exciton–phonon coupling defines the broad room-temperature line shapes and causes phonon side-bands at low temperatures.

Synthetic strategies to improve the room temperature color-pure emitters in LEDs can similarly be translated into color-multiplexed single-photon sources. CdSe/ZnSe QDs have reached a remarkably narrow line width of around 20 meV, achieved via strain engineering (Figure 24A,B).^730,731^ For perovskite QDs, low energy surface phonon modes have been removed from the spectral density to achieve down to 35 meV line width.^329^ The line width, or inversely the coherence time, is more critical at low temperatures, where the generation of indistinguishable single-photons becomes feasible. For II–VI QDs, the degree of optical coherence *T*_2_/2*T*_1_ is insufficient regardless of the applied technique, and pronounced charging-induced blinking and spectral diffusion of II–VI QDs are at odds with the requirements of indistinguishable single-photon generation. InP/ZnSe/ZnS QDs have been demonstrated as remarkable alternatives with 250 ps coherence times at low temperatures, substantially reduced spectral jitter, and high photostability.^732^ In this study, the observed single-photon purity was 0.07, which may be improved with spectral filtering in practical applications. As an impediment to applications in quantum optics, the lowest-lying exciton fine-structure state in InP is nominally dark, leading to delayed emission with lifetimes on the order of microseconds, which can reduce the single-photon purity under high repetition rate excitation. Even the bright-state lifetime of 15 ns is still orders of magnitude longer than the coherence time, which will require substantial radiative enhancement with photonic structures in future sources of indistinguishable single photons. Nevertheless, the high intensity and spectral stability of InP QDs render them a promising platform for further optimization.

The challenge of incoherent emission of QDs is dismissed in perovskite QDs, which show a remarkable combination of unusually fast (200 ps) radiative lifetimes,^332,333^ long 80–200 ps optical coherence times, and minimal phonon sidebands.^733–735^ Reduced spectral jitter, likely owing to the relative absence of surface trap states, makes spectral filtering of multiexciton emission straightforward to achieve high single-photon purity *g* ^(2)^(τ = 0) < 0.05. This remarkable combination of properties led to the recent observation of Hong-Ou-Mandel interference in perovskite QDs,^736^ a hallmark demonstration of quantum optical phenomena in SPEs previously reserved to epitaxial QDs and defects in diamond. Optical coherences of *T*_2_/2*T*_1_ of up to 0.55, absent any cavity integration (Figure 24E). The further optimization of perovskite QDs as quantum emitters will rely on the in-depth understanding of the relationships between the morphology of nanosized perovskites and the electronic structure and optical response. Control over the fine-structure splitting may reduce optical dephasing and allow the generation of entangled photon pairs via the biexciton–exciton cascade, but is complicated by recent findings pointing to an avoid crossing of fine-structure splitting intrinsic to the polaronic lattice distortion, thus complicating the control of the splitting with synthetic targeting of the shape and lattice anisotropy.^737^

Harnessing charged perovskite QDs with faster lifetimes may be a promising approach to increasing the optical coherence, although initial work showed that the trion is more strongly coupled to optical lattice modes, which may reduce the optical coherence.^738^ Perovskite NPLs might have higher optical coherence owing to their faster radiative decay times.^739^ However, very few studies have addressed the properties of individual platelets, and the fine-structure splitting is generally found to be larger than in cubes.^740^ The criticality of surface phonons has also been confirmed at low temperatures, where rational ligand design improved the coherence time and stability. Remarkably, the same work identified inhomogeneous broadening at low temperatures that approaches typical levels of epitaxial QDs.^741^ The application of electric fields is a particularly promising direction for tuning the electronic structure and spectral stability in perovskite QDs. As such, static electric fields have been shown to remove the fine-structure splitting in CsPbI_3_ QDs and mitigate spectral diffusion.^742,743^ The relative simplicity of static E-field imposition compared to magnetic fields paired with the ever-improving synthetic procedures foretell a bright future for perovskite QDs as a source of indistinguishable single-photons, especially as efforts for cavity-and device integration are just beginning.

#### Device Integration

Besides the materials science aspects of the material generating single-photons, the integration of QDs into devices is equally important for applications.^744,745^ Two broad challenges are to be addressed: the coupling to nanophotonic architectures and the electrical addressability. The former serves to increase the emission rate to achieve higher bit-rates in single-photon devices^746,747^ and the indistinguishability^748^ of single photons (Figure 24D). This integration is discussed in the context of ‘hybrid-integration’ of disparately grown emitters with on-chip photonic circuits.^749^ Several avenues have addressed the main challenge of deterministic placement of QDs in on-chip devices, including template-assisted self-assembly,^750^ contact printing,^751^ and DNA-origami strategies.^752^

The electrical addressability of single QDs will benefit from the knowledge base developed for classical QD LEDs, but it is not without challenges. Nonbalanced charge injection can cause instabilities in the emission.^753^ On the other hand, the single-photon purity of electrically excited single QDs is consistently higher than under optical excitation, owing to a reduction in the spurious background emission under optical excitation. On the other hand, the sequential carrier injection can slow the formation of multiple excited states, in principle improving the single-photon purity, as long as simultaneous device instabilities can be avoided.^754^

#### Collective QD Multiphoton Emission

Artificial atoms, like colloidal or epitaxially grown QDs, are extensively explored for the generation of multiphoton emission. Epitaxially grown QDs are indeed capable of generating entangled photon pairs via the biexciton (XX) to exciton (X) radiative cascade process.^755,756^ Alternatively, by properly adjusting the X and XX energy levels, twin photons could be generated despite the very low yield of particles satisfying the rigid constraint of having degenerate X and XX excitonic states.^757^ Colloidal QDs have been notoriously affected by the fast nonradiative Auger process which renders multiexciton states poorly emissive. Therefore, the schemes explored by employing epitaxially grown QDs remained elusive for the generation of more complex quantum light fields.

The advent of perovskite QDs operating in the weak confinement regime, with strongly reduced Auger recombination rates,^332^ could bring colloidal QDs on par with what has been so far achieved employing epitaxially grown QDs. In fact, very bright emission from XX exciton states has been reported by several groups,^758–760^ but the current limited control over the fine structure sublevel exciton states,^334,742,761^ renders the generation of entangled photon pairs not feasible. Alternative strategies in addition to electrical and strain engineering could be devised to reach this goal.

Beyond single QDs, coupled QD systems are an elegant and alternative avenue for the generation of multipartite, N-photons states. Very recently, a solid-state source of photon triplets has been demonstrated employing an epitaxial QD molecule.^762^ The main challenge with epitaxially grown QDs is the limitation in scaling up the number of coherently coupled QDs, which remains limited to only a few emitters.^763^

As outlined above, perovskite QDs are excellent sources of coherent single photons,^733,764^ with record fast radiative lifetime.^332,334^ In addition, such emitters can be assembled in very well-defined 3D SLs,^339^ by a self-drying mediated process. Contrary to the spontaneous emission of photons, coupling among several excited QDs, mediated by the common vacuum modes, could become strong enough to enable the formation of a giant dipole and the radiation of a burst of photons.

In 1954, Dicke predicted that an ensemble of N identical TLS, here exemplified by photoexcited excitons in QD SLs, confined in a volume smaller than about λ^3^ (where λ is the corresponding emission wavelength of the TLS) can exhibit coherent and cooperative emission. If the excited TLS are initially fully uncorrelated, the coherence can be established through spontaneously triggered correlations due to quantum fluctuations (Figure 25A). When this occurs, a so-called SF pulse is emitted (Figure 25B). Coherent SF bursts of photons are characterized by an accelerated radiative decay time τ_SF_ ∝ τ_SE_/*N*, where the exponential decay time τ_SE_ of spontaneous emission from the uncoupled TLS is shortened by the number of coupled emitters *N*. In addition, SF exhibits the following fundamental signatures, the magnitudes of which are also dependent on the excitation density (or number of coupled QDs): (i) a delay or build-up time τ_build‑up_ ∝ log­(*N*)/*N* during which the emitters couple and phase-synchronize to each other, and which corresponds to the time delay between the excitation and onset of the cooperative emission (Figure 25A); and (ii) coherent Rabi-type oscillations in the time domain due to the strong light–matter interaction, known as Burnham–Chiao ringing (Figure 25B).

**Figure 25:**
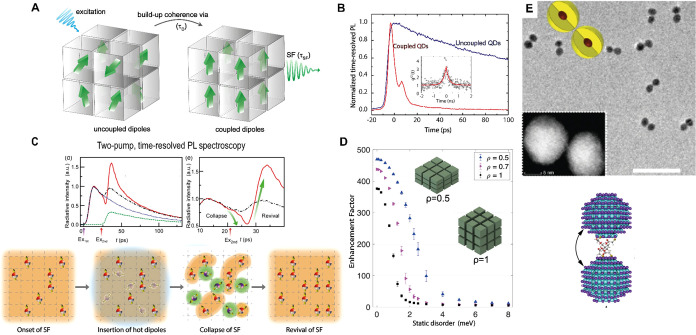
Coupled QD emission. (A) Schematic of the build-up process of SF: an initially uncorrelated ensemble of TLS (randomly oriented green arrows) is excited by a light pulse (blue arrow, top left). After time τ_D_, their phases are synchronized (aligned green arrows) such that they cooperatively emit a SF light pulse (red arrow at right) with a characteristic decay time τ_SF_. Reproduced with permission from ref 511. Copyright © 2018 Springer Nature Limited. (B) Time-resolved decay traces for the two emitting bands, showing a strongly accelerated decay for the SF band. The presence of oscillations in the time domain are a very peculiar feature of superfluorescent emission of multiphotons burst. Inset: an example of superbunching with *g* ^(2)^(0) > 2 from a single SL. Reproduced with permission from ref 773. Copyright © 2020 The Materials Research Society. (C) Echo-like SF behavior under a controllable disturbance, highlighting the collapse and the revival of the collective state after hot dipoles injection. Reproduced with permission under CC BY 4.0 from ref 767. (Copyright © 2023 Wang et al. Open access). (D) Comparison of the robustness of the superradiant enhancement factor to static disorder in SLs of different NC aspect ratios. Reproduced with permission under CC BY 4.0 from ref 770. (Copyright ©2023 Ghonge et al. Open access). (E) Scheme for fabrication of coupled CdSe/CdS colloidal QD molecule and a exemplary TEM image of a QD dimer. Reproduced with permission under CC BY 4.0 from ref 771. (Copyright ©2019 Cui et al.open access).

Time-resolved PL decay measurements reported in Figure 25B revealed an accelerated PL decay of the SF emission peak compared to the PL decay of uncoupled QDs, as predicted by Dicke for superradiant emission. Considering the change in radiative rates, it was possible to estimate that more than 20 QDs contribute collectively to the emission process.^511^ This is an order of magnitude more than what has been achieved with any other solid-state quantum emitters.^763^ Interestingly, the coherent coupling also affects the second-order coherence of the emitted light, as evinced by the photon statistics of the arrival time on a detector. It is well known that coherent light has a random distribution (Poissonian) of the photon arrival times while a TLS shows photon antibunching (sub-Poissonian distribution). In contrast, the cooperative emission from the coupled QDs leads to coherent multiphoton emission bursts which manifest itself in a bunching peak in the second-order correlation function (Figure 25B).

These peculiar features of SF were first observed in perovskite monocomponent QD SLs,^511^ attesting the capabilities of time-correlated multiphotons via the SF process. Subsequent studies have then explored the rich physics behind this rather unconventional process. By exploring binary QD SLs,^515^ it has been observed that the onset of SF is crucially dependent on the volume fraction of the excited QDs. ABO_3_-like SLs exhibit the lowest threshold while NaCl-like SLs were unable to sustain collective emission despite very good structural homogeneity. In fact, the remaining energetic disorder among the QDs and the finite dephasing time, require a sizable number of coupled QDs, and consequently a strong coupling strength, for SF to occur. This explains why SLs with low NC fraction were unable to sustain collective emission.

Similarly, strong exciton–phonon interactions, inducing a net acceleration of the exciton dephasing time, render the collective coupling less efficient. In fact, a much larger τ_build‑up_ and a higher threshold are found at higher temperatures, with typical SF features persisting up to 105 K,^515^ but eventually vanishing entirely at even higher temperatures. Employing bulk-like perovskite compounds, the SF regime has been pushed to room temperature,^765,766^ despite a clear consensus on the underlying photophysics (*e*.*g*. whatever or not the polaron formation is responsible for an elongated coherence) has not emerged yet.

The delicate coherent coupling responsible for collective emission, has been very recently further exploited by a two-pump, time-resolved PL spectroscopy.^767^ The collective response of macroscopic quantum states under perturbation is widely used to study quantum correlations and cooperative properties, such as defect-induced quantum vortices in Bose–Einstein condensates and the nondestructive scattering of impurities in superfluids. Similarly, the SF effect, enabled by dipole–dipole coupling through virtual photon exchange, leads to the macroscopic, giant dipole moment which can be perturbed by the injection of uncorrelated dipoles. As shown in Figure 25C, echo-like behavior is observed in a cooperative exciton ensemble under a controllable perturbation, corresponding to an initial collapse followed by a revival of the SF collective emission. Such a dynamic response could refer to a phase transition between the macroscopic coherence regime and the incoherent classical state on a time scale of 10 ps. The echo-like behavior is absent above 100 K due to the instability of the photogenerated giant dipole, as a result of phonon-induced exciton dephasing. Experimentally, the SF response to perturbations is shown to be controlled by the amplitude and injection time of the perturbations. Such a phase transition, and the occurrence of collective emission has been recently postulated to serve as a platform to simulate and investigate the physics of correlated quantum materials.^768^ The ultrafast optical injection of quantum confined excitons plays the role of doping in real materials, and, at large photodoping level, the exciton gas undergoes an excitonic Mott transition, which fully realizes the magnetic-field-driven insulator-to-metal transition described by the Hubbard model. At lower photodoping, the long-range interactions drive the formation of a collective superradiant state, in which the phases of the excitons generated in each single perovskite QDs are coherently locked.

While synthetic efforts resolved in a vast library of SLs,^516,517^ currently more than 20 different crystals have been realized including lamellar 1D linear chains of perovskite QDs, theoretical modeling with progressively higher complexity has elucidated the occurrence of SF emission and critically highlighted the role of thermal dephasing and energetic disorder.^769^

Recently, a comprehensive theoretical work^770^ has modeled the onset of collective QD response in SLs of different dimensionalities (1D, 2D, and 3D) with variable QD aspect ratios (Figure 25D). They predicted as much as a 15-fold enhancement in robustness against realistic values of energetic disorder in 3D SLs composed of cuboid-shaped, as opposed to cube-shaped NCs. Superradiance from small (*N* ≲ 10^3^) 2D SLs is up to ten times more robust to static disorder and up to twice as robust to thermal decoherence than 3D SLs with the same *N*. As the number of *N* increases, a crossover in the robustness of superradiance occurs from 2D to 3D SLs. For large *N* (>10^3^), the robustness in 3D SLs increases with *N*, showing cooperative robustness to disorder. Despite this theoretical effort still considers a low number of coherently excited QDs (single exciton regime), it helps rationalizing the critical role played by several material parameters (mainly exciton coherence time and energetic disorder in the ensemble) and could guide the design of SLs which sustain SF and coherent multiphoton emission even at room temperature.

So far, collective coupling occurs in SLs which are significantly larger than the wavelength of the emitted photons, and the number of coherently coupled QDs is not well defined but self-limited by the optical properties of constituent build block, *e*.*g*. through their mutual energetic disorder and characteristic dephasing time. To control the number of emitted photons, a possible solution could be to control, via bottom-up assembly strategies, the number of coupled QDs. Recently, a QD molecule consisting of CdSe/CdS core/shell QDs has been realized with a very high yield of dimers formation (Figure 25E).^771^ Coherent coupling and wave function hybridization were manifested by a redshift of the band gap, in agreement with quantum mechanical simulations. Similar to atomic systems which could form molecules and polymer chains once brought into vicinity, the close proximity of two (or more) QDs lead to the formation of hybrid states with electron wave function spreading over the entire QD molecule.

Interestingly, QD molecules were found to reversibly switch between two emission colors, characteristic of the individual QD building block, without intensity loss.^772^ Because the electronic wave functions of both QDs are delocalized over the entire QD molecule, an applied electric field can shuffle electrons between the two QDs thus switching the emission color. Appealingly, the concept is highly engineerable: the specific emission centers of the QD molecule can be easily adjusted by the size, composition and possibly even the number and shape of the constituent QDs. This could realize electrical multiplexing color switches at the single photon level. The technology seems to be scalable and a higher number of QD assemblies could be engineered toward more complex quantum light sources. The grand challenge is to now couple two QDs with very similar, likely degenerate exciton states, thus favoring the formation of SF and coherent multiphoton emission in a control manner.

### Quantum Information Science

Colloidal semiconductor NCs present an excellent platform to explore the fundamental physics and chemistry of optical materials for quantum information science and to realize solid-state technologies for applications in quantum computation, sensing, simulation, and communication.^723^ Colloidal semiconductor NCs are prized for their optical properties as excitons are spatially confined within, and for particles smaller than or comparable in size to the excitonic Bohr radius, delocalized over the NC volume.^444,774–778^ Semiconductor NCs can also host quantum point defects, localized optically active impurity or vacancy centers and their complexes, known as color centers or phosphors, with charge or spin degrees of freedom.^779–782^ These excitonic, charge, or spin-states can be isolated within individual NCs creating the requisite TLSs for quantum bits, known as qubits. These qubits can be optically initialized to set the quantum state, manipulated through interactions to control the quantum dynamics to process information, and read out optically or electrically to measure the output.

The required characteristics of the NCs are different for various applications. For example, excitons with short lifetimes, and even when transform limited, with similar, but short optical coherence times are suitable as bright sources of indistinguishable single photons needed for use in quantum photonic circuits.^733^ Spin qubits, with longer lifetimes, and thus coherence times, are required for quantum storage and manipulation.^783–785^ Regardless of the application, colloidal NCs present a number of advantages, in comparison to bulk materials, in studying photon and spin qubits and integrating these qubits for use,^723^ as dispersions in fluids for sensing or as single NCs or NC arrays on surfaces and in optical cavities for computation and communication.

The negatively charged, nitrogen-vacancy (NV-) center (Figure 26A, inset) in the ultrawide bandgap semiconductor diamond is a well-studied quantum point defect and a prototypical spin qubit.^786,787^ Diamond has a low nuclear spin bath and low spin–orbit coupling, limiting interaction with phonons and magnetic and electronic noise. The NV-center introduces dipole-allowed, molecular-like s- and p-states, deep within the diamond bandgap (Figure 26B), creating spin-triplet and spin-singlet manifolds that are coupled by intersystem crossing (dashed arrows). Preferential intersystem crossing allows nonresonant optical excitation (green arrow) to preferentially populate the *m*_s_ = 0 sublevel, *initializing* the spin state. The spin-state dynamics can be *manipulated* by external magnetic and electric fields, strain, temperature, and pressure. Differences in the spin-state energy and lifetime create contrast in the visible fluorescence (green arrows), providing a scheme for *readout*.

**Figure 26:**
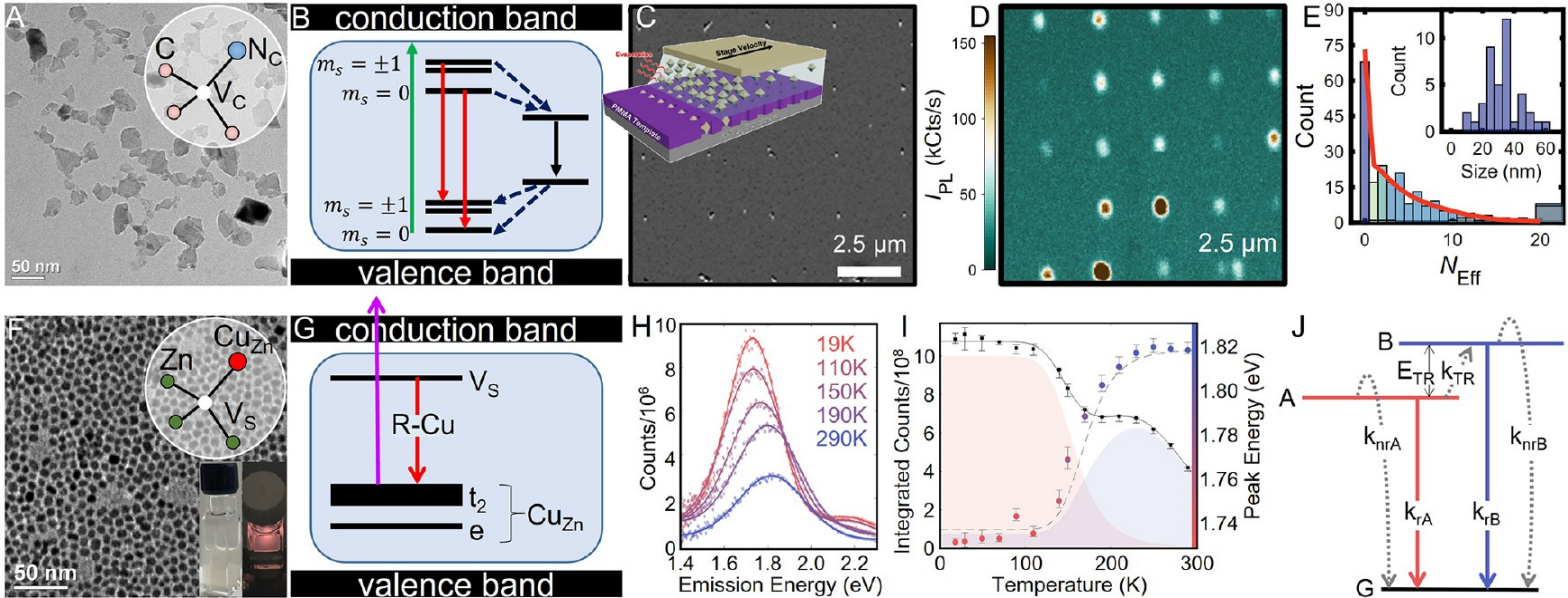
(A) TEM image and (B) energy level diagram of low-fluorescence, colloidal, milled nanodiamonds doped with NV-centers. Inset (A): schematic of the NV-center in diamond.^796^ (C) Schematic and AFM image of the template-assisted self-assembly of colloidal nanodiamonds. (D) PL map of assembled fluorescent nanodiamonds. (E) Statistical characterization of the number of NV-centers (*N*_eff_) in individual nanodiamonds characterized from autocorrelation measurements, where *N*_eff_ = 0 (purple), 0 < *N*_eff_ < 1.5 (yellow-green), 1.5 < *N*_eff_ < 2.5 (green), and *N*_eff_ > 2.5 (blue). (Inset) AFM height distribution for the nonfluorescent nanodiamonds. (F) TEM image and (G) schematic energy diagram of colloidal, wet-chemically synthesized ZnS:Cu NCs. Inset (F): schematic of the Cu_Zn_-V_S_ center in ZnS. (H) Temperature-dependent PL spectra of ZnS:Cu NCs. (I) Integrated PL intensity (black symbols) and peak energy (colored symbols) as a function of temperature, extracted from Gaussian fits of data in (H). The solid black curve is a fit to the intensity data and the dashed colored curve is a sum of the emissions, weighted by their corresponding best-fit emission intensities, consistent with (J) an energy level diagram of two manifolds of states created by the defect, inside the ZnS bandgap, with coupled relaxation processes. Red and blue shaded regions in (I) represent the relative temperature-dependent intensities *I*_A_(*T*) and *I*_B_(*T*) from the best-fit model. Panels (A–E) reproduced from ref 796. Copyright © 2022 American Chemical Society. Panels (F–H) reproduced from ref 806. Copyright © 2023 American Chemical Society.

While the best NV-center spin coherence is seen in bulk diamond crystals, < 100 nm nanodiamond particles can be dispersed in solvents to form colloids, particularly interesting for *in vivo* and *in vitro* sensing.^782,787^ However, chemically pure, nanodiamonds with stable NV-centers are created by milling bulk diamond crystals, yielding particles varying in size, shape, surface chemistry, and the number of NV-centers, and thus with inhomogeneity in their optical and quantum optical properties (Figure 26A). Template-assisted self-assembly^788–791^ and optical trapping^792,793^ techniques have been used to deterministically and scalably position arrays of single and countable numbers of NCs on surfaces and in optical cavities.^794,795^ The assembly of NCs on surfaces, in addition to allowing the study of single NC photophysics, reduces the number of defects within the beam spot and therefore the purity requirements from <100 ppt in bulk crystals to <100 ppm in NCs.^723^ Figure 26C shows a schematic of the template-assisted self-assembly process, in which topographical trap sites created in resist templates allow capillary-driven assembly of commercial, ∼40 nm diameter nanodiamonds (Figure 26A).^796^ Atomic force microscope (AFM) measurements (Figure 26C), after the resist is removed, shows an example assembly of an array of single nanodiamonds, achieved with ∼80% yield. Spatially resolved PL (Figure 26D), autocorrelation, power-dependent intensity, and spin lifetime measurements of these samples allow statistical characterization of the nanodiamonds. For example, measurements of >200 commercial, low-fluorescence, milled nanodiamonds show that 31% of nanodiamonds have no NV-centers, 12% have single emitters, and the remainder have multiple NV-centers. The non-Poissonian distribution of emitters is hypothesized to arise from inhomogeneity in the nitrogen incorporation during the parent single crystal growth^797,798^ and the stochastic creation of NV-centers. Most studied milled nanodiamonds have surface carboxyl groups with patchy coverage, reported to reside at undercoordinated carbon sites between crystal facets.^799,800^ While sufficient to allow their aqueous dispersion, it is also difficult to functionalize nanodiamonds to both stabilize the particles in fluids and maintain their quantum optical properties, prompting the development of surface coatings, important for their use as sensors.^801^

While nanodiamonds are a relatively mature technology with desirable quantum optical properties, the discovery of new materials and spin qubits is needed for applications.^784,802^ In contrast to milled nanodiamonds, the wet-chemical synthesis of colloidal NCs allows the preparation of a wide range of semiconductor NC compositions (*e*.*g*., II–VI, III–V, IV–VI, and metal-halide perovskite NCs) with near-atomic precision in control over size and shape. The surface chemistry of these NCs is better understood and tailorable using known organic and inorganic ligand libraries.^415^ Advantageously, wet-chemical synthesis also allows the more rapid preparation of NCs with different defects, *e.g*., in comparison to implantation in bulk crystals, to facilitate quantum point defect discovery. With control over NC size and shape and the achievement of complex core–shell and Janus structures, NCs also allow wave function engineering to sculpt the spatial distribution of charge and spin states.^723^

Like diamond, ZnS has a dilute nuclear spin bath and low spin–orbit coupling. Impurity-doped ZnS is among the best-known phosphor materials and ZnS has recently been explored to host quantum point defects.^803,804^ Cu-doped ZnS (ZnS:Cu) emits in the visible red, green, and blue light from color centers known as R-Cu, G-Cu, and B-Cu.^781,805^ The R-Cu emission is reported to originate from a Cu_Zn_-V_S_ defect complex, which is a more dipole-allowed radiative transition (unlike ZnS:Mn that arises from intra-d-shell, dipole forbidden transitions) with the same symmetry as that of the NV-center in diamond (Figure 26A–F). Recently, red-emitting, colloidal ZnS:Cu NCs were reported (Figure 26F).^806^ The red emission is consistent with that seen in bulk materials arising from localized electronic transitions associated with the Cu_Zn_-V_S_ defect complex (Figure 26G). Temperature- and time-dependent optical spectroscopy (Figure 26H) has been used to map the peak energy, intensity, and lifetime of the red emission. A blue shift and a plateau in the intensity dependence (Figure 26I) of the red emission with increasing temperature is empirically modeled by the thermally activated carrier transfer between two manifolds of radiative states (Figure 26J). Room temperature quantum emission has now been observed from Cu_Zn_-V_S_ quantum point defects in single ZnS NCs.^807^ Understanding the luminescence characteristics of the defects is an important first step. Further studies, using electron spin resonance and optically detected magnetic resonance are important to understanding the spin-dependent optical properties of the defects, toward developing protocols for quantum point defect initialization, control, and readout. Studies of single quantum point defects in NCs are important to learning their location within the NC, concentration, and charge state, which can be manipulated by engineering the NC surface chemistry, exploiting their large surface-to-volume ratio to passivate surface states and remotely dope the NCs. The quick, controlled, and scalable synthesis of quantum point defects in colloidal NCs and the assembly of individual NCs on surface and in cavities make NCs excellent materials as light-matter interfaces to explore fundamentally and for applications in quantum technologies.^796^

### NCs for Catalysis

Colloidal NCs have been used as precursors of active and selective heterogeneous catalysts for over two decades now. There are multiple reasons that make colloidal NCs very relevant in advancing catalytic applications: (1) they can be used as model systems where several crucial parameters for catalytic activity (exposed facets, composition, metal–support interactions, *etc*.) can be tuned and utilized in advancing fundamental understanding of active sites and motifs under catalytically relevant conditions of temperature and pressure; (2) they can be engineered to display the largest fraction of active sites, when active site motifs are known, for a specific application or reaction; (3) they can be utilized to follow and observe processes of catalysts restructuring, activation and deactivation, given the uniform nature of surface structure and composition; (4) the NC-ligand interface can be engineered and utilized to display catalytic properties that differ from classic systems, such as supported catalyst. For the above reasons, research in colloidally prepared catalysts has been expanding in the recent years.^808,809^ Industrial uses of these catalysts have also been realized, although only for small-scale applications so far.^810^ Continued research in this field is expected to expand application areas and deepen fundamental knowledge, while also enhancing their impact in practical applications. A few case examples from recent years illustrate the potential and promise of this approach.

The use of colloidal NCs as precursors for supported catalysts has been one of the first applications of these controlled materials in the field.^811,812^ In analogy to single-crystal surfaces, NCs with well-defined sizes and facets allow to control the type and number of active sites exposed to the reaction (unless restructuring occurs, see below).^813,814^ The fraction of sites can then be studied as a function of catalytic performance to evaluate whether certain sites are majorly responsible for the reactivity patterns.^815,816^ The development of theoretical models, especially using advanced computational tools, to explain the reactivity patterns is equally useful. As an example of the latter, composition- and size-controlled alloyed Pd/Pt NC catalysts are useful to show the importance of step sites on the reactivity for propene combustion.^817^ As NCs vary in size between ∼2 and ∼10 nm, the fraction of terrace, edge and kink sites change accordingly, and comparing accurate turnover frequency values with the computationally modeled fraction of exposed sites allow proposing the hypothesis that pairs of atoms with specific coordination numbers (7–7 step sites, *i.e*., coordinated to 7 near neighbors) are the most active sites for propene combustion under reaction conditions (Figure 27A–F). Similar strategies have been used by de Jong and co-workers to understand performance trends in Fischer–Tropsch synthesis over Co- and Fe-based catalysts.^818–820^ These catalysts not only provide useful information on the most active and selective sites toward hydrocarbon and olefin formation, but also on structural evolution of the materials, as well as metal–support interactions.^821^

**Figure 27:**
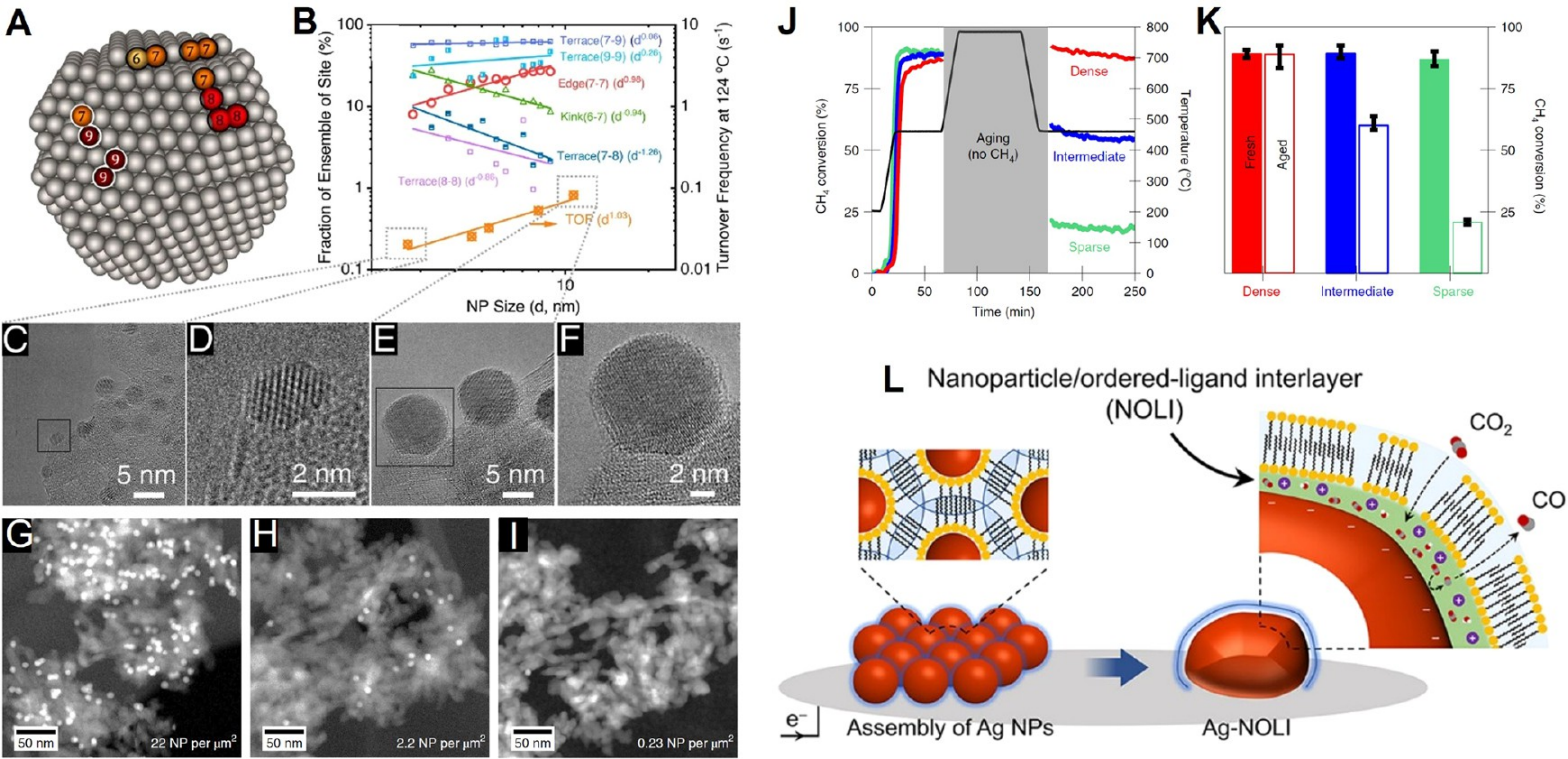
(A) Definition of the ensemble of sites on Pd/Pt NCs composed of 1500 atoms (∼4 nm) with color varying from yellow to red, indicating coordination numbers from low (6) to high (9). (B) Correlation between the simulated fractions of ensemble of sites and experimental turnover frequency at 124 °C with different NC size. (C–F) High-resolution TEM images of alumina-supported postcatalysis (C and D) 2.3 nm Pd/Pt NC and (E and F) 10.2 nm Pd/Pt NC. The boxes in (C) and (E) are used to highlight the high-resolution particles presented in (D) and (F), respectively. (G–I) Representative high-angle annular dark field scanning transmission electron microscopy images of dense (0.659 wt %) (G), intermediate (0.067 wt %) (H) and sparse (0.007 wt %) (I) Pd/Al_2_O_3_ samples where different loadings are used to change the Pd interparticle distance in the catalysts. (J) CH_4_ conversion profiles in methane oxidation for Pd/Al_2_O_3_ catalysts with different nanoparticle loadings following the temperature profile (black line and right axis). (K) Averaged CH_4_ conversion values at 460 °C for the Pd/Al_2_O_3_ catalysts before (‘Fresh’) and after (‘Aged’) aging. Error bars represent the minimum and maximum results of at least three repeat experiments. (L) Schematic illustration of nanoparticle/ordered-ligand interlayer (NOLI) formation and its effect in CO_2_ electrocatalysis. Surface ligands (tetradecylphosphonic acid) are initially covalently bonded to the nanoparticle (NP) surface. Upon biasing under CO_2_-reducing conditions, the ligands collectively detach and form a structurally ordered ligand layer. The starting small NCs also fuse into a larger NC during the process. The initial interaction between ligands induced by NC assembly is considered crucial for the NOLI formation. Panels (A–F) were adapted with permission from ref 817. Copyright © 2020 National Academy of Sciences. Panels (G–K) were adapted with permission from ref 838. Copyright © 2019 Goodman et al. under exclusive license to Springer Nature Limited. Panel (L) was adapted from ref 847. Copyright © 2021 American Chemical Society.

This latter topic is one where colloidal NCs excel given the opportunity to utilize the same NC precursors for the preparation of samples on different supports can easily be used as a strategy to identify the influence of the support on the catalytic performance.^822^ Finally, the use of colloidal NCs with specific defective structures (*e.g*., grain boundaries, point defects) can also help elucidate the role of these sites on activity, thus continuing to expand the reach of these materials in the fundamental understanding of catalytic performance.^823^

The past few years have witnessed the use of engineered colloidal catalysts to deliberately improve catalytic performance in several fields. The tunable nature of colloidal NCs endows them with the potential to achieve the largest fraction of active site motifs through tailoring their structure in solution. Alloyed and intermetallic NCs are clear examples of this approach, whereby mixing elements with complementary properties, *i.e*., one reactive element and one modulating its reactivity, catalysts with improved rates and selectivity can be obtained. Exemplary cases in electroreduction of CO_2_ to CO on ordered AuCu NCs^824^ and Pt/Sn intermetallic catalysts for propane dehydrogenation^825^ highlight this aspect, where the deliberate preparation of NCs with appropriate compositions allow to overcome activity and stability challenges in the reaction of interest. Pd-based bimetallic NCs have been utilized for the catalytic oxidation of methane. The study systematically shows the effects of adding a second metal to increase catalytic activity (for PdZn and PdNi) or to inhibit the sintering process (for PdSn, PdFe, and PdCo).^137^ In another paper, Ni–In NCs have been studied for catalytic hydrogenation of unsaturated aldehydes. Tuning the ratio between Ni:In as well as size of NCs, high catalytic activity and selectivity can be achieved. Eventually, 5 nm in size Ni_2_In NCs exhibit optimal performance.^826^ Intermetallic NCs offer opportunities for exploring alternative catalytic concepts, such as adsorption of homogeneous catalysts on the surface of NC,^827^ plasmonic catalysis,^828^ and electrocatalysis.^138^

Recent cases of more complex architectures built from colloidal NCs have been reported for the chemical upcycling of waste plastics. Perras and colleagues have shown that by astutely sandwiching Pt NCs between a nonporous silica core and a porous silica shell, it is possible to induce polymer melts to react with the Pt surface in specific conformations, leading to narrower distributions of hydrocarbon products in polyethylene hydrogenolysis.^829^

Another relevant example is the engineering of NC shapes to induce the formation of specific surface site motifs of relevance in several electrochemical transformations.^830,831^ The use of overlayers of oxides or other materials to cap and protect colloidal NCs has also emerged as an engineering tool to dictate selectivity and stability in heterogeneous catalysts, such as in the example of thermally stable catalysts for emission control applications using encapsulated Pt and Pd NCs in alumina,^832^ or in hybrid oxide coatings prepared in solution to enhance the stability of Cu-based catalysts for CO_2_ electroreduction.^833^ It is expected that this field of application of colloidal catalysts will continue to grow in the future.

Catalyst restructuring and activation/deactivation processes are somewhat unavoidable and often affect catalyst performance in crucial ways, and understanding these dynamic processes is an important step toward the identification of active sites and motifs in heterogeneous catalysts.^834,835^ Gaseous and liquid environments provide challenges for probing the most important factors responsible for processes like leaching, sintering, agglomeration, decomposition, and poisoning of active sites. Using uniform and well-defined colloidal catalysts therefore simplifies the study of catalyst dynamics because they reduce the number of variables that need to be considered when identifying their origin.

Harsh temperature and pressure conditions are usually associated with restructuring by ripening and particle migration that induce deactivation of supported catalysts. Using narrow size distributions allows to ascertain the contribution of particle size to the ripening/agglomeration phenomena, and colloidal catalysts can even allow to engineer these distributions and probe their involvement in deactivation processes.^836,837^ Controlling the dispersion and positioning of supported metal NCs allow demonstrating how the interparticle distance can affect particle decomposition processes that are responsible for deactivation of methane combustion catalysts through decomposition into single atoms (Figure 27G–K).^838^ The decomposition was found to be dependent on the NC density on the support, with denser (higher loading) catalysts being more stable than sparse (lower loading) catalysts, contrarily to what sintering deactivation phenomena would predict. In analogy to this work, more recent research prove how interparticle separation is crucial for enhancing catalytic selectivity in liquid-phase reactions by appropriately positioning supported metal particles within the catalyst layers.^839^

The advantage of colloidal NC approaches is that interparticle distance, metal loading, composition, can be independently tuned while guaranteeing similar catalytic properties of individual supported particles because those are encoded in the synthesis process. Changes occurring to the structure as well as the catalytic performance can easily be followed at the same time, and the changes can be very dramatic, as in the case of supported Ru NCs that go from producing methane to producing almost exclusively CO as a function of catalyst pretreatments in CO_2_ hydrogenation.^840^ The visualization of restructuring processes in real time can now be obtained using a combination of spectroscopic and microscopic measurements. Cu NCs used as electrocatalysts for CO_2_ reduction have been recently found to undergo redissolution and reprecipitation processes during activation, leading to changes in rates and selectivity for reduced products during operation and as a function of voltage cycles/operation.^841^ These examples highlight how colloidal NCs help identify and explain restructuring phenomena much more readily than with other catalyst synthesis techniques and provide a way to understand catalyst activation/deactivation phenomena.

One element that clearly distinguishes colloidal catalysts from other catalysts is the potential for taking advantage of the ligands/surfactants for imparting properties that would not otherwise be possible with other methods.^842^ In general, ligands/surfactants on the surface of colloidal NCs need to be removed after synthesis and before catalytic applications in order to allow the reactive surface of the NCs to be fully exposed to gaseous and liquid reactants.^843–845^ However, in certain cases, ligands can actually be utilized to direct the activity and selectivity of catalytic processes. In a particularly exciting example of utilizing ligand layer for reactivity, Kim, Yang, and co-workers demonstrate that a detached layer of ligands on the surface of Ag, Au, and Pd NCs can create an interlayer that can host cations and reactive species responsible for increased and highly selective CO_2_ electroreduction.^846^ Later on, it was found that ordered assemblies of NCs can also enhance these ligand interactions responsible for the improved performance, including the electrochemical stabilization of intermediates within the ordered organic ligand interlayers,^846,847^ leading to the formation of reservoirs of intermediates and reactive species (in this latter case mostly CO) that can facilitate the formation of higher carbon number products.^848^

The colloidal NC environment can be further engineered postsynthesis to drive the catalytic activity of the formed metal surfaces. Riscoe et al. hypothesized that coating NCs with polymer ligands can modify the reactivity of metal surfaces, and the development of NC-porous polymer hybrid materials first demonstrate that the polymer layers can dictate transition state stability in Pd-catalyzed CO oxidation reaction.^849^ Further engineering of the polymer layers and NC composition in Ru/TiO_2_ supported catalysts also demonstrated how the modification of the NC environment using functional groups in polymer layers can dictate the C–C coupling probability, largely increasing by few orders of magnitude the rate of higher hydrocarbon formation.^850,851^ The exploitation of surface ligands is an area of nascent exploration in the community that promises to further increase the utilization of engineered NCs in several catalysis application areas.

### NC-Based Precursors for Thermoelectric Materials

The conversion of heat into electricity and vice versa, known as thermoelectricity, presents a promising method for utilizing waste heat and optimizing thermal management.^852,853^ However, the high costs associated with expensive raw materials, energy-intensive manufacturing processes, and low efficiency have impeded the large-scale implementation of thermoelectric devices.^854^

Traditionally, thermoelectric materials are either done in single crystal form, high-temperature reactions, or by consolidating powders to produce dense ingots. Alternatively, powders can be synthesized in solution and constituted of NCs.^855^ Compared with solid-state methods, solution-processed thermoelectric materials demand shorter reaction times and lower temperatures, reducing energy consumption. Furthermore, NC reactions are intended to include self-purifying processes that should yield defect-free NCs of the targeted compound. Therefore, reagent purity requirements is less demanding as side products, unreacted species, and solvents can be separated after the synthesis.^856^ After the purification process, the NC-based powders are then consolidated into dense inorganic solids by applying pressure and temperature (Figure 28A).

**Figure 28:**
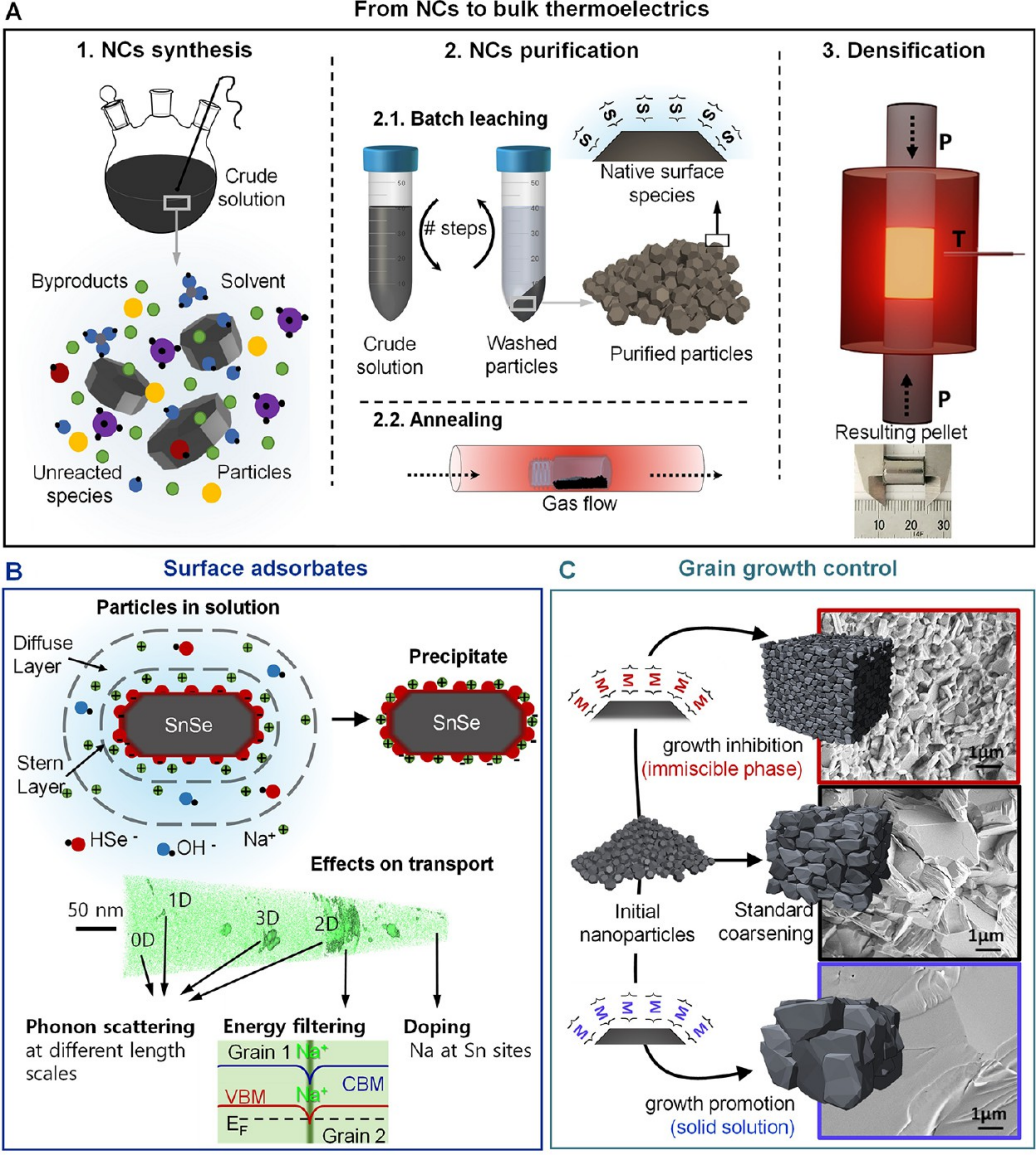
(A) Steps involved in the solution-processing of thermoelectric materials. Reproduced with permission from ref 856. Copyright © 2024 Fiedler et al. (B) Example of the role of NC surface adsorbates into the thermoelectric material microstructure. Reproduced with permission under CC BY 4.0 from ref 864. (Copyright © 2021 Liu et al. open access). (C) Example of grain growth control through NC surface ligands. Reproduced from ref 874. Copyright © 2021 American Chemical Society.

Beyond the potential reduced powder cost, solution synthesis offers pathways to create powders from precisely engineered NCs that can yield dense materials with features not achievable by other synthetic approaches.^857^ The reason is that by adjusting various controllable parameters when designing the powder (such as NC size, shape, crystal structure, composition, surface species, and organization), we can determine the final material features (such as crystal structure, defects, grain size, grain orientation, and interfaces), and as a result, the thermoelectric performance.^855^ In this scenario, NCs can act as versatile precursors that, when subject to the right reaction conditions, can develop into bulk materials with specific structural characteristics in a similar fashion as metal complexes are converted into well-defined NCs through colloidal synthesis.^858,859^

The development of this approach is still in its early stages, mainly due to the complex processes involved in transforming NCs into dense solids (*e.g*., sintering, decomposition of surface species, solid-state reactions, melting) and the extensive range of parameters that determine the final outcome. One of the most significant challenges is establishing clear correlations between the properties of the NCs and the structural characteristics of the consolidated material. A key issue arises from the requirement for comprehensive and accurate structural and compositional information about both the NC-based precursors and the resulting solid material, which is not only difficult but often time-consuming and requires specialized equipment not readily available in many laboratories.^855^

For example, NC composition usually differs from the nominal one, unlike traditional solid-state methods. Yet, minor changes in NC stoichiometry can massively impact the properties of the final solid. NC shape determines the surface atoms and their atomic environment, altering the particle’s surface energy and reactivity,^860^ both crucial parameters for the transformation that occurs during consolidation. Moreover, the surface species connected to the under-coordinated termination atoms can vary from molecules with long aliphatic chains to molecular or ionic species, each of them having very different roles during the transformation into dense solids.^861–864^

Typically, colloidal syntheses yield NCs capped with long hydrocarbon chains^863^ that form composites with a carbon-containing phase^843,865^ even after calcination of the ligands,^866,867^ potentially resulting in barriers for atomic diffusion^868^ and blocking charge transport.^869^ Alternatively, inorganic ligands can be used not only to avoid any carbon presence but also as a tool to further guide sintering and solid-state reactions during consolidation. Where the ligand could (i) become a crystalline inorganic matrix with the NCs^870,871^ embedded, (ii) react with the NCs to yield a new phase,^872^ (iii) control crystal growth^873–875^ and coarseing, (iv) transform into secondary phases,^874^ and (v) introduce atomic impurities.^866,876^

Functionalizing NC surface using inorganic molecules has proven significantly powerful in providing nanocomposites with features that cannot be achieved using the most established spinodal decomposition in high-temperature reactions.^866,868,877–879^ The inorganic molecules can be selected based on their role during sintering, which allows the control of the final materials’ structure and composition and, therefore, provides an additional tool to optimize thermoelectric performance. Recent examples have shown the possibility of controlling grain growth during sintering depending on the inorganic ligand. If the inorganic ligand decomposes into a compound that is miscible with the matrix phase, grain growth will be promoted, while if it is immiscible, it will be hindered (Figure 28C).^874,880^ Through hindering grain growth, SnSe-CdSe composites were produced with structural features that yield one of the highest-performance thermoelectric materials.^874^

Despite the importance of identifying surface species for rationalizing the process and the different methods to study the NC surface chemistry,^881^ some surface species can remain elusive. For example, observing ionic groups intercalating between organic molecules is complicated.^459^ Previously overlooked ionic adsorbates in SnSe NCs were unveiled by studying the resulting solid through atom probe tomography.^864^ When using Na salts in the reaction mixture, Na^+^ ions are electrostatically adsorbed on the NC surface and stay there after the NCs are purified to maintain charge neutrality.^864^ Na is present within the matrix in the sintered pellets, acting as a dopant in dislocations, precipitates, and forming grain boundary complexions. Moreover, it was proven that they play a crucial role not only in directing the material nano/microstructure during thermal processing but also in determining the transport properties of the consolidated material (Figure 28B).^864^ The interfaces created between SnSe and Na-rich phases lead to energy filtering, enhancing the Seebeck coefficient. These findings highlight the importance of a holistic analysis of the NC precursors and resulting materials to establish synthetic rules and have correct structure–property relationships.

In addition to the possibilities of tailoring the NC characteristics and their surface species, NC-based powders can be created using different types of NCs to form composites and even with a certain degree of ordering to direct the sintering process. Blending different types of NCs has been one of the most effective strategies for obtaining materials with exceptional properties that cannot be achieved through other methods. For instance, by blending Ag or Cu NCs with PbS, Ag/Cu-PbS nanocomposites could be produced with thermoelectric properties that surpassed any previously reported PbS-based material.^882,883^ This achievement was made possible thanks to the presence of long organic chains as ligands, which, upon annealing, converted into graphitic carbon, preventing the diffusion of Ag or Cu into the PbS matrix. The presence of metallic Ag/Cu, with a work function close to the conduction band of PbS, provided a way to control carrier concentration through modulation doping, allowing much higher mobility than the ionic doping method, resulting in high electrical conductivities throughout the entire temperature range.^882^

NC blends can also be conceived to induce reactions that yield different materials from the original NCs. An example is the combination of CsPbBr_3_ and PbS NCs.^884^ During the consolidation process under temperature, PbBr_2_ is extracted from CsPbBr_3_ and diffuses into the PbS matrix, controlling the material doping level, as Br^–^ acts as an electron donor, leaving behind Cs_4_PbBr_6_ nanodomains.^884^

In the past decade, significant efforts have been made not only to unravel the unknowns of NC-based precursors to produce dense inorganic material^856,864,885,937^ but also to use them to reach record thermoelectric performance while trying to minimize cost.^857,874,886,887^ In this line, NC-based precursors have also gained lots of traction as active materials for the production of thermoelectric materials through additive manufacturing techniques.^888,889^

### Printed Thermoelectric Devices from NC-Dispersed inks

Conventional bulk-scale thermoelectric modules consist of *n*-type and *p*-type cuboid-shaped thermoelectric semiconductor legs connected electrically in series and thermally in parallel. They are typically manufactured through bulk-scale processing techniques that involve multistep processes such as synthesis of ingots, dicing, metallization, and chipping.^890^ However, these traditional methods are not only energy-intensive and expensive but also limit the flexibility in module design. This issue becomes particularly critical when fabricating emerging thermoelectric devices such as cylindrical, miniatured, and wearable devices. Microscale miniatured devices offer the potential to be used as auxiliary power supply or local thermal management devices for electronic systems such as the Internet of Things, wireless sensor networks, and lab-on-a-chip devices.^891^

Recently, various printing techniques have gained attention for fabricating thermoelectric devices with customized designs by patterning *n*-type and *p*-type thermoelectric semiconductors and metal electrodes.^892,893^ Compared to conventional manufacturing techniques, printing techniques offer several advantages, including enhanced design flexibility in device structures and cost-effectiveness in processing. Colloidal NCs have emerged as a highly effective ink for these printing processes. They can be synthesized via well-established methods, and their surface chemistry enables the optimization of ink printability. Additionally, the intrinsic thermoelectric properties of the printed materials can be enhanced by controlling the size, composition, and surface ligands of the NC building blocks.

For printing with thermoelectric NC inks, the colloidal stability of the NC inks must be secured, and their rheological properties adjusted to conform with the selected printing method. The colloidal stability of NCs is typically achieved through the utilization of surface ligands. For instance, long-chained organic surfactants or polymers are frequently employed for enhancing the colloidal stability of NCs through steric stabilization. However, these organic stabilizers, which are generally electrical insulators, can potentially introduce impurities that can degrade the thermoelectric properties of the final product.^894,895^ Consequently, post-thermal or chemical processing steps may become necessary to eliminate these organic impurities. Alternatively, electrostatic stabilization of NCs presents an effective approach to securing colloidal stability while minimizing the presence of undesired impurities. For instance, all-inorganic NCs coated with molecular metal chalcogenide complexes, also known as chalcogenidometallates, are highly soluble in polar solvents. Furthermore, upon heating, they can induce phase transformations in semiconducting metal chalcogenides.^554,867,896,897^ This feature can be leveraged to control their composition and doping.

The rheological properties of inks are of paramount importance in ensuring the printing processability. For instance, the success of screen printing relies on highly viscous inks, whereas inkjet printing necessitates inks with low viscosity.^892^ Aerosol jet printing accepts a wide range of ink viscosities.^892^ However, Newtonian rheological properties are often required to achieve a uniform deposition of inks. Optimizing these rheological properties for inks containing NCs is generally achievable by adjusting the volume fraction of NCs within the medium. However, enhancing the viscosity of colloidal NC inks can sometimes be challenging due to solubility limitations. One approach to address this challenge is to introduce surface charges on the NC, which creates an electric field near the charged surface. This induced electric field alters the structure of the surrounding fluids and the rheological properties of the NC-dispersed inks via the electroviscous effect.^898–902^ Kim et al. developed all-inorganic viscoelastic thermoelectric inks for dispensing-based 3D printing by using Sb_2_Te_4_^2–^ chalcogenidometallate anions as inorganic additives for Bi_2_Te_3_-based NCs (Figure 29A). Employing compositionally similar anions has proven to be beneficial in enhancing the viscoelasticity of the NC-dispersed inks and maintaining the compositional integrity of the final printed product.

**Figure 29:**
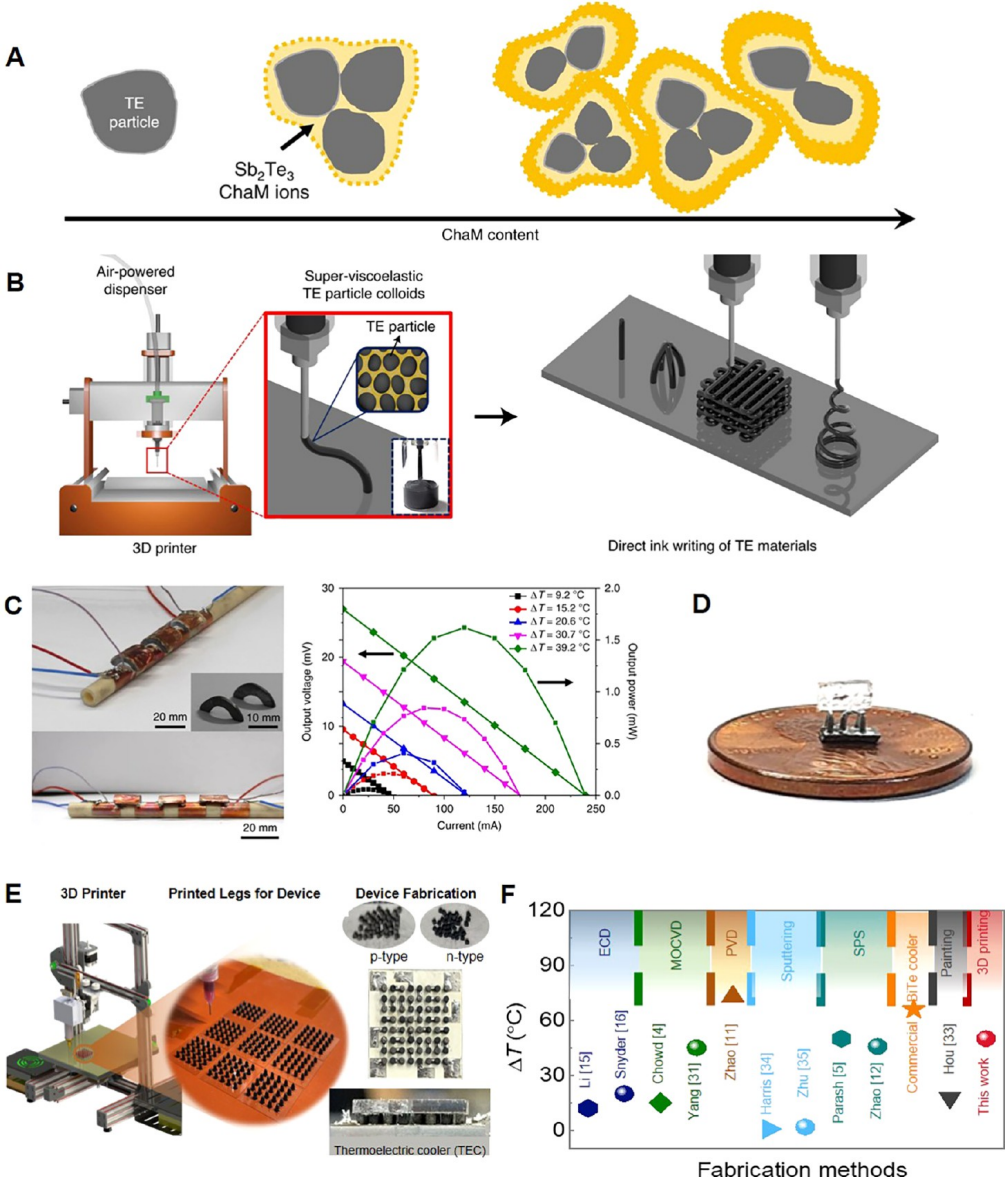
(A) Scheme of the formulation of all-inorganic thermoelectric-NC-based inks. (B) Scheme of the direct 3D writing process of thermoelectric NC-based inks with high viscoelasticity. (C) Cylindrical power generators that consist of 3D-printed *p*-type and *n*-type thermoelectric half-rings and its output voltage and power depending on temperature differences. (D) Microscale thermoelectric devices fabricated by the direct 3D writing process. (E) Schematic diagram of the used 3D printer with a picture of the printed thermoelectric legs and their assembly into the device. (F) Comparison of the maximum cooling gradients achieved for thermoelectric coolers fabricated by different methods. Panels (A) and (C) were reproduced with permission from ref 898. Copyright © 2018 Kim et al. Panels (B) and (D) were reproduced with permission from ref 903. Copyright © 2021 Kim et al. under exclusive license to Springer Nature Limited. Panels (E) and (F) were reproduced with permission from ref 907. Copyright © 2025, The American Association for the Advancement of Science.

Another key parameter is the characteristics of NCs. Highly viscoelastic thermoelectric inks have been developed by optimizing the particle size, size distribution, and surface states of the inks, facilitating direct 3D printing using the inks (Figure 29B).^903^ This process can produce single filaments with lateral dimensions on the order of hundreds of micrometers, which can be used as thermoelectric legs in microscale thermoelectric devices.

Ink printing has been used to manufacture devices using well-known thermoelectric materials of Bi_2_Te_3_, Bi_2‑x_Sb_*x*_Te_3_, PbTe, and Cu_2_Se as inks. The NC surfaces in the Bi_2_Te_3_ and Bi_2‑x_Sb_*x*_Te_3_ inks were engineered using Sb_2_Te_4_^2–^-based ions, while those in the Cu_2_Se ink were modified using Se_8_^2–^ polyanion as a surface capping agent. Lee et al. enhanced the viscoelasticity of PbTe inks by electronically doping them with Na and Sb, which induced surface charges in the inks through surface-charge imbalance. Efficient particle sintering was achieved using these inks, which increased the peak *ZT* values of the Bi_2‑x_Sb_*x*_Te_3_, PbTe, and Cu_2_Se products up to 1.1, 1.4, and 2.0, respectively. These ZT values are comparable to those obtained for typical ingots with same compositions.^899,901,902,904^

Ink printing technology uniquely enables the fabrication of thermoelectric devices with customized shapes, intricate structures, and microscale dimensions (Figure 29C,D), offering a level of flexibility not attainable through traditional manufacturing processes. For instance, studies have demonstrated the efficiency of dispensing-based 3D printing and aerosol jet printing technologies in fabricating heat-source conformable thermoelectric devices.^898,899,905^ These methods enable the production of complex architectural designs and the precise deposition of inks on curved surfaces. Another distinct advantage of printing technology is its versatility: materials can be printed on a diverse range of substrates. Moreover, the thickness of the printed thermoelectric materials can be controlled using this technology. This adaptability allows for the efficient and straightforward manufacturing of flexible thermoelectric devices catering to wearable applications.^906^ The streamlined printing process and meticulous control over material utilization significantly enhance production efficiency and cost-effectiveness, ultimately expanding the market for thermoelectric devices. Very recently, Xu et al.^907^ reported the fabrication of high-performance thermoelectric materials (*p*-type bismuth antimony telluride [(Bi, Sb)_2_Te_3_] and *n*-type silver selenide (Ag_2_Se)) with a record-high cooling temperature gradient of 50 °C in an ambient environment by using an extrusion-based 3D printing technique (Figure 29E,F). By tailoring the ink formulation, Xu et al. demonstrate the formation of interfacially bonded grains, allowing high mobilities despite the presence of a large number of pores. This resulted in 3D-printed materials with excellent thermoelectric properties.

### Phase Change Memory (PCM) at the Nanoscale

Currently, our computers are built by combining several silicon technologies to reach an intricate optimum for the cost, speed, and density of microchips.^908^ One problem of such a computer architecture is the pronounced mismatch between the speed of slow but nonvolatile storage-class memory (*e*.*g*., NAND Flash SSD) and fast but volatile operating memory (*e.g*., Dynamic RAM). This speed gap can extend beyond 1–2 orders of magnitude, rendering operating memory idle upon slow data transfer from the memory-storage segment of the computer and thus limiting the computer performance, power efficiency, and upscaling of computer clusters.^909^ PCM devices are ideal for closing the speed gap as their performance can be tuned between the DRAM and NAND characteristics by the choice of PCM material.^910,911^ Besides solving one of the most pressing challenges in computer architecture, PCM technology features favorable and well-differentiated performance parameters, including a bandwidth up to 5 GB/s, subns switching rate, and improved data retention at ambient and elevated temperatures.^912^

The two main challenges of PCM technology are the high price per bit and the further miniaturization of memory devices. The future development of the PCM field thus belongs to nanoscience, and specifically, liquid-based chemistry offers an all-embracing approach to improve PCM technology.^913,914^ Monodisperse colloidal nanoparticles are ideal for studying scaling rules for ultrasmall PCM devices, disentangling intertwined effects of structure at the nanoscale and a contribution of surface atoms.^915–918^ Furthermore, liquid-phase clusters and complexes^919,920^ unlock a convenient materials platform for screening PCM materials and enabling alternative fabrication approaches for PCM devices, such as spin-coating and printing, which are less expensive, more material-efficient, and template-independent.^921–923^ Figure 30 summarizes the status of liquid-phase PCM nanomaterials.

**Figure 30:**
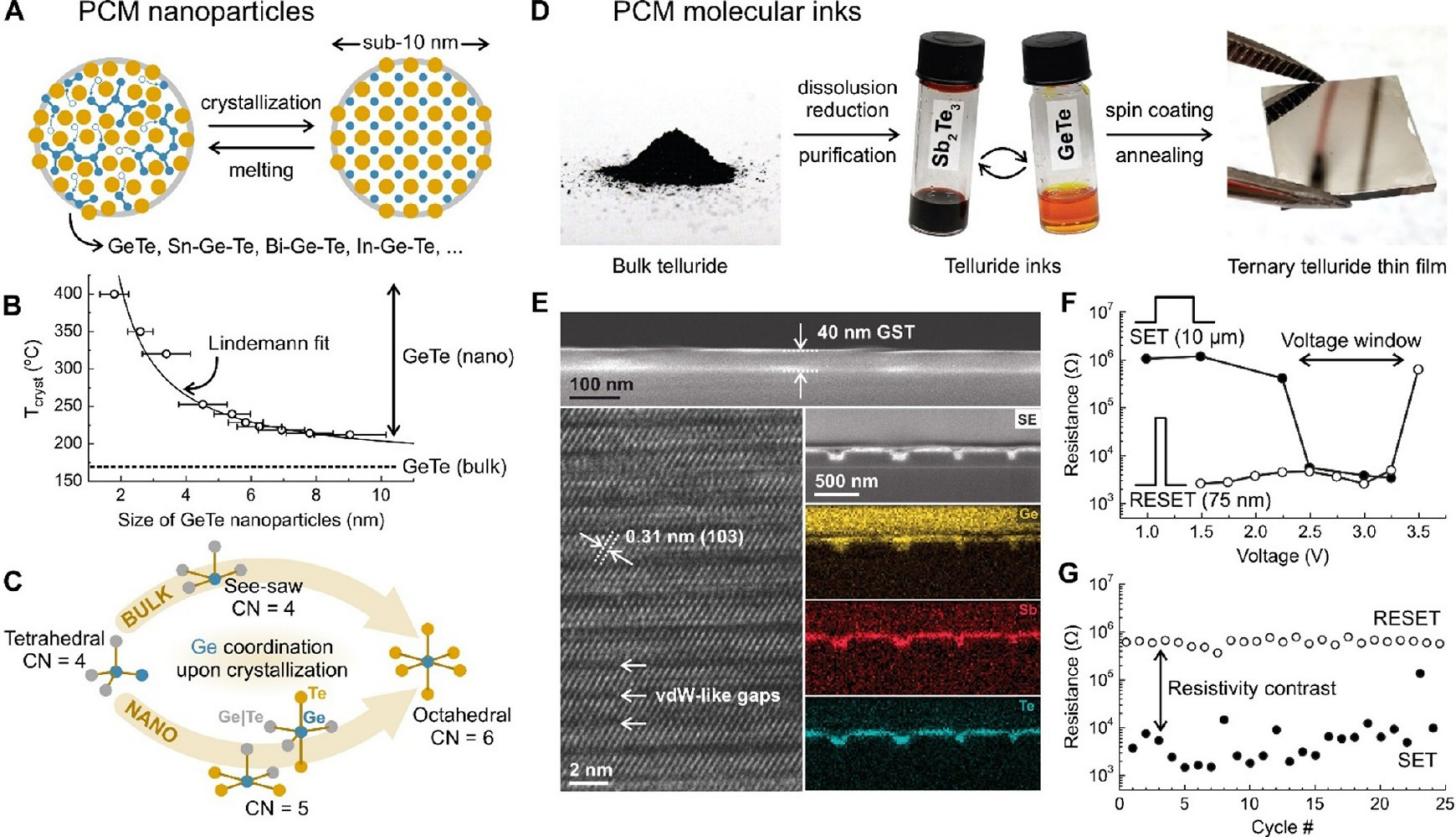
(A) Schematics of reversible phase transitions between amorphous and crystalline PCM nanomaterials. Note the chain ordering of cations (blue) in the amorphous structure. (B) Size-dependent crystallization temperature of GeTe nanoparticles and a Lindemann criterion fit of the dependence. (C) Crystallization mechanism bulk vs nanoscale GeTe PCM material. (D) Schematics for the thin film deposition from telluride molecular inks. (E) Structural properties of ink-based Ge–Sb–Te (GST) thin films. (F, G) Switching and cycling of a PCM device with solution-engineered GST memory layer by tuning the amplitude and duration of the voltage pulses. Note the voltage window in F and the resistivity contrast in G for reliable switching and reading of a memory cell, respectively. Panels (A) and (C) were reproduced with permission under CC BY 4.0 from ref 928. (Copyright © 2024 Wintersteller et al. open access). Panel (B) was reproduced with permission from ref 916. Copyright © 2018 American Chemical Society. Panels (D)-(E) were reproduced from ref 922. Copyright © 2023 American Chemical Society.

#### PCM Nanoparticles

PCM technology is based on crystallization and melting phase transitions (Figure 30A), switching the memory material between amorphous and crystalline phases with distinctly different electrical (*i.e*., resistivity) and optical (*i.e*., reflectivity) properties.^911^ In contrast to bulk materials, however, nanoparticles attain a size dependency for both phase transitions.^914^ For example, it has been consistently observed by several groups that the crystallization temperature of GeTe nanoparticles increases as their size decreases (Figure 30B).^915,916,924,925^ This phenomenon has been explained by the effect of surface atoms, holding excessive energy per atom and thus rendering the structure of the nanoparticle more disorganized compared to the bulk. The analogy can be taken from the melting point depression phenomenon,^926^ except that the crystallization phase transition is characterized by increased ordering (*i.e*., negative change of entropy), which is the opposite to the melting phase transition.^916^ Therefore, the surface plays the opposite effect too, requiring higher crystallization temperatures for materials with larger fraction of surface atoms (*i.e*., smaller sizes). In common, both phase transitions can be quantified by the Lindemann criterion, as shown in the case of GeTe crystallization in Figure 30B.^916^

The size-dependent phase transitions are the game changers for the ultrasmall PCM devices, affecting all phase change properties. For example, higher crystallization temperatures lead to better data retention properties, while low melting temperature improves the power consumption characteristics of PCM devices.^916^ On the flip side, however, the bonding and structural dynamics are also affected.^927^ Specifically, PCM NCs exhibit stronger glass-forming properties, leading to slower crystallization kinetics and stronger covalent bonding.^928^ Therefore, the crystallization mechanism of GeTe nanoparticles includes an additional transition state for Ge atoms with the coordination number of 5 as they are changing the amorphous tetrahedral environment to the rock-salt-type octahedral coordination (Figure 30C).^928^ Although this nanoscale effect leads to slower switching, it appears beneficial in suppressing the aging of PCM devices, which is particularly useful for multibit data storage and analog-type computing applications. In summary, while only several telluride PCM compositions have been so far developed in the form of size-uniform colloidal nanoparticles,^917,918^ it will be important to extend this library of PCM nanomaterials in order to test the generalizability of reported nanoscale phase-change effects.^915,916,924,929^

#### PCM Molecular Inks

Bulk tellurides can be dissolved in several solvents, including hydrazine chemistry and, more recently, a cosolvent formulation of diamine and dithiol.^919,920^ Upon reduction and purification steps, bulk telluride powders are broken down to molecular complexes and small clusters,^930^ which are easy to spin coat on a variety of substrates (Figure 30D). Such thin films show excellent structural characteristics, including small roughness, thickness tunability, compactness, and high crystallinity. Furthermore, telluride inks can be deposited on prepatterned substrates, filling grooves and vias with small lateral dimensions (Figure 30E).^922,923^ Therefore, molecular telluride inks are a convenient materials platform for PCM technology (Figure 30D).

Recently, a proof-of-concept memory device with an ink-based PCM layer has been demonstrated.^922^ The telluride ink can be drop-casted or spin-coated to infill the via opening and thus connect two buried planar electrodes. This device is then switched by tuning the amplitude and the length of the voltage pulse (Figure 30F), and the switching can be repeated many times while both resistivity states remain nonvolatile (Figure 30G). While this memory device shows several key properties, such as distinct resistivity contrast, cyclability, and low power consumption, further improvement of endurance and aging properties of liquid-borne PCM devices are necessary to reach the standards of sputtered PCM chips.^912^ In addition, the possibility to achieve sub-10 nm memory cell dimensions will become the key for widespread integration of ink-based PCM layers in the fabrication processes of memory chips.

## Conclusions and Outlook

In the past decade, the landscape of colloidal NC research has undergone a transformative evolution, pushing the boundaries of nanoscience and technology with breakthroughs that seemed unthinkable just a few years ago. These tiny structures have transcended their origins as lab curiosities to become pivotal components in fields as diverse as energy harvesting, optoelectronics, catalysis, and quantum information science.

Exquisite control over the size and shape of NCs of various metals, semiconductors, magnetic, and upconverting materials has been achieved. Some of these materials, such as luminescent QDs, are nowadays synthesized on an industrial kg-scale for display applications. At the same time, oxide nanoparticles are used in sunscreens, composites, and other products. NC syntheses have been extended to material systems with ever-increasing complexity. The latter includes randomized complex compositions, yielding high configurational entropy in high-entropy materials (HEMs), chiefly metals and metal oxides. The other exciting avenue toward highly entropic materials is to leverage extreme structural dynamics, as in metal-halide perovskite NCs. These QDs have challenged the ethos of the field, whereby the antibonding valence band and overall structural softness give rise to an unexpectedly clean electronic behavior, coined as defect tolerance.

One major focus has been on unveiling mechanisms that underpin the NC formation, with the ultimate goal of devising universal synthesis approaches. Adapting the concept of retrosynthesis from organic chemistry, the idea is to decompose the target NCs into simpler building blocks, mapping out the chemical pathways, reaction conditions, and reagents required. Improved *in situ* characterization techniques, providing real-time information on the NC formation process, emerge as crucial tools to obtain the much sought-after molecular-level understanding. Such studies are currently challenging established models of the NC growth and increasingly suggest that nonclassical nucleation is a rather common NC formation path.

NC self-assembly continues to trigger the imagination as a powerful, yet delicate, path to devise advanced materials. Notable strides have been made in the shape-controlled NC assembly and the development of *in situ* electron microscopy and X-ray scattering methods as accompanying diagnostic tools. Liquid-phase TEM, allowing real-time observation of NC dynamics at the nanoscale, has revealed complex, nonclassical crystallization pathways and intermediate phases during NC self-assembly. Despite the progress, challenges remain in minimizing artifacts from electron-beam interactions, better understanding the hydrodynamic properties of liquid-cell TEM systems, and replicating *ex situ* self-assembly conditions.

The remarkable innovations in surface chemistry have been the gateway to the development of NC-based gels, 2D patterning, and 3D printing of NCs. The assembly of NC gels has enabled the construction of porous networks with remarkable versatility in applications. Dynamic bonding between the particles renders the gels reconfigurable and responsive to diverse stimuli, such as temperature or chemical changes. There are abundant possibilities when fine-tuning NC gel characteristics and harnessing their capabilities. However, creating spatially uniform, stable equilibrium gels with NC building blocks still presents a major challenge.

High-fidelity direct optical lithography of NCs into high-resolution 2D patterns has been accomplished through photochemically active ligands or linkers. This advance has enabled integration of NCs into complex and multilayered device architectures. The major undertakings for the years to come are to further improve spatial resolution, especially below the 100 nm scale, optimize NC stability under various processing conditions, and expand the range of materials that can be patterned with these techniques.

Beyond 2D patterning, the programmable assembly of 3D structures with nanoscale resolution has been accomplished by using photosensitive ligands or molecular additives. Semiconductor, metal, and metal-oxide NCs have been printed into 3D functional structures through light-induced chemical reactions, forming bonds between the NCs through their capping ligands. NC 3D printing has brought the vision of fully 3D printing all the device components closer to reality. However, there are still considerable hurdles in achieving high material purity, strong mechanical integrity, and efficient charge and exciton transport in printed NC structures.

In the last four decades since the conception of colloidal QDs, optoelectronic applications have been at the forefront of QD research, including displays, LEDs, lasers, photovoltaics, and IR imaging and sensing. Successful large-scale commercialization has already been achieved for InP-based QDs (*e.g*., by Samsung), CdSe-based QDs (*e.g*., by TCL), and lead-halide-perovskite-based QDs (*e.g*., by Avantama), particularly in the context of display applications, where such QDs enable wide color gamut, high brightness, and high energy efficiency. Presently, increasing research activities are foreshadowing near-term market entry also for several other NC material platforms, such as Zn-chalcogenides QDs (as blue emitters, *e.g*., for LEDs). However, several challenges remain in advancing the commercial competitiveness of QD-based light-emitting applications, including addressing the toxicity of some QD materials, improving their long-term device stability and efficiency, especially for blue-emitting QDs and when incorporated in LEDs and lasers, and scaling up production while maintaining quality.

In the wake of the prominent QD display and LED developments, QD lasing has also been blessed with substantial progress in the past decade. Among the noteworthy recent developments is ASE in electrically stimulated colloidal QDs, bringing the field closer to realizing electrically pumped laser diodes. Future work should focus on achieving stable and efficient electrically driven QD lasing for practical applications, particularly in the NIR spectral region for telecommunications and integrated photonics.

More limited progress has been made with QD-based solar cells, which were once at the epicenter of NC research as a potential cost-effective alternative to the established semiconductor thin-film technology. However, the slow progress in efficiency, problems with stability, and concerns regarding production scalability, all accompanied by strong competition from emerging technologies, have diluted researchers’ efforts and turned the attention to alternative photovoltaic materials like (bulk) perovskite thin films, providing comparable benefits with fewer limitations.

Emerging as a more promising light-absorbing technology are colloidal QD photodetectors, especially in the field of IR imaging and sensing, with lead and mercury chalcogenide QDs as the main contender in industry, commercialized, *e.g*., by Emberion^931^ and Quantum Solutions.^931^ These detectors now cover a broad spectral range, extending from the visible to the SWIR and mid-IR regions, with enhanced quantum efficiency and detectivity, rendering them competitive with traditional semiconductor devices regarding sensitivity and noise performance. Combined with the ability to produce large-area sensors at low cost, QDs hold the promise to induce a paradigm shift in SWIR detection. Future research will focus on ensuring long-term stability, reducing noise levels, developing scalable fabrication techniques, and minimizing the adverse environmental impact of heavy-metal-based QDs.

Transcending the many classical optoelectronic applications studied early on, the past years have also seen colloidal QDs emerge as a promising candidate for quantum-light applications, either as a solution-processed quantum-light sources on their own, or as a controllable platform for hosting quantum-point defects with exciton and spin qubits. Similar to epitaxial QDs, the size-confinement effects in colloidal QDs facilitate single-photon emission, while the mature and versatile wet-chemical fabrication methods also allow fine control over the emission color and bestow solution processability. Single-photon purity at room temperature and high emission rates render InP and lead-halide perovskite QDs suitable for practical quantum applications. However, to successfully compete with various alternative quantum-light material platforms, massive efforts will be required. The community may further enhance the single-photon purity, spectral stability, and coherence times, as well as fully leverage their solution-processability as a powerful feature enabling versatile approaches for QD integration into nanophotonic device schemes.

In the realm of quantum science, colloidal NCs, with their ability to host quantum point defects, such as NV-centers, may enable the creation of qubits with both short-lived excitons for photon generation and long-lived spin states for quantum memory. However, several questions require attention; *inter alia*, the impact of surface states on quantum properties and the integration of NCs with existing technologies.

NCs have been pivotal players in catalytic applications due to their large surface-to-volume ratio and have gained significant attention due to their tunable size, shape, composition, and surface properties. These precise features present opportunities to control catalytic activity, selectivity, and stability, and to understand catalytic mechanisms at a fundamental level. Despite their potential, the stability of these materials under harsh catalytic conditions, such as high temperatures and pressures, is a concern. Engineering the NC-ligand interface and developing robust surface chemistries are critical areas for future research. Such efforts, both for metal- and semiconductor-NC catalysis platforms, may profit from developing suitable *in-operando* and single-particle-level characterization methods to obtain the mechanistic insights needed to formulate rational-design strategies.

Using NCs as precursors presents a promising alternative to traditional synthetic methods of thermoelectric materials by leveraging precise control over the NC’s properties, such as size, shape, and composition, to tailor the resulting material composition and microstructure. However, converting NC-based powders into dense, high-performance thermoelectric solids is challenging. The transformation involves complex processes requiring further research to understand and rationalize fully. While these significant questions are being answered, the ability to produce cost-effective, high-performance thermoelectric materials with customizable shapes and structures, especially through advanced techniques like 3D printing, makes NCs a compelling option for next-generation thermoelectric devices.

Integrating colloidal NCs into PCM technology allows for precise control over the size and distribution of memory materials. Size-dependent phase transitions can improve the energy efficiency and switching speed of PCM devices, making them suitable for applications requiring rapid data access.

Although this perspective has not delved into biomedical applications of colloidal NCs, it is important to acknowledge their significant impact in this field. NCs have been employed in drug-delivery systems to improve the solubility and bioavailability of poorly soluble drugs, allowing for controlled and targeted release. In imaging, QDs and other luminescent NCs have facilitated high-resolution cellular and *in vivo* imaging, providing deeper insights into biological processes at the molecular level. Additionally, NCs have found applications in biosensing, enabling ultrasensitive detection and theranostics, combining diagnosis and treatment in a single platform. For those interested in exploring the biological applications of colloidal NCs, we defer them to more bio-focused perspectives and reviews.

Finally, we suggest that the coming decade of nanoscience with NCs will see a further surge in computational tools for accelerated materials discovery, advanced materials characterization, and device optimization. While currently not yet as established as in fields such as protein design and protein structure prediction,^932^ an increasing number of NC researchers incorporate artificial intelligence (AI) tools into their everyday research endeavors, such as machine-learning (ML) algorithms, and increasingly also deep-learning methods. In the field of NC synthesis, ML methods may accelerate the tedious exploration of vast and often still poorly explored synthesis parameter spaces to achieve specific NC properties and functionalities.^933^ Such efforts are underway at various levels: many traditional human-centered research laboratories are incorporating simple tools to harness the existing low-data-volume data sets from either existing literature^934^ or their own research laboratories;^22^ simultaneously, few research groups are also starting to build and operate full-fledged robotic synthesis-and-characterization platforms for colloidal NCs, leveraging high-throughput experiments and deep-learning algorithms based on multilayered neural networks.^935,936^ In the field of NC characterization, an advantage is particularly expected for methods handling huge data volumes from multidimensional data sets, such as those generated from *in situ* or *operando* TEM, synchrotron-based studies, shot-to-shot analyses in laser spectroscopy, or real-time monitoring of device performance. In the field of computational chemistry, ML will bring a long-term target closer to reality: the accurate simulation of NCs with realistic size, shape, and ligand passivation over sufficiently long time scales, as required, *e.g*., to quantify and rationalize charge/energy transport, charge/energy recombination, or chemical reactions. For example, machine-learned force fields may achieve the high computational accuracy of quantum-chemical methods at the much-reduced computational cost of classical methods. Given the pace at which AI is entering our everyday life inside and outside research laboratories, it may not be easy to predict precisely how, but certainly that, AI will impact the coming decade of nanoscience with NCs.

In conclusion, the progress made in colloidal NC research over the past decade has been truly remarkable, paving the way for innovative applications across various fields. The success of NC research is a testament not only to the perseverance of individual researchers but also to the vibrant, interdisciplinary collaborations that have driven (and will continue to shape) this field. These cross-disciplinary efforts, along with computational aid from techniques such as AI, are the only way to continuously stream breakthroughs and innovations in nanoscience with NCs.

## Abbreviations

NC: nanocrystal
QD: quantum dot
PL: photoluminiscence
QY: quantum yield
TOPO: trioctylphosphine oxide
TOP: trioctylphosphine
NMR: nuclear magnetic resonance
XRD: X-ray diffraction
CNT: classical nucleation theory
NPL: nanoplatelet
NR: nanorod
UV–vis: utraviolet visible
UV: ultraviolet
SAXS: Small-angle X-ray scattering
2D: two-dimensional
ZB: zinc blende
ML: monolayer
1D: one dimension
3D: three-dimensional
IR: infrared
NIR: near-infrared
SWIR: short-wave infrared
LED: light emitting diode
MWIR: midwave infrared
ITO: indium tin oxide
BIC: bound states in the continuum
HEM: high-entropy material
HEA: high-entropy alloy
MR: metal-rich
M_*x*_B_*y*_: metal boride
B: boron
FA: formamidinium
MA: methylammonium
AZ: aziridinium
RP: Ruddlesden–Popper
SEM: scanning electron microscope
TB: tight binding
FF: force field
ML-FFs: machine learning-based force fields
SL: superlattices
DOLFIN: direct optical lithography of functional inorganic nanomaterials
PEB: photoexcitation-induced chemical bonding
MPA: 3-mercaptopropionic acid
ROIC: read-out integrated circuit
CMOS: complementary metal-oxide-semiconductor
ASE: amplified spontaneous emission
QLD: QD laser diodes
EL: electroluminescent
*j*: high current densities
BRW: Bragg reflection waveguide
DBR: distributed Bragg reflector
TLS: two-level system
SPE: single-photon emitter
SF: superfluorescence
NV-: nitrogen vacancy
AFM: atomic force microscope
PCM: phase-change memory
CM: carrier multiplication
SE: spin exchange
TA: transient absorption
AI: artificial intelligence
ML: machine learning
